# Discovery of Novel Coumarin-Schiff Base Hybrids as Potential Acetylcholinesterase Inhibitors: Design, Synthesis, Enzyme Inhibition, and Computational Studies

**DOI:** 10.3390/ph16070971

**Published:** 2023-07-06

**Authors:** Aso Hameed Hasan, Faruq Azeez Abdulrahman, Ahmad J. Obaidullah, Hadil Faris Alotaibi, Mohammed M. Alanazi, Mahmoud A. Noamaan, Sankaranarayanan Murugesan, Syazwani Itri Amran, Ajmal R. Bhat, Joazaizulfazli Jamalis

**Affiliations:** 1 Department of Chemistry, Faculty of Science, University Teknologi Malaysia, Johor Bahru 81310, Johor, Malaysia; aso.hameed@garmian.edu.krd; 2 Department of Chemistry, College of Science, University of Garmian, Kalar 46021, Kurdistan Region, Iraq; 3 Department of Pharmacy, Kalar Private Technical Institute, Kalar 46021, Kurdistan Region, Iraq; farouqpharma85@gmail.com; 4 Department of Pharmaceutical Chemistry, College of Pharmacy, King Saud University, Riyadh 11451, Saudi Arabia; aobaidullah@ksu.edu.sa (A.J.O.); mmalanazi@ksu.edu.sa (M.M.A.); 5 Department of Pharmaceutical Sciences, College of Pharmacy, Princess Nourah bint AbdulRahman University, Riyadh 11671, Saudi Arabia; hfalotaibi@pnu.edu.sa; 6 Mathematics Department, Faculty of Science, Cairo University, Giza 12613, Egypt; noamaan@sci.cu.edu.eg; 7 Medicinal Chemistry Research Laboratory, Birla Institute of Technology & Science Pilani (BITS Pilani), Pilani Campus, Pilani 333031, Rajasthan, India; murugesan@pilani.bits-pilani.ac.in; 8 Department of Biosciences, Faculty of Science, Universiti Teknologi Malaysia, Johor Bahru 81310, Johor, Malaysia; syazwaniitri@utm.my; 9 Department of Chemistry, R.T.M. Nagpur University, Nagpur 440033, Maharashtra, India; bhatajmal@gmail.com

**Keywords:** acetylcholinesterase, Schiff base, coumarin, DFT, chemical reactivity, molecular modeling, drug likeness

## Abstract

To discover anti-acetylcholinesterase agents for the treatment of Alzheimer’s disease (AD), a series of novel Schiff base-coumarin hybrids was rationally designed, synthesized successfully, and structurally characterized using Fourier transform infrared (FTIR), Nuclear magnetic resonance (NMR), and High-Resolution Mass Spectrometry (HRMS) analyses. These hybrids were evaluated for their potential inhibitory effect on acetylcholinesterase (AChE). All of them exhibited excellent inhibitory activity against AChE. The IC_50_ values ranged from 87.84 to 515.59 μg/mL; hybrids **13c** and **13d** with IC_50_ values of 0.232 ± 0.011 and 0.190 ± 0.004 µM, respectively, showed the most potent activity as acetylcholinesterase inhibitors (AChEIs). The reference drug, Galantamine, yielded an IC_50_ of 1.142 ± 0.027 µM. Reactivity descriptors, including chemical potential (μ), chemical hardness (η), electrophilicity (ω), condensed Fukui function, and dual descriptors are calculated at wB97XD/6-311++ G (d,p) to identify reactivity changes of the designed compounds. An in-depth investigation of the natural charge pattern of the studied compounds led to a deep understanding of the important interaction centers between these compounds and the biological receptors of AChE. The molecular electrostatic surface potential (MESP) of the most active site in these derivatives was determined using high-quality information and visualization. Molecular docking analysis was performed to predict binding sites and binding energies. The structure-activity-property relationship studies indicated that the proposed compounds exhibit good oral bioavailability properties. To explore the stability and dynamic behavior of the ligand-receptor complexes, molecular dynamics simulations (MDS) were performed for 100 ns on the two best docked derivatives, **13c** and **13d**, with the AChE (4EY7) receptor. A popular method for determining the free binding energies (MM/GBSA) is performed using snapshots taken from the systems’ trajectories at 100 ns. These results revealed that the complex system of compound **13d** acquired a relatively more stable conformation and exhibited better descriptors than the complex system of compound **13c** and the Galantamine drug, suggesting its potential as an effective inhibiting drug. The binding free energy analysis revealed that the 13d-4EY7 complex exhibited greater stability with AChE receptors compared to other complexes.

## 1. Introduction

**Figure 1:**
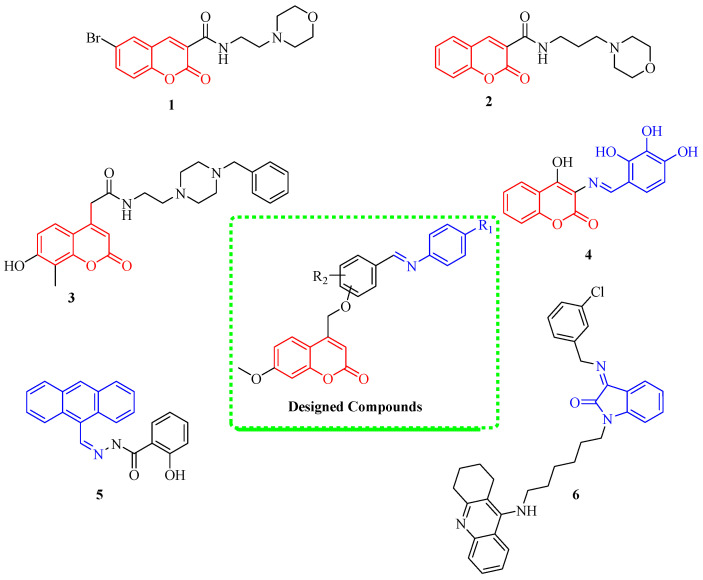
Some reported anticholinesterase agents and designed compounds in this work.

Alzheimer’s disease (AD) is the main cause of dementia and one of the most prevalent neurodegenerative diseases that impact people around the world. It is characterized by progressive memory loss, severe behavioral abnormalities, and cognitive impairments [1]. By 2050, the number of AD patients will have tripled from the present level of more than 50 million worldwide [2]. The cholinergic hypothesis is one of the most significant hypotheses, despite the fact that the etiology of AD has not yet been fully explained. In the brains of AD patients, it was found that the level of the neuromodulator acetylcholine (ACh) was abnormal. Studies have shown that acetylcholine levels rise when acetylcholinesterase (AChE) is inhibited, thereby enhancing memory and cognitive function in patients [3]. Therefore, a major treatment strategy for AD involves inhibiting the acetylcholinesterase (AChE) enzyme, which catalyzes the hydrolysis of ACh neurotransmitters. Currently, drugs such as donepezil, rivastigmine, and galantamine, which belong to the class of acetylcholinesterase inhibitors (AChEIs), are approved and administered as therapeutic options for the management of AD [4]. However, while these medications can provide temporary improvements in memory and cognitive function, they are unable to prevent or reverse the progression of the disease. Given the limitations of current treatments and the urgent need to address both symptomatic relief and disease progression, researchers are continually seeking and developing potential agents that can effectively treat AD and potentially modify its course.Heterocyclic-based compounds have been reported as active inhibitors against the acetylcholinesterase enzyme (AChE). Medicinal chemists have developed and used a variety of coumarin cores to design novel treatments with a wide spectrum of pharmacological properties [4]. Mohammadi-Khanaposhtani et al. have investigated the inhibition of AChE with a series of coumarin-3-carboxamide-*N*-morpholine derivatives. The obtained IC_50_ values ranged in micromolar levels (6.21–26.4 µM). Compounds **1** and **2** were found to be the most active among the series [5]. Kara et al. reported the synthesis and AChE inhibition activity of a series of coumarin-based compounds. Among the series, derivative **3,** with an IC_50_ value of 0.04 µM, displayed higher activity [6]. On the other hand, Schiff bases represent an important group of organic compounds with various biological activities [7,8,9,10,11]. The study of novel biologically significant Schiff bases has been drawing the attention of chemists and pharmacists [12,13,14,15]. Wang et al. have designed and synthesized a series of Schiff bases containing coumarin cores. The synthesized compounds were assessed for their anti-Alzheimer activity. The obtained results showed that derivative **4** showed higher inhibitory activities with IC_50_ values of 0.673 and 0.711 µM for human monoamine oxidasehMAO-A and hMAO-B, respectively [16]. Compound **5** showed promising inhibitory activity against AChE with an IC_50_ value of 4.12 µM [17]. In 2019, 16 hybrids of acrine-isatin Schiff bases were designed and synthesized by Riazimontazer and co-workers. The designed compounds were tested as potential anti-Alzheimer agents. Compound **6,** with an IC_50_ value of 0.42 nM, was the most potent (IC_50_ = 38.72 nM). Interestingly, chloro-substituent compounds displayed stronger inhibitory activity than those with nitro and methoxy substituents [18]. Figure 1 shows that some coumarin and Schiff base-containing compounds were reported as AChE inhibitors.

Our research group has reported chalcone-based coumarin scaffolds and psoralen derivatives as potential acetylcholinesterase inhibitors [19,20] in our ongoing search for a lead compound in drug discovery, as illustrated in Figure 1. This concept led us to design and synthesize a novel series of coumarin-Schiff base hybrids. This may contribute to discovering more effective inhibitors against AChE. Molecular modeling analyses of the synthesized compounds were performed to clarify their interaction modes and stabilities with the amino acid residues of AChE and explore their pharmacological effects. Finally, comprehensive reports were provided on the density functional theory (DFT) calculations, in silico assessments of local and global reactivity, and drug-likeness predictions using ADMET for the designed hybrids.

## 2. Results and Discussion

### 2.1. Chemistry

**Scheme 1:**
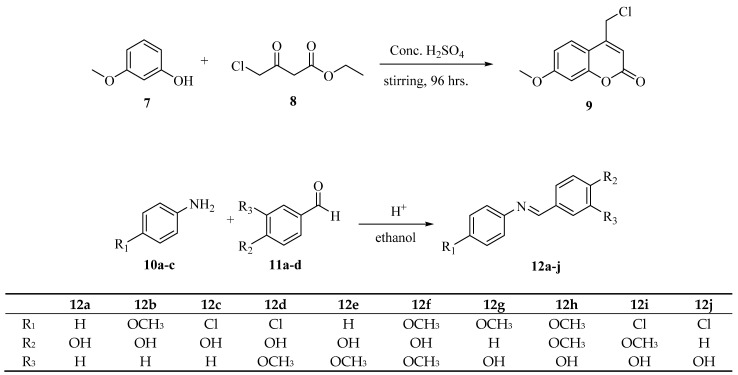
Synthesis of coumarin **9** and Schiff bases (**12a-j**).

The Pechmann condensation was performed between 3-methoxyphenol **7** and ethyl 4-chloroacetoacetate **8** in the presence of concentrated sulfuric acid. This reaction resulted in the formation of 4-(chloromethyl)-7-methoxy-2H-chromen-2-one 9 as a white powder with a yield of 94.0% (Scheme 1). Schiff bases (**12a-j**) were synthesized via an acid-catalyzed condensation reaction between commercially available hydroxybenzaldehydes (**11a-d**) and various aniline substituents (**10a-d**) in ethanol for 24 h. These compounds were synthesized in moderate to good yields (56.7–89.6%).

**Scheme 2:**
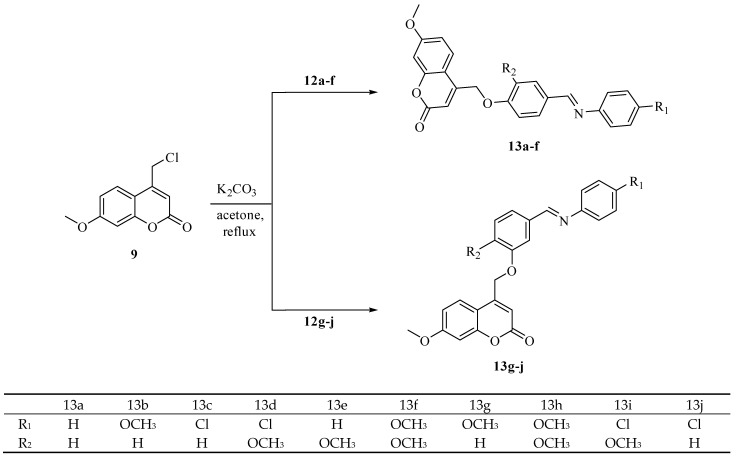
The Synthesis of Coumarin-Schiff bases Hybrids (**13a-j**).

The formation of Schiff bases (**12a-j**) is illustrated in Scheme 1. The presence of the expected CH=N group in these imines is strong evidence for the formation of target compounds and could be confirmed by IR and ^1^H NMR. The IR spectra of compounds (**12a-j**) showed characteristic C=N bands from (1603–1627 cm^−1^). Moreover, their ^1^H NMR spectra exhibited singlet signals in the range (δ 8.44–8.54). Thus, they are in agreement with previous studies [21,22,23]. Finally, the newly synthesized 4-(chloromethyl)-7-methoxy-2H-chromen-2-one **9** and Schiff bases (**12a-j**) were combined to form coumarin-imine hybrids (**13a-j**) following the procedure outlined in Scheme 2.

### 2.2. Anti-Acetylcholinesterase of Coumarin-Imine Hybrids (***13a-j***)

**Figure 2:**
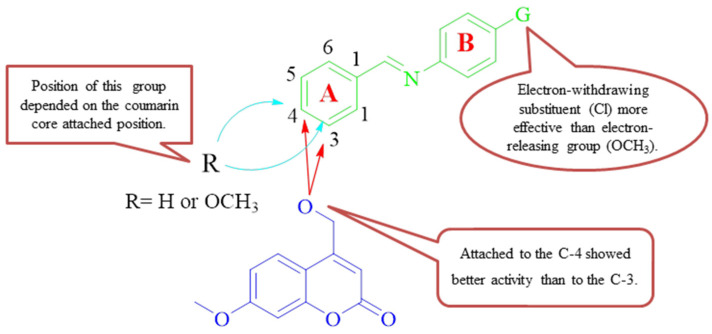
Summary for SAR study of the target hybrids (**13a-j**).

The in vitro activity of the coumarin-imine series (**13a-j**) was mentioned in Table 1. The study found that all hybrids (except for compound **13h**) had excellent inhibition activity. It was observed that the compounds with chloro substituent, which reduced electron density on the aromatic ring [24], exhibited better biological activity in comparison to those without a substituent or with a methoxy group at C-4 of the ring-B (Figure 2). As for chloro-hybrids, **13c** and **13d** showed activity at 0.232 and 0.190 μM, respectively. These two compounds were more potent than galantamine by ~5-fold. However, the IC_50_ value calculated for coumarin **13h** (1.175 μM) was lower than the positive control.

Furthermore, the introduction of a methoxy group on the ring-B, as in compounds **13b**, **13f**, **13g,** and **13h,** decreased anti-AChE activity, whereas the inhibitory activity against the enzyme was increased by the incorporation of a chloro group, as in compounds **13c**, **13d**, **13i** and **13j**. That is worth mentioning; the connection of the imine moiety to the coumarin core at 4-O was preferred. The structure-activity relationship (SAR) study of hybrids (**13a-j**) was summarized as follows (Figure 2): (1) The anti-AChE activity of the target compounds, which substituent group on imine ring-B was the electron-withdrawing group was better than that of the electron-donating group (-Cl *>* H *>* OCH_3_). (2) The bioassay outcome pointed out that the presence of chloro on C-4 of ring-B together with methoxy substituents on phenyl ring-A at the third position of the imine scaffold enhanced the AChE inhibition. (3) The coumarin core attached to the imine moiety via the O atom at C-4 was more active than those attached at C-3.

### 2.3. Molecular Docking

**Figure 3:**
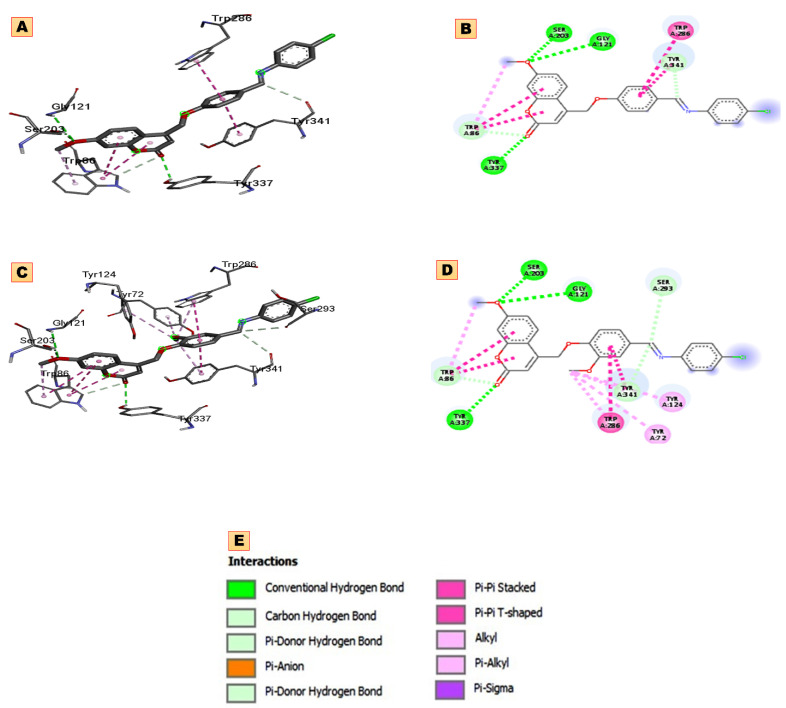
The proposed binding modes of synthesized hybrids (**13c** and **13d**) docked in the active site of acetylcholinesterase (4EY7) protein; (**A**) 3D ligand interactions of **13c**, with the target receptor, (**B**) 2D ligand interactions of **13c**, with the target receptor, (**C**) 3D ligand interactions of **13d**, with the target receptor, (**D**) 2D ligand interactions of **13d**, with the target receptor, and (**E**) Representative keys for the type of interactions.

The proposed molecular docking scores and binding mode with representation keys for the type of interaction docking in the active site of the acetylcholinesterase (4EY7) protein of newly synthesized hybrids (**13a-j**) studied in this work are presented in Table 2 and Figure S1. Upon careful inspection of these results, derivatives (**13a-j**) displayed binding affinity values ranging from −10.60 to −13.2 kcal/mol, which was the highest as compared to the positive compound, galantamine (−9.60 kcal/mol). As shown in Figure S1, the coumarin moiety, located in the catalytic anionic site (CAS) of the receptor, established hydrogen bonds (HBs) between carbonyl oxygen and amino acid residues. Compounds **13a**, **13c,** and **13d** formed HBs with GLY121 and SER203, but in cases **13c** and **13d** (Figure 3), another HBs was observed with TYR337, while hybrid **13a** formed HBs with amino acid residues of GLY122 and PHE295 with affinity energies of −11.9, −13.2, and −13.2 kcal/mol, respectively. Hybrids **13b** and **13e** formed another HBs with GLY122, but **13b** formed one HBs with SER203 while **13e** formed with TYR13, and their affinity energies values were −11.7 and −13.1 kcal/mol, respectively.

Compounds **13f**, **13h**, **13i,** and **13j** formed HBs with the TYR132, SER203, TYR132, and TYR337 amino acid residues, respectively, and the corresponding binding energies were −12.7, −10.6, −11.7, and −12.3 kcal/mol, respectively, with more than one HBs for compound **13j** with TYR124 amino acid residues (Table 2). In the case of hybrids **13c** and **13d**, the aryl ring of the coumarin core formed two π-π T-shaped interactions with residues of TRP86. Additionally, another aryl ring was established through two π-π stacking interactions with TRP286 amino acid residues. The CH_3_ group of coumarin interacted with residues of TYR74, TYR124, TRP86, and TRP286 through π-alkyl interactions. Careful inspection of the binding site pattern and binding energy between the AChE enzyme and the studied hybrids (**13a-j**) indicated that both hybrids (**13c** and **13d**) could be good candidates for the treatment of AChE, which is correlated with experimental results. We will be focusing on both (**13c** and **13d**) in our next discussion of stability complexes by molecular dynamics simulation.

### 2.4. Structural Activity Relationships (SARs)

In this study, the physicochemical properties such as molar volume (*V*), hydration energy (*HE*), molar refractivity (*MR*), surface area grid (*SAG*), and polarizability (*Pol*) for hybrids (**13a-j**) were calculated (Table 3) and discussed using HyperChem (v8.0.7). The molecular polarizability (*Pol*) characteristics of a compound are determined based on how efficiently its electronic system controls itself in response to the presence of an external electric field of light. The importance of molecular polarizability is that it plays a crucial role in simulating a variety of compound characteristics and bioactivities [25]. Molecule volume, which controls things like blood-brain barrier permeability and intestinal absorption, is the main factor that influences molecular polarizability. Thus, molecular volume must be used in QSAR investigations to simulate molecular characteristics and bioactivities. A further SAR parameter is molar refractivity (MR), a steric characteristic that is dependent on the spatial arrangement of the phenyl ring in the compounds under evaluation. The spatial arrangement is significant because it is crucial to understanding how drug molecules interact with biological receptors. The London dispersive force, which is greatly involved in the interaction between drug molecules and receptors, is another factor that influences molar refractivity in addition to its dependence on molecular volume.

According to the findings in Table 3, the size (volume) and molecular weight of proposed hybrids are often proportional to polarizability data, molecular refractivity, and surface area grid, such as hybrid **13f,** which has the highest volume value (1263.19 Å^3^), refractivity (123.14 Å^3^), maximum polarizability value (48.94 Å^3^), surface area grid (750.75 Å^3^), and the highest molecular weight (MW) (445.47 amu). On the other hand, compound **13a,** which has lower values in all five descriptors (molecular volume, polarizability, refractivity, surface area grid, and MW) are (1111.03 Å^3^, 44.0 Å^3^, 110.22 Å^3^, 668.68 Å^2^, and 445.47 amu), respectively. From Table 3, other compound gradients decrease in order as **13h** > **13d** > **13i** > **13b** > **13e** > **13g** > **13c** > **13j** is the same pattern in all hybrids.

The obtained results in Table 3 exhibit an increase in the values of hydrophobicity, causing a decline in hydration energy. The hydration energy determines the various molecular conformations’ stability in aqueous solutions [26,27]. The change in the hydration energy value is affected by the increase or decrease in the number of hydrogen bonds (acceptors and donors). Table 3 illustrates the absolute values of hydration energy ordered as **13f** < **13h** < **13d** < **13i** < **13b** < **13e** < **13g < 13c < 13j < 13a** with values of (−12.78, −12.58, −10.84, −10.62, −12.67, −11.21, −12.52, −10.72, −10.57, and −11.09 kcal/mol)*,* respectively, and characterized by hydrogen bonds (acceptors and donors).

Lipophilicity is a major determinant of many ADME properties. *Log P* expresses the portioning of the drug molecules between the aqueous medium outside the cell membrane and the lipid nature of the cell membrane. This means that compounds with a lower *Log P* are more polar and have poorer lipid bilayer permeability, whereas hybrids with a higher *Log P* are more nonpolar and poorly soluble in water [28]. For that reason, all compounds except compounds **13c** and **13j** have good aqueous solubility. Furthermore, *Log P* values of compounds **13f** = **13h** < **13d** = **13i** < **13b** = **13e** = **13g** < **13a** are in the field of optimal values (0 < *Log P* < 5) [29]. It can be concluded that these hybrids have optimal biological activity and good oral bioavailability. Hybrids **13c** and **13j** need a drug delivery carrier to deposit them on the surface of a suitable nanomaterial with specific properties to enhance oral bioavailability.

### 2.5. Molecular Dynamics Simulation and System Stability

**Figure 4:**
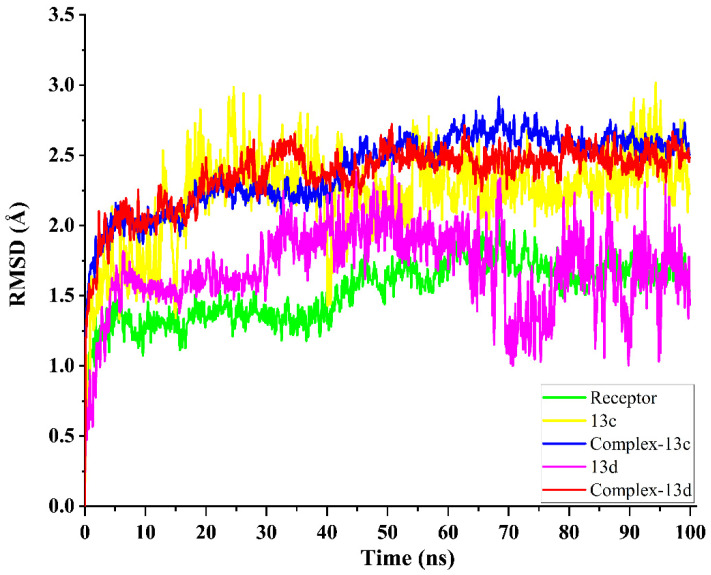
The root-mean-square deviation (RMSD) of the solvated receptor backbone, hybrids (**13c** and **13d**), and the receptor.

The conformational stability of the complex interaction is influenced by the molecular interactions and the solvent conditions around the receptor. As the initial structure, the best-docked pose of the most active compounds (**13c** and **13d**) with the highest binding affinity was chosen. Moreover, MD was performed to explore the interaction modes and stability of these compounds [30,31]. For this reason, a long-range MD simulation of 100 ns was investigated to study structural stability and conformational stability as well as the dynamics of protein-ligand complexes. Herein, the root-mean-square deviation (RMSD) throughout the 100 ns simulations was used to determine the stability of the systems. An RMSD value lower than 3.0 Å was the most acceptable; it shows that the system is the most stable [20]. For all frames of the AChE protein, ligands (**13c** or **13d**), and ligand-protein complex systems, as presented in Figure 4, the average RMSD values were 1.530, 1.691, and 2.364 Å in the **13d** complex, whereas in the **13c** complex, they were 1.631, 2.229 and 2.396 Å, respectively. The standard deviation of the average RMSD values were 0.199, 0.296, and 0.218 Å, in the **13d** complex, whereas in the **13c** complex, they were 0.208, 0.328, and 0.262 Å, respectively. These results revealed that, compared to the **13c**-AChE complex, the other complex (**13d**-AChE) established a significantly more stable conformation. During MD simulation production, examining amino acid residue behavior and its interaction with the compound necessitates assessing receptor structural flexibility upon compound binding [20].

**Figure 5:**
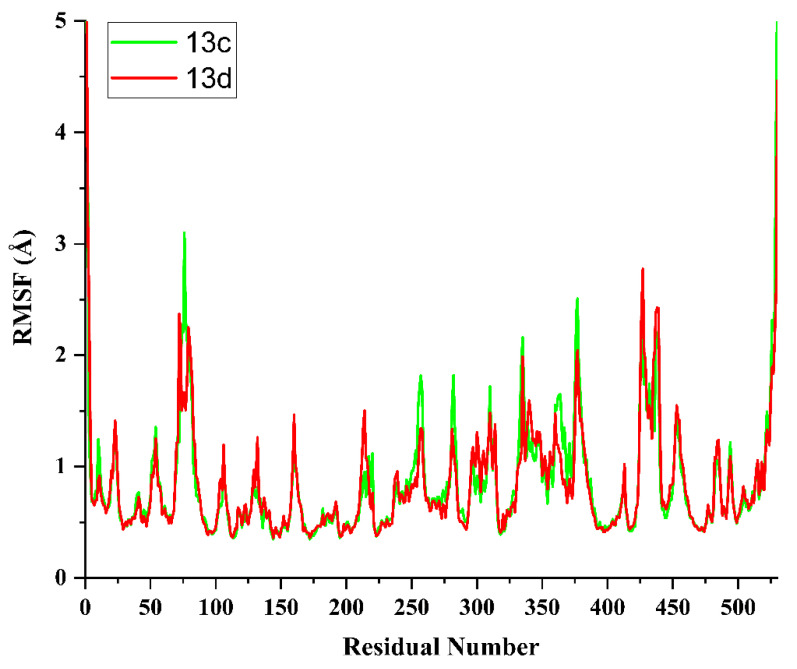
The root-mean-square fluctuation (RMSF) of solvated receptor backbone and hybrids (**13c** and **13d**) with a receptor.

**Figure 6:**
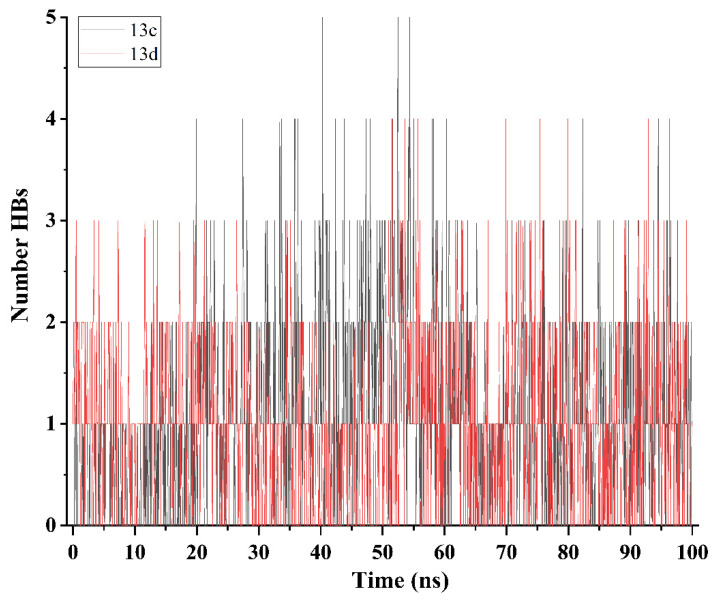
The number of average hydrogen bonding interactions between receptor-hybrids (**13c** and **13d**).

**Figure 7:**
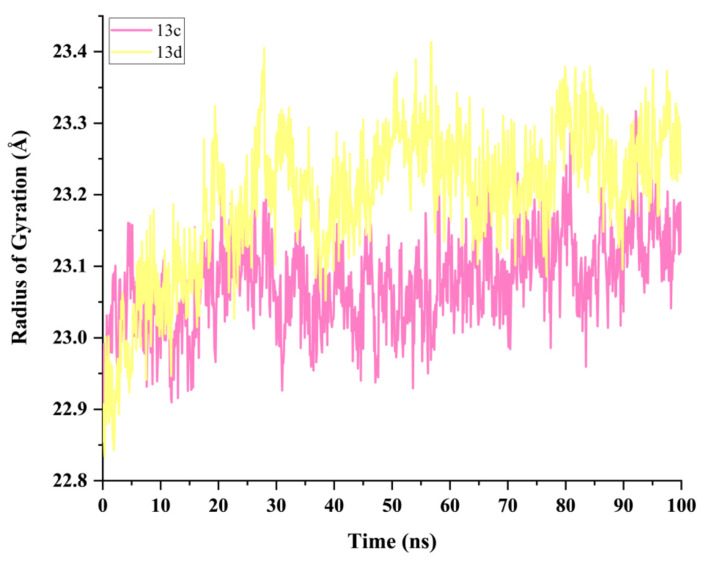
Radius of gyration (Rg) of receptor-ligands (**13c** and **13d**).

The residue changes were evaluated through the root-mean-square fluctuation (RMSF) method to study the effect of ligand binding to the relevant targets throughout the simulations (100 ns). The calculated average RMSF value for the **13c**-AChE complex was 0.85 Å, whereas its value for the **13d**-AChE complex was 0.83 Å to protein systems. Figure 5 illustrates the overall amino acid residue fluctuations of both complex systems. These findings indicate that the inhibition of the **13d**-AChE complex system is lower than that of the complex (**13c**-AChE) system, which will reflect well on the complex stability. Figure 6 depicts the number of hydrogen bond interactions between hybrids (**13c** and **13d**) and the target protein (AChE) with an angle cut of 10 degrees and an rcut of 3.0 Å, plotted against simulation time (100 ns). The average number of hydrogen bonds per timeframe was calculated to be 1.09 for **13c**-AChE and 1.005 for **13d**-AChE. It could be observed that the interactions dramatically increased the number of hydrogen bonds per trajectory analysis from 1 to 5 HBs. Therefore, the obtained results show that the system **13d** complex acquired a relatively more stable conformation than the other system, the **13c** complex. The radius of gyration (Rg) provides evidence for both simulation stability and protein structural compactness. R_g_ values for the studied complexes were 23.077 and 23.198 Å, respectively, as shown in Figure 7. R_g_ of **13c**-AChE showed a more rigid structure than **13d**-AChE.

**Figure 8:**
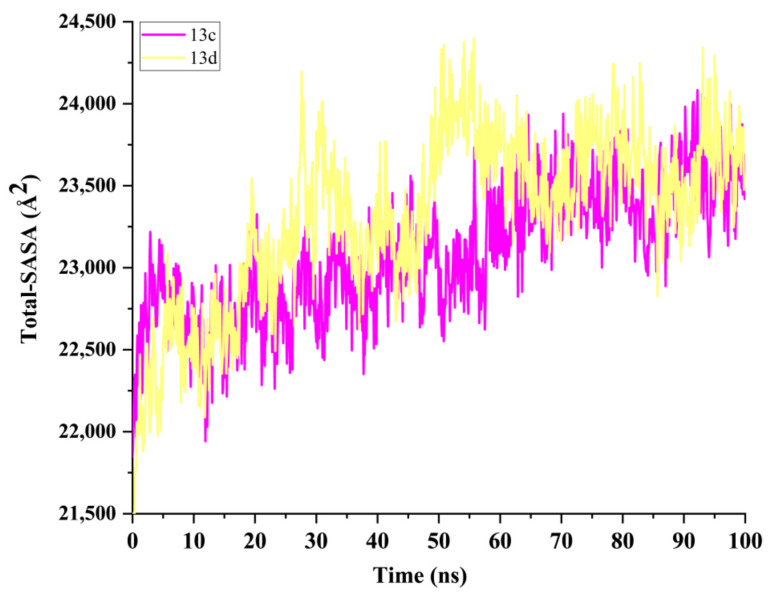
Solvent accessible surface area (SASA) analysis for receptor-hybrids (**13c** and **13d**).

**Figure 9:**
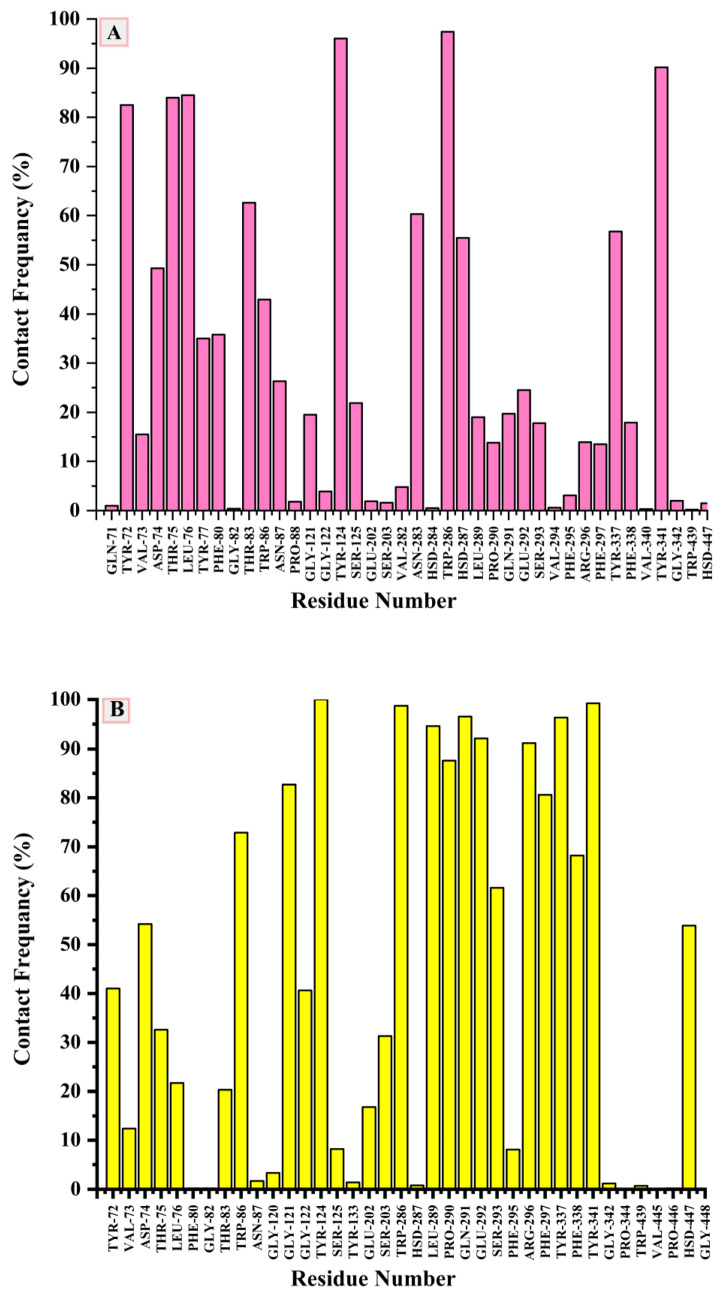
Contact frequency percentage analysis for receptor-hybrids; (**A**) **13c,** and (**B**) **13d**.

The MD simulation in ligand-bound conditions was utilized to determine the solvent-accessible surface area (SASA) of the receptor. As shown in Figure 8, when the ligand bound to the target receptor, the SASA values changed. The analysis shows that the **13d**-protein complex is more stable upon ligand binding than the **13c**-protein complex in regards to protein folding states and stability. To estimate the binding between the ligands **13c** or **13d** and AChE complexes, the contactFreq.tcl module on VMD and a cutoff of 4 Å were used to perform a contact frequency (CF) study, as shown in Figure 9a,b. In the simulation **13c** complex case study, the following amino acid residues exhibited higher CF values: TYR72, THR75, LEU76, TYR124, TRP286, and TYR341. Whereas in the **13d** complex, there were good contact surfaces with the protein pocket. Through this study, the following residues displayed higher CF values: TRP86, GLY121, TYR124, TRP286, LEU289, PRO290, GLN291, GLU292, ARG296, PHE297, TYR337, PHE338, and TYR341.

### 2.6. Binding Free Energy by MM/GBSA Methods

The molecular mechanics energy approach (MM/GBSA), which combines surface area continuum solvation and generalized Born, is a commonly used approach for calculating the free binding energies of small molecules to biological macromolecules. This approach might be more reliable compared to docking scores [32]. Therefore, to validate the docking scores that were anticipated by molecular docking studies for hybrids **13c** and **13d** against the AChE receptor, the binding free energy of the simulated ligand-protein was determined. The binding free energy of the simulated complex was computed to revalidate the inhibitor affinity predicted by docking simulation studies for the hybrids **13c** and **13d** with the AChE receptor. The MolAICal tool was used to take snapshots of the system trajectories being investigated in order to determine the binding free energy [33]. All the reported computed energy solvation components are presented in Table 4. Most negative values demonstrate favorable interactions. The binding free energy of both complexes **13c** and **13d** was calculated to be −23.645 and −36.042 kcal/mol, respectively. A close inspection of the individual energy contributions displays that the van der Waal energy of both complexes was found to be **13c**-AChE with −44.913 kcal/mol, which had less binding affinity. Whereas **13d**-AChE (−51.081 kcal/mol) exhibited strong binding affinity. The electrostatic energy for both complexes has considerable and moderate values. The binding free energy shows that the **13d**-AChE complex was found to have more stability than the **13d**-AChE complex.

### 2.7. Density Functional Theory (DFT)

#### 2.7.1. Molecule Orbital Calculations

The optimized geometrical parameters (dihedral angles, bond angles, and bond lengths), natural charges, natural population of the nucleus of proposed derivatives, the energetics of the ground state, molecular electrostatic potential maps, and reactivity descriptors of synthesized hybrids were calculated and analyzed utilizing the spectroscopic data and elemental analysis.

##### Ground State Geometric Parameters (S281–S299)

The optimized geometry, dihedral angles, bond angles, bond lengths, vector of the dipole moment, and numbering system of coumarin hybrids (**13a-j**) are presented in Table S1 and Figure S2. In the present work, the gas-phase wB97XD/6-311++G(d,p) has been compared to the available crystal data of 7-acetoxy-coumarin (ref. CCDC 1113091) [34] to evaluate the geometrical parameters. The mean absolute errors (MAE) that were calculated for the bond lengths and angles of the coumarin nucleus are given in Table S2. Careful inspection shows that MAEs range from 0.001 to 0.051 Å in bond length, from 0.014 to 0.562 degrees in bond angles, and from 0.004 to 1.354 degrees in dihedral angles in long-range corrected hybrid functionals (wB97XD), which give complete reducibility in predicting bond lengths and angles with experimental results with respect to experimental results and computational time and power uses. Therefore, the wB97XD/6-311++G (d,p) level of theory was selected for geometry optimizations and all calculations. The majority of the calculated bond lengths show underestimated values with percentages ranging from 0.25 to 1.1% in O1-O11 and an overestimation with values ranging from 1.1 to 5.1% in C8-C17. Generally, there is no major change. Inspection of the values of the dihedral angles compiled in Table S1 shows that almost all molecules are planar except the N36-phenyl moiety, which is out of plane and perpendicular in all the studied compounds (**13a-j**) with dihedral angle values of −41.8 to −47.6 degrees. The bond angle values calculated are shown in Table S1. Results vary from 109.7 to 125.0 degrees, which nicely compares to a regular SP^2^ hybridization geometry.

##### Natural Charges and Natural Population

Natural charge analysis performed on the electronic structures of synthesized compounds (**13a-j**) clearly describes the distribution of electrons in various subshells of their atomic orbitals. The charge analysis carried out for all compounds using wB97XD/6-311++G(d,p) level of calculation is presented in Table 5. In Table 5, the most electronegative charges for hybrids (**13a-j**) are accumulated on O11, O24, O16, O1, N36, and C7, respectively, from −0.563 e to −0.313 e. According to an electrostatic point of view, these electronegative atoms tend to have active sites for electrons. However, the most electropositive atoms, such as C2, C10, C8, and C25, from +0.774e to +0.271e, respectively, tend to accept electrons at their active sites. Going from **13a** to **13j** is a minor change in natural charge with the same pattern of electrostatic mapping with the order. An in-depth study of the natural charge pattern of hybrids is extremely beneficial for gaining insight into the crucial interactions between title hybrids and biological receptors of AChE, which enhances the investigation of cytotoxicity activity.

##### Frontier Molecular Orbitals (FMOs) Analysis

**Figure 10:**
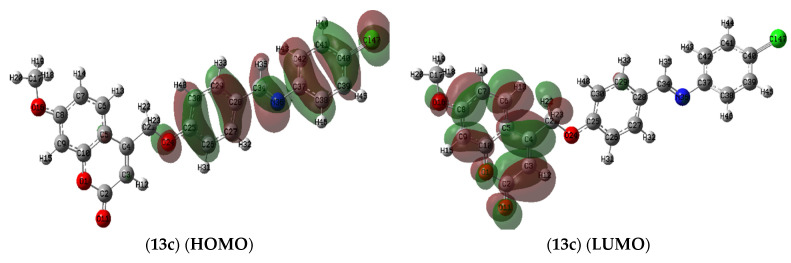

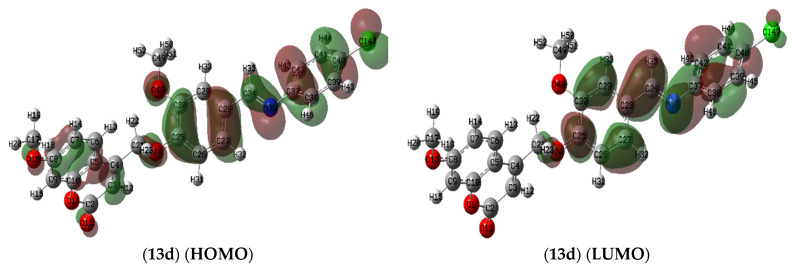
Frontier molecular orbitals of the compounds (**13c** and **13d**) utilizing wb97xd/6-311++g(d,p) level of calculation.

As presented in Table 6, among all synthesized hybrids, **13e** displayed higher stability and less reactivity with an energy gap value of 7.96 eVm, whereas hybrid **13b** (ΔE_gap_ = 7.38 eV) showed the lowest stability and highest reactivity [35,36,37,38]. The energy gaps of the rest of the hybrids were ordered as follows: **13j** < **13i** < **13d** < **13c** < **13a** < **13f** < **13h** < **13g**. Due to the significance of the parameters such as I (potential ionization) and A (electron affinity), their calculations enable us to determine the global reactivity descriptors. The I and A parameters are related to the one-electron orbital energies of the HOMO and LUMO. The obtained results (Table 6) exhibited that hybrids **13j** had the highest values of I (8.29 eV) and A (0.37 eV). The I value of the other hybrids are **13d** > **13c** > **13e** > **13i** > **13a > 13g > 13f > 13b > 13h.** Whereas in the case of A values, the order is as follows: **13d > 13c > 13a > 13b > 13i > 13g > 13e > 13f > 13h**. Then we can predict that derivatives **13j**, **13d,** and **13c** are the best candidates for interaction with other biological AChE receptors. All of the hybrids have almost similar HOMO and LUMO isodensity dispersion as depicted in Figure S3, except for **13c** and **13d** (Figure 10), which have slightly different dispersion on the coumarin rings and the (E)-*N*-benzylideneaniline moiety. The direction of the electronic charge transfer motion is represented by the dipole moment vector with the order norm vector; synthesized compounds are ordered as **13j > 13i > 13f > 13e > 13a > 13d > 13c > 13h > 13b > 13g.**

##### Global Reactivity Descriptors

The density functional theory (DFT) uses the chemical system’s electron density to explain several basic ideas about how chemicals react [39]. In chemistry, understanding the nature of chemical interactions and predicting the chemical reactivity of molecules, atoms, or ions are the two most challenging problems. Herein, we studied the reactivity of novel synthesized derivatives (**13a-j**). Table 7 shows the values of the important reactivity descriptors that help us figure out how reactive and stable hybrids (**13a-j**) are. Among all compounds, coumarin **13j,** with the highest value of η = 3.98 eV, is the chemically hardest compound, while coumarin **13b**, with the lowest value (η = 3.69 eV), is chemically soft and more reactive. The chemical hardness of other hybrids is ordered as **13j** > **13i** > **13d** > **13c** > **13a** > **13f** > **13h** > **13g**. A general idea of charge transfer in any molecule’s ground state can be obtained from the electronic chemical potential (V) value. In terms of chemical potential, hybrid **13h** has the greatest value (−3.8 eV), whereas hybrid **13j** has the lowest (−4.33 eV), and other coumarin hybrids are in the following order: **13f > 13b > 13g > 13e > 13i > 13a > 13c > 13d** (Table 7).

A thermodynamic parameter that is represented by the electrophilicity index (ω) estimates the energy changes that occur when a chemical system reaches saturation with the addition of more electrons. This is very beneficial in determining a system’s chemical reactivity. As shown in Table 7, coumarin hybrid **13h** is nucleophilic in nature with the lowest electrophilicity index value equal to 1.9 eV, while hybrid **13j** is strongly electrophilic in nature (ω = 2.37 eV). The lowest electrophilicity index (ω) order of other coumarins is the same as electron affinity (**A**). The electronegativity (**X**) describes the tendency of an atom in a covalent bond to draw electrons towards it. From the obtained electronegativity of the synthesized hybrids, coumarin **13j** is the best electron acceptor with **X** = 4.33 eV, which had the highest electronegativity value as compared to the other hybrids. The electronegativity (**X**) order of other coumarins shows the same behavior in both the electron affinity and electrophilicity indexes. In terms of global softness, hybrid **13b** displayed the highest reactivity and softness values (0.14 eV), whereas the other hybrids exhibited almost the same values of softness (0.126 to 0.133 eV) with order **13g > 13h > 13f > 13a > 13c > 13d > 13i > 13j > 13e**.

##### Local Reactivity Descriptor

To study the site selectivity and chemical reactivity of a molecule, the principles of local reactivity descriptors have been frequently applied [40,41]. The Fukui function is a local descriptor that can be used to study molecular site selectivity [42]. It is the first derivative of the electronic density ρ(r) in relation to the electron numbers (N) of a system at a constant external potential ν(r) [43], as represented in the following equation:

$$f\left(r\right)={\left(\frac{\partial \rho \left(r\right)}{\partial N}\right)}_{v\left(r\right)}=\frac{1}{2}\;{\left(\frac{\partial \mu }{\partial v\left(r\right)}\right)}_{v\left(r\right)}$$

Based on the changes in electrical density throughout a reaction process, we can calculate Fukui functions to identify the active sites. As shown in the following equation, for the three different environments of chemicals, the Fukui functions *f^+^* (r), *f^–^* (r), and *f^0^* (r) are determined [44,45,46]:

$$\begin{array}{l} f^{-}\left(r\right)={\;q}_{k}\left(N\right)-q_{k}\left(N\;-1\right)\;\approx {\;\rho }^{\mathrm{HOMO}}\left(r\right)\;\mathrm{for}\;\mathrm{electrophilic}\;\mathrm{attack}\\ f^{+}\left(r\right)={\;q}_{k}\left(N+1\right)-q_{k}\left(N\;\right)\approx \rho^{\mathrm{LUMO}}\left(r\right)\;\mathrm{for}\;\mathrm{nucleophilic}\;\mathrm{attack}\\ f^{0}\left(r\right)=\frac{1}{2}\left[q_{k}\left(N+1\right)-q_{k}\left(N\;-1\right)\right]\approx \frac{1}{2}\left[\rho^{\mathrm{HOMO}}\left(r\right)+\rho^{\mathrm{LUMO}}\left(r\right)\right]\;\mathrm{for}\;\mathrm{radical}\;\mathrm{attack} \end{array}$$

where $q_{k}\left(N\right)$, $q_{k}\left(N+1\right)$ and $q_{k}\left(N-1\right)$ are the atomic populations on the *k_th_* atom for the neutral molecule, anionic and cationic species, respectively. Tables S3 and S4 represent the descriptor values of coumarin hybrids (**13a-j**) computed at the wb97xd/6-311++G (d, p) level. In addition to knowing how an atomic site in a molecule could be electrophilic or nucleophilic, Labbe et al. [47] suggested an additional dual descriptor (Δf(r)) that is provided by the following equation:

$$\Delta f\left(r\right)=f^{+}\left(r\right)-f^{-}\left(r\right)$$

The obtained results indicate that the most electrophilic reactivity is on the imine moiety, which is mostly found on the atoms O24, N36, C37, C40, and C42, while the nucleophilic active site on the coumarin moiety is located on O1, C2, C3, C4, C6, C8, O11, and O16 localized. From Tables S3 and S4, when considering the dual descriptor Δf(r) for the nucleophilic and electrophilic attacks, as well as the philicity indices, the same result could be obtained. The high electronegativity of atoms N and O led to an electron density redistribution, in addition to the effect of -OCH_3_ insertion groups in R1 and R2 substitutions, that causes these characteristic changes. These findings are in agreement with the analysis of the natural population using calculated HOMO and LUMO. In 2004, Chattaraj et al. proposed the generalized philicity concept; with the help of corresponding condensed-to-atom Fukui function variations, they developed a local quantity known as philicity coupled with a site k in a molecule ($f_{k}^{\alpha }$), as given in the following equation [48].

$$\omega_{k}^{\alpha }=\omega f_{k}^{\alpha }$$

where α represents the local philic quantities, radical (α = 0), nucleophilic (α = +), and electrophilic (α = −) attacks. According to the aforementioned equation, the most electrophilic property has the highest value of $\omega_{k}^{\alpha }$. Moreover, different local softness was proposed by Lee et al. to define the reactivity of a molecule [49] as in the following Equation.

$$s_{k}^{\alpha }=sf_{k}^{\alpha }$$

In the aforementioned equation, α is represented by local softness quantities for nucleophilic (α = +), electrophilic (α = −), and radical attacks (α = 0). In order to complete the picture, the software package Multiwfn (v. 3.7) determined the local electrophilicity and nucleophilicity index, condensed local softness, and relative electrophilicity/nucleophilicity for each atom in the compounds from a CDFT point of view [50]. A close inspection would show that all the compounds had the donating and the back-donating processes at the center of their active sites, in agreement with the Fukui functions and also with the frontier orbital, as shown in the results obtained, which are boldly given in Tables S5 and S6. These findings showed the studied compounds to have several active sites, making them able to interact with the surface of pocket proteins via donating electrons. Lastly, the aforementioned local descriptors show that the experimental data in this study are in agreement with the theoretical variation of the compound’s efficiency.

##### Molecular Electrostatic Potential (MEP)

In several fields of chemistry, the electrostatic potential (ESP) on molecular surfaces has become one of the most effective tools for identifying, analyzing, and understanding trends [51,52]. It is related to electronic density, which is an excellent descriptor for describing the charge distributions on a molecule, identifying regions that are differently charged, and identifying the sites where hydrogen-bonding interactions, electrophilic properties, and nucleophilic properties are most likely to take place [53]. Electrostatic potentials (ESPs) are essential for predicting and understanding intermolecular interactions [52]. The crucial interactions between the synthesized hybrids and biological targets must be better understood with the help of an in-depth analysis of their ESPs. The ESP, as denoted by *V*(*r*) (in a.u.) at a given point r (x,y,z) in the molecule’s vicinity, is a calculation of the electrostatic energy that a positive unit test charge would experience at that point. Negative and positive ESPs corresponded to attractive and repulsion interactions, respectively. The following equation defines the ESP as the interaction energy between a proton at *r* and the electrical charge produced by the electrons and nuclei.

$$V\left(r\right)=\overset{nuclei}{\underset{A}{\sum }}\frac{Z_{A}}{\left|R_{A}-r\right|}-\int \frac{\rho \left(r^{\prime }\right)}{\left|r-r^{\prime }\right|}dr^{\prime }$$

where *Z_A_* is the charge, *R_A_* is the position of nucleus *A*, and ρ (*r’*) is the electron density at position *r’*.

**Figure 11:**
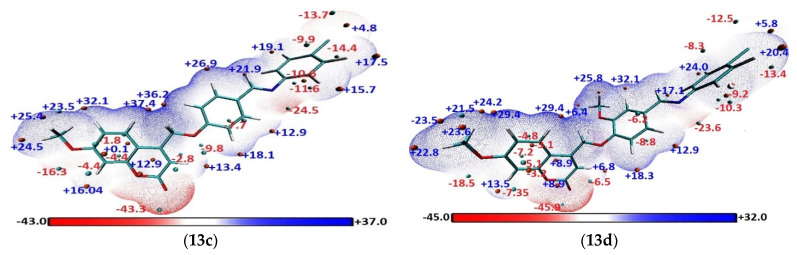
Molecular electrostatic potential (MEP) surfaces for the optimized geometry of hybrids (**13c** and **13d**) based on wb97xd/6-311++g(d,p).

The electrostatic potentials-mapped surfaces of the hybrids (**13a-j**) are presented in Figure S4. The overall van der Waals surface can be divided into several fragments by the quantitative molecular surface analysis module of the Multiwfn package, and this capability enables us to analyze the features of the ESP distribution. For hybrid **13a**, the surface displays a high negative value of ESP at the O11, O16, O24, and N36 (−43.97, −16.9, −11.0, and −28.01 kcal/mol), respectively, with the positive charge being distributed among various active sites. As for the potential of various derivatives (**13b-j**) to redistribute electrons, the global minima of ESPs around O11, O16, O24, and N36 centers increase, reaching minimum values in case **13f** with sequence (−46.9, −18.5, −22.0, and −27.9 kcal/mol) due to the electron-donating OCH_3_ group but in case **13c** and **13d** (Figure 11), Cl derivatives show that the negative decrease due to electron drawing behavior as sequence (−43.3, −16.3, −9.8 and −24.5 kcal/mol) and (−45.9, −18.5, −6.5 and −23.5 kcal/mol), respectively. The global maxima of ESPs on the derivatives (**13b-j**) surfaces are located on the carbon with the proton of these derivatives, which vary from +36.2, +35.6, +37.4, +29.4, +28.3, +27.6, +34.1, +26.8, +28.7 and +35.9 kcal/mol for hybrids (**13a-j**), respectively. This indicates that electrostatic or hydrogen bonding will be the main interaction between hybrids and their target receptors. The ability to generate hydrogen bond interaction and intramolecular charge transfer (ICT) is confirmed by a careful examination of these ESP values, indicating that they can act as therapeutics. These values show the same findings from the analyses of the NBO population and local reactivity descriptors reported in the previous section.

## 3. Experimental

### 3.1. General Procedures

Reagents and solvents used in the research were commercially available. The reagents 3-Methoxyphenol, ethyl 4-chloroacetoacetate, 4-hydroxy-3-methoxybenzaldehyde, 3-hydroxy-4-methoxybenzaldehyde, 4-chloroaniline, and 4-methoxyaniline were purchased from Sigma-Aldrich. The other materials were purchased from Acros Organics, such as aniline, glacial acetic acid, anhydrous potassium carbonate, acetone, chloroform, dichloromethane, ethyl acetate, methanol, ethanol, and *n*-hexane. Analytical grade (AR) solvents such as ethanol absolute AR (99.9%) were purchased from Fisher Scientific. The purity of these chemicals was 90–99.9%, and they were used without further purification. Thin-layer chromatography (TLC) was used to detect compounds present in the products. The TLC plates used were the thin aluminum plates from Merck, pre-coated with silica gel F_254_ with 0.2 mm thickness. The spots were visualized under UV light at 254 nm or 365 nm. Melting points (uncorrected) were determined using a Barnstead Electrothermal 9100 melting point. The infrared IR spectra were recorded using a Perkin Elmer FTIR spectrometer. Samples were prepared as KBr discs. The ^1^H NMR (400 MHz) and ^13^C NMR (100 MHz) were recorded on a Bruker Avance II 400 MHz NMR Spectrometer using dimethyl sulfoxide (DMSO-d_6_, Sigma-Aldrich, 99.9%) as the solvent. Chemical shift values were given in δ (ppm) scales. The HRMS were recorded on an Agilent Technologies 6545 Q-TOF LC/MS.

#### 3.1.1. Synthesis of 4-(Chloromethyl)-7-methoxy-2*H*-chromen-2-One (**9**)

3-Methoxyphenol (30 mmol) was added to a cooled aqueous solution of 70% H_2_SO_4_ (60 mL) and followed by adding ethyl 4-chloroacetoacetate (35 mmol). The solution was stirred in an ice-bath for a period of 9 h and continuously stirred for 96 h. The reaction mixture was poured into cold water. The precipitate was filtered off, washed several times with cold water, dried, and recrystallized from ethanol to afford 4-(chloromethyl)-7-methoxy-2*H*-chromen-2-one (**9**) as a white powder (6.34 g, 94%); R*_f_* = 0.51 (hexane: EtOAc = 3:2); m.p 197–198 °C (lit. 199–200 °C [54]). IR (KBr) (υ _max_/cm^−1^): 3072 (C-H *sp^2^*), 2942 (C-H *sp^3^*), 1727 (C=O), 1615 (C=C olefinic), 1560–1499 (C=C aromatic), 1058 (C-O). ^1^H NMR (400 MHz, DMSO-d_6_): δ 3.87 (3H, s, OCH_3_), 5.00 (2H, s, CH_2_Cl), 6.50 (1H, s, H-3), 7.00 (1H, dd, *J* = 2.4 and 8.8 Hz, H-6), 7.05 (1H, d, *J* = 2.4 Hz, H-8), 7.76 (1H, d, *J* = 8.8 Hz, H-5).

#### 3.1.2. General Procedure of Synthesis of Schiff bases (**12a-j**)

Glacial acetic acid (1 mL) was added dropwise into a solution of hydroxybenzaldehyde (10 mmol) and substituted aniline (10 mmol) in 95% EtOH (20 mL). The reaction mixture was refluxed for one hour at 80 °C and left to stir at room temperature overnight. After that, the mixture was poured into iced water (25 mL). The solvent was removed by vacuum filtration to form a solid. The solids were purified by crystallization from ethanol to afford the desired Schiff bases (**12a-j**).

##### (E)-4-((Phenylimino)methyl)phenol (**12a**)

Pale yellow powder; yield: (1.24 g, 63%); R*_f_* = 0.47 (hexane: EtOAc = 3:2); m.p 193–194 °C (lit. 195–196 °C [55]). IR (KBr) (υ _max_/cm^−1^): 3438 (O-H), 1603 (C=N), 1577, and 1515 (C=C aromatic). ^1^H NMR (400 MHz, DMSO-d_6_): δ 6.88 (2H, d, *J* = 8.8 Hz, H-2 and H-6), 7.19 (3H, m, H-3′, H-4′ and H-5′), 7.37 (2H, t, *J* = 7.6 and 8.0 Hz, H-2′ and H-6′), 7.77 (2H, d, *J* = 8.8 Hz, H-3 and H-5), 8.45 (1H, s, CH=N), 10.15 (1H, s, OH).

##### (*E*)-4-(((4-Methoxyphenyl)imino)methyl)phenol (**12b**)

Pale yellow powder; yield: (1.48 g, 65%); R*_f_* = 0.51 (hexane: EtOAc = 3:2); m.p 208–209 °C (lit. 210–212 °C [56]). IR (KBr) (υ _max_/cm^−1^): 3432 (O-H), 1605 (C=N), 1575, and 1513 (C=C aromatic). ^1^H NMR (400 MHz, DMSO-d_6_): δ 3.76 (3H, s, OCH_3_), 6.86 (2H, d, *J* = 8.8 Hz, H-2 and H-6), 6.94 (2H, d, *J* = 8.8 Hz, H-3′ and H-5′), 7.21 (2H, d, *J* = 8.8 Hz, H-2′ and H-6′), 7.74 (2H, d, *J* = 8.8 Hz, H-3 and H-5), 8.45 (1H, s, CH=N), 10.15 (1H, s, OH).

##### (E)-4-(((4-Chlorophenyl)imino)methyl)phenol (**12c**)

Pale yellow powder; yield: (1.83 g, 79%); R*_f_* = 0.58 (hexane: EtOAc = 3:2); m.p 187–188 °C (lit. 184–185 °C [57]). IR (KBr) (υ _max_/cm^−1^): 3447 (O-H), 1600 (C=N), 1572, and 1515 (C=C aromatic). ^1^H NMR (400 MHz, DMSO-d_6_): δ 6.88 (2H, d, *J*= 8.4 Hz, H-2 and H-6), 7.22 (2H, d, *J*= 8.8 Hz, H-2′ and H-6′), 7.42 (2H, d, *J*= 8.8 Hz, H-3′ and H-5′), 7.77 (2H, d, *J*= 8.4 Hz, H-3 and H-5), 8.46 (1H, s, CH=N), 10.19 (1H, s, OH).

##### (*E*)-4-(((4-Chlorophenyl)imino)methyl)-2-methoxyphenol (**12d**) [58]

Yellow powder; yield: (2.35 g, 90%); R*_f_* = 0.54 (hexane: EtOAc = 3:2); m.p 193–194 °C. IR (KBr) (υ _max_/cm^−1^): 3394 (O-H), 1623 (C=N), 1584, and 1513 (C=C aromatic). ^1^H NMR (400 MHz, DMSO-d_6_): δ 3.84 (3H, s, OCH_3_), 6.89 (1H, d, *J* = 8.0 Hz, H-6), 7.22 (2H, d, *J* = 8.4 Hz, H-2′ and H-6′), 7.33 (1H, dd, *J* = 1.2 and 8.4 Hz, H-5), 7.42 (2H, d, *J* = 8.4 Hz, H-3′ and H-5′), 7.52 (1H, d, *J* = 1.6 Hz, H-3), 8.44 (1H, s, CH=N), 9.88 (1H, s, OH).

##### (*E*)-2-Methoxy-4-((phenylimino)methyl)phenol (**12e**)

Pale yellow powder; yield: (1.29 g, 57%); R*_f_* = 0.62 (hexane: EtOAc = 3:2); m.p 116–117 °C (lit. 110–114 °C [59]). IR (KBr) (υ _max_/cm^−1^): 3450 (O-H), 1621 (C=N), 1584, and 1515 (C=C aromatic). ^1^H NMR (400 MHz, DMSO-d_6_): δ 3.85 (3H, s, OCH_3_), 6.91 (1H, d, *J* = 8.0 Hz, H-6), 7.20 (3H, m, H-3′, H-4′ and H-5′), 7.33 (1H, dd, *J* = 1.2 and 8.0 Hz, H-5), 7.39 (2H, t, *J* = 7.6 and 8.0 Hz, H-2′ and H-6′), 7.54 (1H, d, *J* = 1.2 Hz, H-3), 8.44 (1H, s, CH=N), 9.80 (1H, s, OH).

##### (*E*)-2-Methoxy-4-(((4-methoxyphenyl)imino)methyl)phenol (**12f**)

Greenish-yellow powder; yield: (1.54 g, 60%); R*_f_* = 0.67 (hexane: EtOAc = 3:2); m.p 131–132 °C (lit. 133 °C [60]). IR (KBr) (υ _max_/cm^−1^): 3430 (O-H), 1622 (C=N), 1586, and 1506 (C=C aromatic). ^1^H NMR (400 MHz, DMSO-d_6_): δ 3.76 (3H, s, OCH_3_), 3.84 (3H, s, OCH_3_), 6.88 (1H, d, *J* = 8.0 Hz, H-6), 6.94 (2H, d, *J* = 8.8 Hz, H-3′ and H-5′), 7.22 (2H, d, *J* = 8.8 Hz, H-2′ and H-6′), 7.29 (1H, dd, *J* = 2.0 and 8.0 Hz, H-5), 7.51 (1H, d, *J* = 1.6 Hz, H-3), 8.46 (1H, s, CH=N), 9.71 (1H, s, OH).

##### (*E*)-3-(((4-Methoxyphenyl)imino)methyl)phenol (**12g**)

Greenish-yellow powder; yield: (1.49 g, 65%); R*_f_* = 0.38 (hexane: EtOAc = 3:2); m.p 123–125 °C (lit. 122.0 °C [61]). IR (KBr) (υ _max_/cm^−1^): 3443 (O-H), 1626 (C=N), 1592, and 1502 (C=C aromatic). ^1^H NMR (400 MHz, DMSO-d_6_): δ 3.77 (3H, s, OCH_3_), 6.90 (1H, d, *J* = 4.8 Hz, H-5), 6.96 (2H, d, *J* = 8.8 Hz, H-3′ and H-5′), 7.27 (4H, m, H-2′, H-4, H-6 and H-6′), 7.35 (1H, d, *J* = 2.4 Hz, H-2), 8.54 (1H, s, CH=N), 9.69 (1H, s, OH).

##### (*E*)-2-Methoxy-5-(((4-methoxyphenyl)imino)methyl)phenol (**12h**)

Beige color; yield: (1.46 g, 57%); R*_f_* = 0.55 (hexane: EtOAc = 3:2); m.p 255–257 °C (lit. 257–259 °C [62]). IR (KBr) (υ _max_/cm^−1^): 3422 (O-H), 1627 (C=N), 1610, and 1576 (C=C aromatic). ^1^H NMR (400 MHz, DMSO-d_6_): δ 3.76 (3H, s, OCH_3_), 3.83 (3H, s, OCH_3_), 6.94 (2H, d, *J* = 8.8 Hz, H-3′ and H-5′), 7.01 (1H, d, *J* = 8.0 Hz, H-3), 7.22 (2H, d, *J* = 8.8 Hz, H-2′ and H-6′), 7.26 (1H, dd, *J* = 2.0 and 8.4 Hz, H-4), 7.51 (1H, d, *J* = 2.0 Hz, H-6), 8.44 (1H, s, CH=N), 9.33 (1H, s, OH).

##### (*E*)-5-(((4-Chlorophenyl)imino)methyl)-2-methoxyphenol (**12i**) [63]

Yellow powder; yield: (1.86 g, 71%); R*_f_* = 0.39 (hexane: EtOAc = 3:2); m.p 179–181 °C. IR (KBr) (υ _max_/cm^−1^): 3432 (O-H), 1618 (C=N), 1599, and 1578 (C=C aromatic). ^1^H NMR (400 MHz, DMSO-d_6_): δ 3.84 (3H, s, OCH_3_), 7.03 (1H, d, *J* = 8.0 Hz, H-3), 7.23 (2H, d, *J* = 8.4 Hz, H-2′ and H-6′), 7.30 (1H, dd, *J* = 2.0 and 8.4 Hz, H-4), 7.42 (3H, m, H-3′, H-5′ and H-6), 8.44 (1H, s, CH=N), 9.40 (1H, s, OH).

##### (*E*)-3-(((4-Chlorophenyl)imino)methyl)phenol (**12j**)

Pale yellow powder; yield: (1.69 g, 73 %); R*_f_* = 0.41 (hexane: EtOAc = 3:2); m.p 134–136 °C (lit. 135 °C [61]). IR (KBr) (υ _max_/cm^−1^): 3384 (O-H), 1621 (C=N), 1598, and 1482 (C=C aromatic). ^1^H NMR (400 MHz, DMSO-d_6_): δ 6.94 (1H, d, *J* = 6.8 Hz, H-5), 7.27 (2H, d, *J* = 8.4 Hz, H-2′ and H-6′), 7.32 (3H, m, H-2, H-4 and H-6), 7.45 (2H, d, *J* = 8.4 Hz, H-3′ and H-5′), 8.53 (1H, s, CH=N), 9.78 (1H, s, OH).

#### 3.1.3. General Procedure of Synthesis of Coumarin-Schiff Base Hybrids (**13a-j**)

Anhydrous K_2_CO_3_ (250 mg) was added to a solution of the previously prepared Schiff bases (**12a-j**) (1 mmol) and 4-(chloromethyl)-7-methoxy-2*H*-chromen-2-one (**9**) in acetone (30 mL). The reaction mixture was boiled at 65 °C for 24 h. The mixture was cooled, and ice water was added to form precipitates. The solvent was removed by vacuum filtration, washed several times with cold water, dried, and recrystallized to afford the desired hybrids (**13a-j**).

##### (*E*)-7-Methoxy-4-((4-((phenylimino)methyl)phenoxy)methyl)-2*H*-chromen-2-one (**13a**)

Pale yellow powder; yield: (0.21 g, 55%); R*_f_* = 0.37 in hexane: CH_2_Cl_2_ (2:1); m.p 147–149 °C. IR (KBr) (υ _max_/cm^−1^): 3064 (C-H *sp^2^*), 2940 (C-H *sp^3^*), 1704 (C=O), 1609 (C=N), 1574 and 1510 (C=C aromatic). ^1^H NMR (400 MHz, DMSO-d_6_): δ 3.87 (3H, s, OCH_3_), 5.49 (2H, s, OCH_2_), 6.43 (1H, s, H-3”), 7.00 (1H, dd, *J* = 2.4 and 8.8 Hz, H-6”), 7.05 (1H, d, *J* = 2.4 Hz, H-8”), 7.21 (3H, m, H-3′, H-4′ and H-5′), 7.28 (2H, d, *J* = 8.8 Hz, H-3 and H-5), 7.39 (2H, t, *J* = 7.8, H-2′ and H-6′), 7.79 (1H, d, *J* = 8.8 Hz, H-5”), 7.92 (2H, d, *J* = 8.8 Hz, H-2 and H-6), 8.55 (1H, s, CH=N). ^13^C NMR (100 MHz, DMSO-d_6_): δ 163.0, 160.6, 160.5, 160.2, 155.5, 152.1, 151.5, 130.9, 130.2, 129.6, 126.4, 126.0, 121.3, 115.7, 112.7, 110.8, 109.4, 101.4, 65.8, 56.4. HRMS (ESI): calcd. for C_24_H_19_NO_4_ [M + H]^+^ 385.1314, found 385.1312.

##### (*E*)-7-Methoxy-4-((4-(((4-methoxyphenyl)imino)methyl)phenoxy)methyl)-2*H*-chromen-2-one (**13b**)

Beige powder; yield: (0.13 g, 30%); R*_f_* = 0.42 in hexane:CH_2_Cl_2_ (2:1); m.p 162–163 °C. IR (KBr) (υ _max_/cm^−1^): 3076 (C-H *sp^2^*), 2956 (C-H *sp^3^*), 1708 (C=O), 1607 (C=N), 1574 and 1506 (C=C aromatic). ^1^H NMR (400 MHz, DMSO-d_6_): δ 3.78 (3H, s, OCH_3_), 3.88 (3H, s, OCH_3_), 5.48 (2H, s, OCH_2_), 6.44 (1H, s, H-3”), 6.96 (2H, d, *J* = 8.8 Hz, H-3 and H-5), 7.01 (1H, dd, *J* = 2.4 and 8.8 Hz, H-6”), 7.06 (1H, d, *J* = 2.4 Hz, H-8”), 7.25 (4H, m, H-2′, H-3′, H-5′ and H-6′), 7.80 (1H, d, *J* = 8.8 Hz, H-5”), 7.90 (2H, d, *J* = 8.8 Hz, H-2 and H-6), 8.57 (1H, s, CH=N). ^13^C NMR (100 MHz, DMSO-d_6_): δ 163.0, 160.5, 160.3, 158.1, 158.0, 155.5, 151.5, 144.8, 130.6, 130.5, 126.4, 122.6, 115.6, 114.8, 112.7, 110.8, 109.4, 101.4, 65.8, 56.4, 55.7. HRMS (ESI): calcd. for C_25_H_21_NO_5_ [M + H]^+^ 415.1420, found 415.1424.

##### (*E*)-4-((4-(((4-Chlorophenyl)imino)methyl)phenoxy)methyl)-7-methoxy-2*H*-chromen-2-one (**13c**)

Brown powder; yield: (0.33 g, 78%); R*_f_* = 0.33 in hexane:CH_2_Cl_2_ (2:1); m.p 161–163 °C. IR (KBr) (υ _max_/cm^−1^): 3071 (C-H *sp^2^*), 2936 (C-H *sp^3^*), 1699 (C=O), 1605 (C=N), 1574 and 1508 (C=C aromatic). ^1^H NMR (400 MHz, DMSO-d_6_): δ 3.87 (3H, s, OCH_3_), 5.48 (2H, s, OCH_2_), 6.43 (1H, s, H-3”), 7.00 (1H, dd, *J* = 1.2 and 8.8 Hz, H-6”), 7.05 (1H, d, *J* = 2.0 Hz, H-8”), 7.25 (2H, d, *J*= 8.4 Hz, H-3 and H-5), 7.28 (2H, d, *J*= 8.4 Hz, H-2′ and H-6′), 7.44 (2H, d, *J*= 8.4 Hz, H-3′ and H-5′), 7.79 (1H, d, *J* = 8.8 Hz, H-5”), 7.92 (2H, d, *J*= 8.4 Hz, H-2 and H-6), 8.56 (1H, s, CH=N). ^13^C NMR (100 MHz, DMSO-d_6_): δ 163.0, 160.9, 160.7, 160.5, 155.4, 151.4, 150.9, 131.1, 130.3, 129.9, 129.5, 129.5, 123.2, 115.7, 112.7, 110.8, 109.4, 101.4, 65.8, 56.4. HRMS (ESI): calcd. for C_24_H_18_ClNO_4_ [M + H]^+^ 419.0924, found 419.0920.

##### (*E*)-4-((4-(((4-Chlorophenyl)imino)methyl)-2-methoxyphenoxy)methyl)-7-methoxy-2*H*-chromen-2-one (**13d**)

Pale yellow powder; yield: (0.26 g, 58%); R*_f_* = 0.40 in hexane: CH_2_Cl_2_ (2:1); m.p 173–175 °C. IR (KBr) (υ _max_/cm^−1^): 3078 (C-H *sp^2^*), 2958 (C-H *sp^3^*), 1703 (C=O), 1614 (C=N), 1579 and 1511 (C=C aromatic). ^1^H NMR (400 MHz, DMSO-d_6_): δ 3.87 (3H, s, OCH_3_), 3.89 (3H, s, OCH_3_), 5.46 (2H, s, OCH_2_), 6.40 (1H, s, H-3”), 6.99 (1H, dd, *J* = 2.4 and 8.8 Hz, H-6”), 7.04 (1H, d, *J* = 2.4 Hz, H-8”), 7.26 (2H, d, *J* = 8.8 Hz, H-2′ and H-6′), 7.33 (1H, d, *J* = 8.4 Hz, H-6), 7.44 (2H, d, *J* = 8.8 Hz, H-3′ and H-5′), 7.47 (1H, dd, *J* = 1.6 and 8.4 Hz, H-5), 7.61 (1H, d, *J* = 2.0 Hz, H-3), 7.78 (1H, d, *J* = 8.8 Hz, H-5”), 8.53 (1H, s, CH=N). ^13^C NMR (100 MHz, DMSO-d_6_): δ 163.0, 161.2, 160.6, 155.4, 151.6, 150.8, 150.4, 149.7, 130.3, 130.2, 129.5, 126.4, 124.3, 123.2, 113.8, 112.7, 110.8, 110.5, 109.2, 101.4, 66.3, 56.4, 56.2. HRMS (ESI): calcd. for C_25_H_20_ClNO_5_ [M + H]^+^ 449.1030, found 449.1028.

##### (*E*)-7-Methoxy-4-((2-methoxy-4-((phenylimino)methyl)phenoxy)methyl)-2*H*-chromen-2-one (**13e**)

White powder; yield: (0.28 g, 67%); R*_f_* = 0.47 in hexane:CH_2_Cl_2_ (2:1); m.p 177–178 °C. IR (KBr) (υ _max_/cm^−1^): 3079 (C-H *sp^2^*), 2939 (C-H *sp^3^*), 1705 (C=O), 1611 (C=N), 1579 and 1507 (C=C aromatic). ^1^H NMR (400 MHz, DMSO-d_6_): δ 3.87 (3H, s, OCH_3_), 3.90 (3H, s, OCH_3_), 5.45 (2H, s, OCH_2_), 6.41 (1H, s, H-3”), 6.99 (1H, dd, *J* = 2.4 and 8.8 Hz, H-6”), 7.04 (1H, d, *J* = 2.0 Hz, H-8”), 7.23 (3H, m, H-3′, H-4′ and H-5′), 7.33 (1H, d, *J* = 8.4 Hz, H-6), 7.39 (2H, t, *J* = 7.6 Hz, H-2′ and H-6′), 7.47 (1H, d, *J* = 8.4 Hz, H-5), 7.62 (1H, s, H-3), 7.78 (1H, d, *J* = 8.8 Hz, H-5”), 8.52 (1H, s, CH=N). ^13^C NMR (100 MHz, DMSO-d_6_): δ 163.0, 160.5, 160.5, 155.4, 152.0, 151.6, 150.3, 149.7, 130.5, 129.6, 126.4, 126.1, 124.0, 121.3, 113.8, 112.7, 110.8, 110.5, 109.2, 101.4, 66.3, 56.4, 56.2. HRMS (ESI): calcd. for C_25_H_21_NO_5_ [M + H]^+^ 415.1421, found 415.1420.

##### (*E*)-7-Methoxy-4-((2-methoxy-4-(((4-methoxyphenyl)imino)methyl)phenoxy)methyl)-2*H*-chromen-2-one (**13f**)

White powder; yield: (0.31 g, 69.3%); R*_f_* = 0.51 in hexane:CH_2_Cl_2_ (2:1); m.p 183–184 °C. IR (KBr) (υ _max_/cm^−1^): 3077 (C-H *sp^2^*), 2917 (C-H *sp^3^*), 1717 (C=O), 1610 (C=N), 1579 and 1505 (C=C aromatic). ^1^H NMR (400 MHz, DMSO-d_6_): δ 3.77 (3H, s, OCH_3_), 3.88 (3H, s, OCH_3_), 3.90 (3H, s, OCH_3_), 5.47 (2H, s, OCH_2_), 6.41 (1H, s, H-3”), 6.96 (2H, d, *J* = 8.8 Hz, H-3′ and H-5′), 7.00 (1H, dd, *J* = 2.4 and 8.8 Hz, H-6”), 7.05 (1H, d, *J* = 2.4 Hz, H-8”), 7.26 (2H, d, *J* = 8.8 Hz, H-2′ and H-6′), 7.32 (1H, d, *J* = 8.4 Hz, H-6), 7.44–7.46 (1H, dd, *J* = 2.0 and 8.4 Hz, H-5), 7.60 (1H, d, *J* = 1.6 Hz, H-3), 7.80 (1H, d, *J* = 8.8 Hz, H-5”), 8.55 (1H, s, CH=N). ^13^C NMR (100 MHz, DMSO-d_6_): δ 163.0, 160.5, 158.3, 158.1, 155.4, 151.7, 150.0, 149.7, 144.7, 130.8, 126.5, 123.6, 122.7, 114.8, 113.8, 112.7, 110.8, 110.3, 109.2, 101.4, 66.3, 56.4, 56.1, 55.7. HRMS (ESI): calcd. for C_26_H_23_NO_6_ [M + H]^+^ 445.1525, found 445.1519.

##### (*E*)-7-Methoxy-4-((3-(((4-methoxyphenyl)imino)methyl)phenoxy)methyl)-2*H*-chromen-2-one (**13g**)

Brown powder; yield: (0.30 g, 72%); R*_f_* = 0.52 in hexane:CH_2_Cl_2_ (2:1); m.p 198–199 °C. IR (KBr) (υ _max_/cm^−1^): 3067 (C-H *sp^2^*), 2935 (C-H *sp^3^*), 1710 (C=O), 1612 (C=N), 1578 and 1503 (C=C aromatic). ^1^H NMR (400 MHz, DMSO-d_6_): δ 3.78 (3H, s, OCH_3_), 3.87 (3H, s, OCH_3_), 5.46 (2H, s, OCH_2_), 6.44 (1H, s, H-3”), 6.97 (3H, m, H-3′, H-5′ and H-6”), 7.04 (1H, d, *J* = 2.4 Hz, H-8”), 7.29 (3H, m, H-2′, H-4 and H-6′), 7.45 (1H, t, *J* = 7.8 Hz, H-5), 7.55 (1H, d, *J* = 7.6 Hz, H-6), 7.67 (1H, s, H-3), 7.79 (1H, d, *J* = 8.8 Hz, H-5”), 8.52 (1H, s, CH=N). ^13^C NMR (100 MHz, DMSO-d_6_): δ 163.0, 160.5, 158.5, 158.4, 158.2, 155.5, 141.7, 144.4, 138.4, 130.5, 126.5, 122.8, 122.4, 118.8, 118.3, 114.9, 112.7, 110.8, 109.5, 101.4, 65.8, 56.4, 55.7. HRMS (ESI): calcd. for C_25_H_21_NO_5_ [M + H]^+^ 415.1420, found 415.1417.

##### (*E*)-7-Methoxy-4-((2-methoxy-5-(((4-methoxyphenyl)imino)methyl)phenoxy)methyl)-2*H*-chromen-2-one (**13h**)

Yellow powder; yield: (0.36 g, 80%); R*_f_* = 0.67 in hexane:CH_2_Cl_2_ (2:1); m.p 166–168 °C. IR (KBr) (υ _max_/cm^−1^): 3073 (C-H *sp^2^*), 2935 (C-H *sp^3^*), 1728 (C=O), 1612 (C=N), 1577 and 1507 (C=C aromatic). ^1^H NMR (400 MHz, DMSO-d_6_): δ 3.76 (3H, s, OCH_3_), 3.86 (3H, s, OCH_3_), 3.88 (3H, s, OCH_3_), 5.43 (2H, s, OCH_2_), 6.43 (1H, s, H-3”), 6.94 (2H, d, *J* = 8.8 Hz, H-3′ and H-5′), 6.99 (1H, d, *J* = 2.0 Hz, H-6”), 7.02 (1H, d, *J* = 2.8 Hz, H-8”), 7.14 (1H, d, *J* = 8.8 Hz, H-3), 7.22 (2H, d, *J* = 8.8 Hz, H-2′ and H-6′), 7.50 (1H, d, *J* = 8.4 Hz, H-4), 7.73 (1H, s, H-6), 7.77 (1H, d, *J* = 9.2 Hz, H-5”), 8.50 (1H, s, CH=N). ^13^C NMR (100 MHz, DMSO-d_6_): δ 162.9, 160.6, 158.3, 158.0, 155.4, 152.2, 151.8, 147.6, 144.7, 129.7, 126.5, 124.7, 122.6, 115.5, 114.8, 112.7, 112.3, 110.9, 109.4, 101.3, 66.4, 56.3, 56.3, 55.7. HRMS (ESI): calcd. for C_26_H_23_NO_6_ [M + H]^+^ 445.1525, found 445.1512.

##### (*E*)-4-((5-(((4-Chlorophenyl)imino)methyl)-2-methoxyphenoxy)methyl)-7-methoxy-2*H*-chromen-2-one (**13i**)

Beige powder; yield: (0.27 g, 60%); R*_f_* = 0.44 in hexane:CH_2_Cl_2_ (2:1); m.p 156–157 °C. IR (KBr) (υ _max_/cm^−1^): 3086 (C-H *sp^2^*), 2970 (C-H *sp^3^*), 1704 (C=O), 1614 (C=N), 1578 and 1510 (C=C aromatic). ^1^H NMR (400 MHz, DMSO-d_6_): δ 3.87 (3H, s, OCH_3_), 3.90 (3H, s, OCH_3_), 5.44 (2H, s, OCH_2_), 6.40 (1H, s, H-3”), 6.98 (1H, dd, *J* = 2.4 and 8.8 Hz, H-6”), 7.03 (1H, d, *J* = 2.4 Hz, H-8”), 7.25 (2H, d, *J* = 8.8 Hz, H-2′ and H-6′), 7.33 (1H, d, *J* = 8.4 Hz, H-3), 7.43 (2H, d, *J* = 8.8 Hz, H-3′ and H-5′), 7.46 (1H, dd, *J* = 1.6 and 8.4 Hz, H-4), 7.60 (1H, d, *J* = 1.6 Hz, H-6), 7.78 (1H, d, *J* = 8.8 Hz, H-5”), 8.52 (1H, s, CH=N). ^13^C NMR (100 MHz, DMSO-d_6_): δ 163.0, 161.2, 160.5, 155.4, 151.6, 150.8, 150.5, 149.7, 130.3, 130.2, 129.5, 126.4, 124.3, 123.2, 113.8, 112.7, 110.8, 110.5, 109.2, 101.4, 66.3, 56.4, 56.2. HRMS (ESI): calcd. for C_25_H_20_ClNO_5_ [M + H]^+^ 449.1030, found 449.1029.

##### (*E*)-4-((3-(((4-Chlorophenyl)imino)methyl)phenoxy)methyl)-7-methoxy-2*H*-chromen-2-one (**13j**)

Pale yellow powder; yield: (0.32 g, 76%); R*_f_* = 0.29 in hexane: CH_2_Cl_2_ (2:1); m.p 184–186 °C. IR (KBr) (υ _max_/cm^−1^): 3079 (C-H *sp^2^*), 2933 (C-H *sp^3^*), 1703 (C=O), 1612 (C=N), 1579 and 1487 (C=C aromatic). ^1^H NMR (400 MHz, DMSO-d_6_): δ 3.87 (3H, s, OCH_3_), 5.47 (2H, s, OCH_2_), 6.44 (1H, s, H-3”), 6.99 (1H, dd, *J* = 2.0 and 8.8 Hz, H-6”), 7.05 (1H, d, *J* = 2.4 Hz, H-8”), 7.29 (2H, d, *J* = 8.4 Hz, H-2′ and H-6′), 7.32 (1H, dd, *J* = 2.4 and 8.4 Hz, H-4), 7.46 (2H, d, *J* = 8.4 Hz, H-3′ and H-5′), 7.50 (1H, d, *J* = 8.0 Hz, H-5), 7.57 (1H, d, *J* = 7.6 Hz, H-6), 7.70 (1H, s, H-2), 7.79 (1H, d, *J* = 8.8 Hz, H-5”), 8.61 (1H, s, CH=N). ^13^C NMR (100 MHz, DMSO-d_6_): δ 163.0, 161.6, 160.5, 158.4, 155.5, 151.6, 150.5, 148.1, 137.8, 130.6, 129.5, 129.6, 128.9, 123.2, 119.0, 114.6, 112.7, 110.8, 109.5, 101.4, 65.8, 56.4. HRMS (ESI): calcd. for C_24_H_18_ClNO_4_ [M + H]^+^ 419.0924, found 419.0908.

### 3.2. Biological Evaluation of Hybrids against AChE

The procedure described by Ellman [64], slightly modified, was performed to assess the AChE inhibitory activity of synthesized compounds, according to reported procedures in the literature [20,65,66,67]. Detailed protocol is provided in supplementary materials.

### 3.3. Molecular Docking Study

The 3D structures of all synthesized hybrids were previously geometry optimized and then saved in PDBQT file format utilizing Autodock Tools (v1.5.6rc3) [68]. This involves the following steps: developing a torsion tree by detecting and choosing a root and saving it as a pdpqt file for mapping prior to molecular docking simulation. The crystallographic structure of AChE (PDB ID: 4EY7) was downloaded from the protein data bank website (https://www.rcsb.org/structure/4EY7, accessed on 15 June 2023). The protein structure was cleaned from heteroatoms, and the Swiss-PdbViewer was used for molecular mechanics energy minimization [69]. The protein structure was prepared for molecular docking study according to our previous studies and saved in PDBQT format [28,70]. Finally, molecular docking simulation was performed, visualized, and analyzed as previously reported [71,72,73].

### 3.4. Molecular Dynamics Simulation (MDS)

MD simulations were investigated to calculate binding energies and study the relative stabilities of the interactions between the two top hybrids (**13c** and **13d**), consensus docking scores, and target receptors. The simulation studies were carried out utilizing the NAMD (v2.13) suit [74] and the CHARMM36 [75] force field. Furthermore, the latest CHARMM/CGenFF force field was used to generate the parameters and topological files for the selected compounds [19,76]. The complex structure (ligand-protein) was placed in the middle of a box that solvated molecules of water with a TIP3P explicit solvation model. A 0.15 molar solution of (145 Na^+^ and 135 Cl^-^) ions, to mimic the physiological salt concentrations, was added to provide electrostatic screening and charge neutralization which extended 20 Å from the protein. CHARMM and the periodic boundary conditions were set with dimensions of a rectangle-cubic system of 117.0, 117.0, and 117.0 Å in x, y, and z directions, respectively.

The MD studies include minimization, equilibration, and data analysis. There were no atoms restricted in the MD simulations. The isothermal-isobaric (NPT) ensemble and a 2 fs time integration step were selected in this study, and the pressure was set at 1 atm utilizing the Nose’-Hoover Langevin piston barostat [77]. The Langevin thermostat has been used to set the temperature at 300.0 K [78]. The force-field parameters were assumed in order to minimize and equilibrate the complexes in the system, which have a scaling of 1.0 Å. The preliminary energy of the complex was minimized through 2000 steps at 300 K. The temperature, kinetic energy, and/or pressure of the system were controlled by Langevin dynamics simulation through another 144,000 steps. The 500,000 minimization steps were used to equilibrate the solvated system, and 50,000,000 runs for 100 ns. The VMD package was used for the analysis of the output data [79]. A 20.0 Å for the distance cut-off was used for Lennard Jones interactions, and short-range non-bonded interactions with a pair list distance of 12 Å were smoothly truncated at 8.0 Å. The long-range electrostatic interactions were analyzed and visualized using the particle-mesh Ewald (PME) procedure [80], where simulated cells were placed in a grid box with 1.0 Å.

### 3.5. Binding Energy Calculations

The relative binding energy calculations were carried out by the one-average molecular mechanics generalized Born surface area (MM/GBSA) method [81]. The MolAICal [33] was employed to calculate the MM/GBSA for complex (ligands and receptors) based on molecular dynamical (MD) simulated results by NAMD, in which the ligand (L) binds to the target receptor (R) to generate the receptor-ligand (RL). We were only interested in relative binding energies based on MM/GBSA calculations, which were the Gibbs relative binding energy, given by:

$$\Delta G_{\mathrm{bind}}=\Delta G_{\mathrm{RL}}-\Delta G_{R}-\Delta G_{L}$$

### 3.6. Geometry DFT Optimization

During this study, packages of programs were used to run the molecular modeling calculations of all synthesized hybrids utilizing the Gaussian 09W software package [82]. The molecular structures of hybrids were geometrically optimized using density functional theory (DFT) with long-range corrections functional wB97XD (DFT/wB97XD) [83], which includes empirical dispersion with basis set 6-311++G (d,p) [84]. During the geometry optimization, no symmetry restrictions were used [85,86]. The choice of long-range corrections functional wB97XD with a large basis set was due to the accuracy, consistency, flexibility, and better performance of Grimme’s D2 dispersion model, which includes empirical dispersion [83,87]. The same level of theory has been applied to calculate the vibrational frequency for each compound, and the molecular structures of each hybrid were found to correspond to real minima of the potential energy surface [88]. In order to identify the reactive site of the molecules, the DFT/wB97XD was employed to describe reactivity descriptors and molecular stability. A descriptor of local reactivity was computed using the Fukui function and the dual descriptor [40,42,47].

Furthermore, the quantum chemical descriptors from conceptual density functional theory (CDFT) were calculated by utilizing the Multiwfn (v3.7) package [41]. The electrostatic potential (ESP) of the molecules was rendered by the Visual Molecular Dynamics package (VMD 1.9 program) based on the data outputted by the Multiwfn program [50,79]. Natural bond orbital (NBO) analyses have been calculated utilizing NBO 3.1, which is provided in the Gaussian 09W program. The GaussView (v6.1) [89] and ChemCraft (v1.6) packages [90] were used to visualize the optimized structure and molecular orbitals. The QSAR features included in the HyperChem program (v8.0.7) [91] were used to determine the SAR properties of all synthesized compounds.

## 4. Conclusions

A series of Schiff base-coumarin hybrids (**13a-j**) were designed with the assistance of a molecular docking study. The affinity energy of hybrids (**13a-j**) on the active site of acetylcholinesterase (AChE) was in the range of −10.6 to −13.2 kcal/mol, while the affinity energy of positive controls, donepezil and galantamine, was recorded at −11.4 and −9.6 kcal/mol, respectively. The obtained results showed that most of the designed hybrids were more active than the positive controls. Hybrids were synthesized and bio-evaluated against AChE. Results showed that most of them could potentially inhibit the target enzyme. Compounds **13c** and **13d** with IC_50_ values of 0.232 ± 0.011 and 0.190 ± 0.004 yielded the lowest IC_50_ values and were 5-fold stronger than the positive control. The detailed insights into the electronic structure properties, its link to drug-like properties, and the structure-activity relationships of the designed compounds were provided by applying the high level of computational approaches of DFT/wB97XD methods by using 6-311++G(d,p) as the basis set wave function. Moreover, the molecular docking studies revealed that the synthesized compounds exhibit molecular interactions via hydrogen bonds with GLY121, GLY122, TYR132, SER203, PHE295, and TYR337 amino acid residues of the target enzyme. Careful inspection of the pattern of binding sites and binding energy indicated **13c** and **13d** compounds could be good candidates for AChE inhibitors, which is correlated with experimental results. The drug likeness and QSAR descriptors show that compound **13f** has the highest value, while compound **13a** has the lowest values in all descriptors for other derivatives. All target compounds except **13c** and **13j** have good aqueous solubility. Furthermore, *Log P* values of compounds **13f** = **13h** < **13d** = **13i** < **13b** = **13e = 13g** < **13a** are in the field of optimal values (0 < *Log P* < 5). Based on these, it can be stated that these compounds have good oral bioavailability and optimal biological activity. The stability of the systems was measured using RMSD during the 100 ns simulations. Obtained results revealed that the **13d** complex system acquired a relatively more stable conformation than the **13c** complex. The calculated average RMSF values for the **13c**-AChE and **13d**-AChE complexes to protein systems were 0.85 and 0.83 Å, respectively, which demonstrate that the system inhibition of the **13d**-AChE protein complex system is lower than the other systems inhibition, which will reflect well on the complex stability. The binding free energy technique (MM/GBSA) of the simulated complex was computed, and it was found to be −23.645 and −36.042 kcal/mol for complexes **13c** and **13d**, respectively. Thus, it is suggested that the stability of the **13d**-4EY7 complex over the other is better. The overall study indicates that **13d** has comparable and even better descriptors than Galantamine and can be a potential AChE candidate.

## Figures and Tables

**Table 1:** The dock scores and IC_50_ values (μM) of compounds (**13a-j**) and galantamine against AChE.

Compounds	Binding Energy (kcal/mol)	IC_50_ [µM]
**13a**	−11.9	0.440 ± 0.016
**13b**	−11.7	0.466 ± 0.007
**13c**	−13.2	0.232 ± 0.011
**13d**	−13.2	0.190 ± 0.004
**13e**	−13.1	0.297 ± 0.006
**13f**	−12.7	0.365 ± 0.025
**13g**	−11.5	1.090 ± 0.058
**13h**	−10.6	1.175 ± 0.063
**13i**	−11.7	0.712 ± 0.044
**13j**	−12.3	0.651 ± 0.003
**GAL.**	−9.6	1.142 ± 0.027

Data are expressed as mean ± Standard Deviation of seven independent experiments performed in triplicate.

**Table 2:** Molecular docking scores and interaction modes between synthesized compounds (**13a-j**) against AChE (PDB ID: 4EY7).

Compound	Binding Energy (Kcal/mol)	Interactions					
		H-Bond	Hydrophobic				Electrostatic or Other
			Alkyl	π-Alkyl	π-Sigma	π-π T-Shape	π-anion/π-Donor/Carbon H Bond
**13a**	−11.9	GLY121, GLY122, SER203, PHE295	ALA204, TRP236, PHE297	VAL294	His447	TYR337, TRP286, TYR341, PHE338	
**13b**	−11.7	SER203, GLY122	LEU289, ILE451,		VAL294, PHE338	TRP286, TRP86, HIS447	GLU202/TYP133
**13c**	−13.2	GLY121, SER203, TYR337		TYR72, TYR124, TRP86, TRP286		TRP286, TYR341, TRP86	TRP86, TYR341
**13d**	−13.2	GLY121, SER203, TYR337		LEU289		TRP286, TYR341, TRP86	TRP86, TYR341, SER293
**13e**	−13.1	GLY122, TYR133	PRO88, PHE297,	VAL294	VAL294		PHE295, HIS447, GLY126, TYR124, SER125, GLN71, TYR72, TRP86
**13f**	−12.7	TYR133	PHE297, ALA204, HIS447, PRO88, TRP286		TRP86	TRP86	PHE295, GLY126, TYR124, SER125, GLN71, TYR72, ARG296, SER293, VAL294
**13g**	−11.5			TYR124	VAL294	TRP286, TYR337, PHE338	TYR72, TYR124, TYR341
**13h**	−10.6	SER203	TRP286, TRP86, PHE297, LEU289, VAL294, TYR337	VAL294	TRP236	TRP86, TYR341	PHE295, TYR124, ARG296, SER293, HIS447
**13i**	−11.7	TYR133	PRO88, TYR387, TRP86	TRP286, VAL294	TRP86	TYR124	PHE295, GLY126, TYR124, SER125, GLN71, TYR72, HIS447
**13j**	−12.3	TYR124, TYR337	PRO88	LEU289, TRP286	VAL294	PHE338, TRP286, TYR341	ASP74/TRP86, GLN71, TYR341,

**Table 3:** The physicochemical properties analysis and QSAR properties of the synthesized compounds (**13a-j**) against AChE.

Compounds	Polarizability (A^3^)	Refractivity (A^3^)	Vol (A^3^)	Surface Area (Grid) A*^2^*	HE (kcal/mol)	Log P	MW (DA)
**13a**	44.00	110.22	1111.03	668.68	−11.09	4.53	385.42
**13b**	46.47	116.68	1187.79	704.70	−12.67	4.28	415.45
**13c**	45.93	115.02	1156.57	692.72	−10.72	5.05	419.86
**13d**	48.40	121.48	1231.94	732.87	−10.84	4.80	449.89
**13e**	46.47	116.68	1188.57	708.71	−11.21	4.28	415.45
**13f**	48.94	123.14	1263.19	750.75	−12.78	4.03	445.47
**13g**	46.47	116.68	1187.79	704.73	−12.52	4.28	415.45
**13h**	48.94	123.14	1260.71	754.84	−12.58	4.03	445.47
**13i**	48.40	121.48	1230.12	732.60	−10.62	4.80	449.89
**13j**	45.93	115.02	1156.40	690.79	−10.57	5.05	419.86

**Table 4:** Summary of the binding energy calculated for two top docking binding scores hybrids (**13c** and **13d**) with target receptor AChE (PDB ID: 4EY7).

Complexes	ΔΕ_VDW_	ΔΕ_ele_ + ΔG_sol_	ΔG_bin_
**13c**-AChE	−44.913	12.267	−32.645 ± 0.119
**13d**-AChE	−51.081	15.039	−36.042 ± 0.121

**Δ**Ε_VDW_ = van der Waals energy; **Δ**Ε_ele_ = electrostatic energy; **Δ**G_sol_ = solvation free energy; **Δ**G_bin_ = calculated total binding free energy (kcal/mol).

**Table 5:** Natural charge of selected atoms of newly synthesized compounds (**13a-j**) using wb97xd/6-311++g(d,p) level of theory.

	13a	13b	13c	13d	13e	13f	13g	13h	13i	13j
**O1**	−0.519	−0.519	−0.518	−0.520	−0.521	−0.521	−0.519	−0.521	−0.521	−0.519
**C2**	0.774	0.774	0.774	0.774	0.774	0.774	0.774	0.775	0.774	0.774
**C3**	−0.313	−0.314	−0.313	−0.319	−0.319	−0.319	−0.313	−0.318	−0.317	−0.313
**C4**	0.032	0.033	0.032	0.039	0.040	0.040	0.034	0.039	0.038	0.033
**C5**	−0.175	−0.175	−0.175	−0.174	−0.173	−0.173	−0.174	−0.173	−0.173	−0.175
**C6**	−0.138	−0.138	−0.138	−0.136	−0.136	−0.136	−0.138	−0.137	−0.137	−0.138
**C7**	−0.314	−0.314	−0.314	−0.316	−0.316	−0.316	−0.315	−0.316	−0.316	−0.315
**C8**	0.357	0.357	0.357	0.355	0.355	0.354	0.356	0.354	0.355	0.357
**C9**	−0.277	−0.277	−0.277	−0.279	−0.279	−0.279	−0.278	−0.279	−0.279	−0.278
**C10**	0.380	0.380	0.380	0.380	0.380	0.380	0.380	0.380	0.380	0.380
**O11**	−0.562	−0.563	−0.562	−0.566	−0.567	−0.567	−0.563	−0.567	−0.566	−0.562
**O16**	−0.535	−0.535	−0.535	−0.536	−0.537	−0.537	−0.536	−0.537	−0.536	−0.535
**C17**	−0.211	−0.211	−0.211	−0.211	−0.210	−0.210	−0.211	−0.210	−0.210	−0.211
**C21**	−0.043	−0.043	−0.043	−0.051	−0.051	−0.051	−0.042	−0.047	−0.047	−0.042
**O24**	−0.547	−0.548	−0.546	−0.571	−0.572	−0.572	−0.553	−0.572	−0.572	−0.552
**C25**	0.344	0.342	0.346	0.287	0.285	0.282	0.326	0.270	0.271	0.327
**C26**	−0.246	−0.246	−0.246	−0.222	−0.217	−0.222	−0.195	−0.183	−0.181	−0.192
**C27**	−0.131	−0.132	−0.130	−0.165	−0.177	−0.168	−0.096	−0.140	−0.146	−0.103
**C28**	−0.159	−0.155	−0.162	−0.124	−0.115	−0.117	−0.208	−0.150	−0.147	−0.206
**C29**	−0.136	−0.138	−0.134	−0.257	−0.252	−0.261	−0.182	−0.299	−0.300	−0.182
**C30**	−0.310	−0.310	−0.310	0.290	0.287	0.290	−0.285	0.310	0.314	−0.281
**C34**	0.154	0.146	0.157	0.171	0.151	0.158	0.140	0.145	0.156	0.153
**N36**	−0.453	−0.450	−0.456	−0.455	−0.444	−0.450	−0.436	−0.449	−0.455	−0.441
**C37**	0.135	0.099	0.133	0.129	0.132	0.095	0.095	0.099	0.132	0.130
**C38**	−0.209	−0.180	−0.192	−0.190	−0.207	−0.177	−0.175	−0.180	−0.191	−0.189
**C39**	−0.195	−0.289	−0.218	−0.218	−0.196	−0.291	−0.290	−0.289	−0.218	−0.217
**C40**	−0.221	0.320	−0.050	−0.048	−0.219	0.323	0.324	0.321	−0.049	−0.048
**C41**	−0.196	−0.237	−0.218	−0.218	−0.196	−0.237	−0.237	−0.238	−0.219	−0.218
**C42**	−0.238	−0.213	−0.221	−0.220	−0.237	−0.211	−0.210	−0.213	−0.221	−0.219
**A47**	0.208	−0.545	−0.003	−0.001	0.208	−0.545	−0.545	−0.545	−0.003	−0.001
**A48**	0.213	0.213	0.214	−0.546	−0.547	−0.547	0.212	−0.538	−0.537	0.213

A=O, H, or Cl means different atoms in derivatives series; Values are mean ± SD triplicate assays.

**Table 6:** Energetic parameters of synthesized derivatives (**13a-j**) using wb97xd/6-311++g(d,p) level of theory.

Parameters	ET, au	EHOMO, au	ELUMO, au	Eg, eV	μ, D	I, eV	A, eV
**13a**	−1281.51218	−0.29732	−0.00966	7.83	8.73	8.09	0.26
**13b**	−1396.03187	−0.27977	−0.00871	7.38	8.18	7.61	0.24
**13c**	−1741.11979	−0.29937	−0.01125	7.84	8.50	8.15	0.31
**13d**	−1855.63488	−0.30068	−0.01208	7.85	8.54	8.18	0.33
**13e**	−1396.02739	−0.29860	−0.00589	7.96	9.31	8.13	0.16
**13f**	−1510.54717	−0.28213	−0.00252	7.61	9.78	7.68	0.07
**13g**	−1396.03041	−0.28300	−0.00735	7.50	7.68	7.70	0.20
**13h**	−1510.54851	−0.27929	−0.00012	7.60	8.42	7.60	0.00
**13i**	−1855.63633	−0.29807	−0.00737	7.91	9.89	8.11	0.20
**13j**	−1741.11801	−0.30477	−0.01351	7.93	10.33	8.29	0.37

Values are mean ± SD duplicate assays.

**Table 7:** Reactivity indices of hybrids (**13a-j**) using wb97xd/6-311++g(d,p) level of theory.

Parameters	X, eV	η, eV	S, eV	V, eV	ω, eV	N, eV
**13a**	4.18	3.91	0.128	−4.18	2.23	−3.88
**13b**	3.92	3.69	0.136	−3.92	2.09	−3.40
**13c**	4.23	3.92	0.128	−4.23	2.28	−3.94
**13d**	4.26	3.93	0.127	−4.26	2.31	−3.97
**13e**	4.14	3.98	0.126	−4.14	2.15	−3.91
**13f**	3.87	3.80	0.131	−3.87	1.97	−3.47
**13g**	3.95	3.75	0.133	−3.95	2.08	−3.49
**13h**	3.80	3.80	0.132	−3.80	1.90	−3.39
**13i**	4.16	3.96	0.126	−4.16	2.18	−3.90
**13j**	4.33	3.96	0.126	−4.33	2.37	−4.08

Values are mean ± SD triplicate assays.

## Data Availability

Data is contained within the article and supplementary material.

## Supplementary Materials

The following supporting information can be downloaded at: The following supporting information can be downloaded at: https://www.mdpi.com/article/10.3390/ph16070971/s1, Detailed description of biological evaluation protocol and IC_50_ calculation, Inhibition diagrams for the compounds, FTIR spectra of the compounds, 1H and 13C spectra of the compounds, HRMS spectra of the compounds, Figure S1: The proposed binding mode of synthesized hybrids (**13a-j**) docked in the active site of acetylcholinesterase (4EY7) protein; (2D and 3D ligand-receptor interactions), Figure S2: The optimized geometry, numbering system, vector of dipole moment of the synthesized compounds, Figure S3: Frontier molecular orbitals of the synthesized compounds, Figure S4: MEP surfaces of the synthesized compounds, Table S1: The selected bond length (Ao), bond angles and dihedral angles, (degree) of the compounds, Table S2: Mean absolute errors computed for selected bond lengths (Ao) and angles (degree) of synthesized compounds, Tables S3 and S4: Values of the Fukui functions and dual descriptor of compounds, Table S5: Values of the condensed local softnesses (Hartree*e) of compounds, Table S6: Values of the condensed local electrophilicity (ElP)/nucleophilicity (NuP) index (e*eV) of compounds.
