# Supplementary material for: Discovery of Novel Coumarin-Schiff Base Hybrids as Potential Acetylcholinesterase Inhibitors: Design, Synthesis, Enzyme Inhibition, and Computational Studies
Source: Pharmaceuticals (Basel). 2023 Jul 6;16(7):971. doi: 10.3390/ph16070971 (PMC10385371; doi:10.3390/ph16070971)
Supplement: Supplementary file 1 [file pharmaceuticals-16-00971-s001.zip › pharmaceuticals-2482301-supplementary.pdf]

Discovery of novel coumarin-Schiff base hybrids as potential acetylcholinesterase inhibitors: Design, synthesis, enzyme inhibition, and computational studies

Aso Hameed Hasan [1,2], Faruq Azeez Abdulrahman [3], Ahmad J. Obaidullah [4], Hadil Faris Alotaibi [5], Mohammed M. Alanazi [4], Mahmoud A. Noamaan [6], Sankaranarayanan Murugesan [7], Syazwani Itri Amran [8], Ajmal R. Bhat [9] and Joazaizulfazli Jamalis [1,*]

[1]Department of Chemistry, Faculty of Science, University Teknologi Malaysia, Johor Bahru 81310, Johor, Malaysia; aso.hameed@garmian.edu.krd
[2]Department of Chemistry, College of Science, University of Garmian, Kalar 46021, Kurdistan Region, Iraq
[3]Department of Pharmacy, Kalar Private Technical Institute, Kalar 46021, Kurdistan Region, Iraq; farouqpharma85@gmail.com
[4]Department of Pharmaceutical Chemistry, College of Pharmacy, King Saud University, Riyadh 11451, Saudi Arabia; aobaidullah@ksu.edu.sa (A.J.O.); mmalanazi@ksu.edu.sa (M.M.A.)
[5]Department of Pharmaceutical Sciences, College of Pharmacy, Princess Nourah bint AbdulRahman University, Riyadh 11671, Saudi Arabia; hfalotaibi@pnu.edu.sa
[6]Mathematics Department, Faculty of Science, Cairo University, Giza 12613, Egypt; noamaan@sci.cu.edu.eg
[7]Medicinal Chemistry Research Laboratory, Birla Institute of Technology & Science Pilani (BITS Pilani), Pilani Campus, Pilani 333031, Rajasthan, India; murugesan@pilani.bits-pilani.ac.in
[8]Department of Biosciences, Faculty of Science, Universiti Teknologi Malaysia, Johor Bahru 81310, Johor, Malaysia; syazwaniitri@utm.my
[9]Department of Chemistry, R.T.M. Nagpur University, 440033, Nagpur, India; bhatajmal@gmail.com

*Corresponding author E-mail: joazaizulfazli@utm.my (Joazaizulfazli Jamalis).

EXPERIMENTAL

In vitro inhibition study on AChE

The assay for AChE inhibitory activity was done based on a modified protocol [1-5]. Acetylcholinesterase (Type VI-S, from Electrophorus electricus), 5,5′-dithiobis-2-nitrobenzoic acid (Ellman's reagent, DTNB) and acetylthiocholine chloride (ATCI) were purchased from Sigma Aldrich. Enzyme solutions were prepared to give 0.28 units/mL aliquots. Stock solutions were prepared by dissolving 1 mg of tested compounds in 1 mL of DMSO and diluted to a final concentration of 1000, 800, 600, 400, 200, 100, and 50 µg/mL. The assay buffer solution A (pH = 8.0) was prepared by taking 93.2 mL of solution (6 g of sodium dihydrogen phosphate was dissolved in 500 mL of deionized water) and mixed with 6.8 mL of solution (7 g of sodium hydrogen phosphate was dissolved in 500 mL of deionized water) and adjusted. 57.7 mL of sodium dihydrogen phosphate and 42.3 mL of sodium hydrogen phosphate were mixed and adjusted to prepare the buffer B (pH = 7.0). Furthermore, fresh solutions of 0.01 M DTNB (0.396 g of DTNB and 15 mg $NaHCO_3$ were dissolved in 100 mL of the buffer B) in a dark place, and 0.075 M ATCI (0.2048 g of ATCI was dissolved in 10 mL of deionized water), were prepared. In 96-well plates, 20 µL of the test compounds, 10 µL of DTNB, 15 µL of the enzyme (AChE) and 140 µL of the buffer solution A, were added in dark conditions and incubated for 15 min, followed by the addition of ATCI (10 µL) and incubated again for the same period. The activity was measured by reading the absorbance of the solution at 412 nm. Blanks containing all the components except the enzyme were carried out. IC_{50} values were calculated as the concentration of a compound that produces 50% enzyme activity inhibition using the GraphPad Prism 8 programme package. Results are expressed as the mean ± SD of at least three different experiments performed in triplicate.

IC_{50} Calculations

Percentage inhibition was calculated by this formula:

$$\% \text{ (Inhibition)} = \frac{B - A}{B} X\ 100$$

- A= absorbance of the enzyme with the test sample;
- B = absorbance of the enzyme without test sample.
- Each experiment was repeated thrice.

- Concentrations were converted from µg/mL to µM.
- IC_{50} value was calculated by GraphPad Prism.
- Galantamine was used as a reference compound.

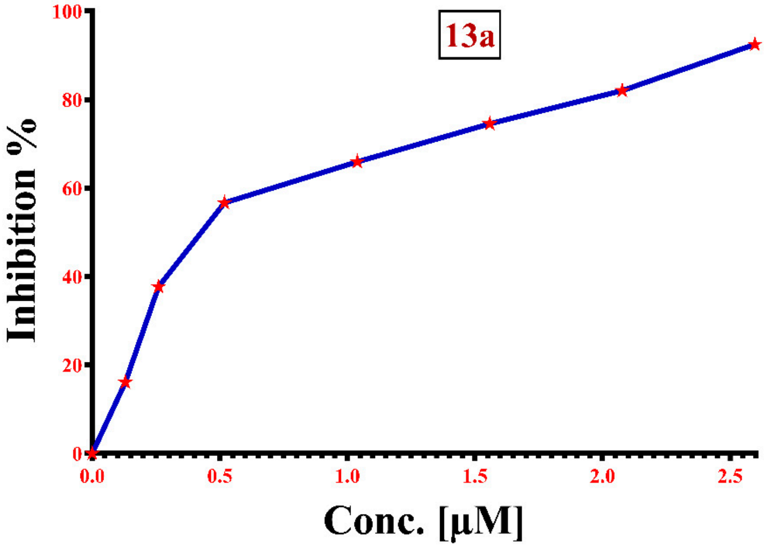

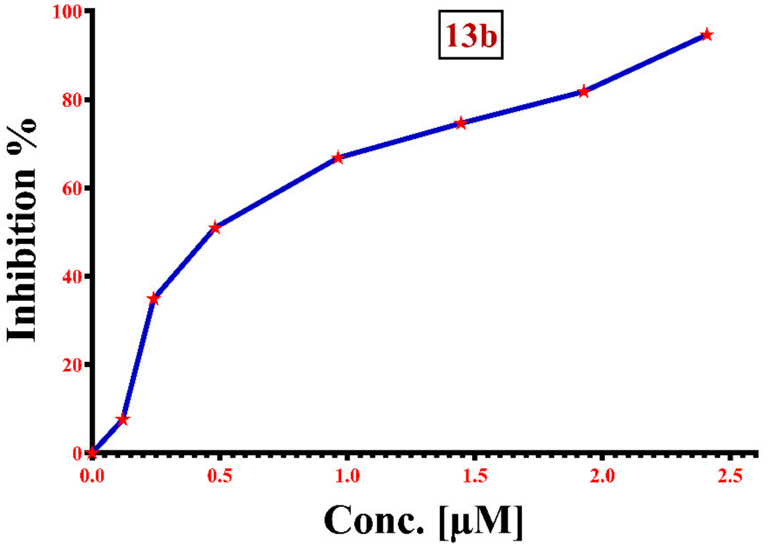

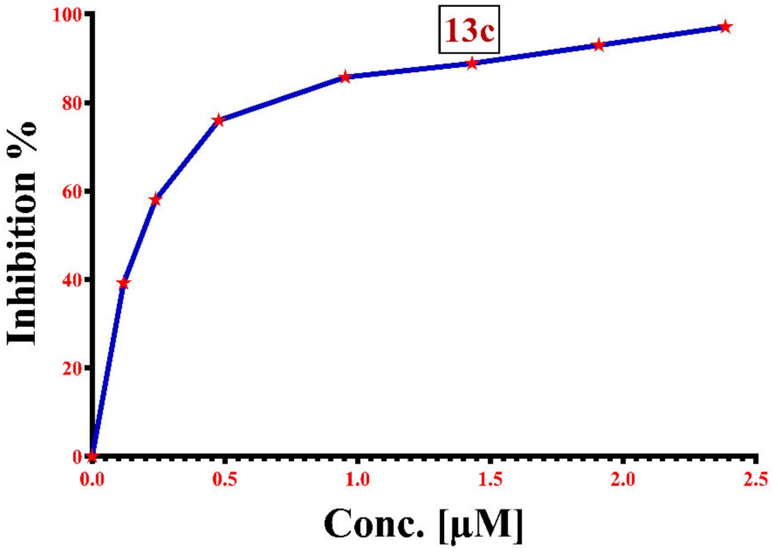

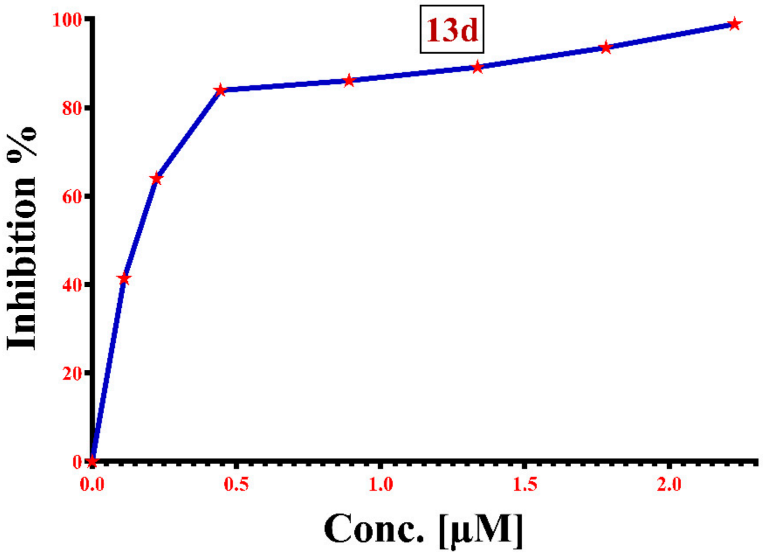
13d
Inhibition %
Conc. [µM]
0
20
40
60
80
100
0.0
0.5
1.0
1.5
2.0

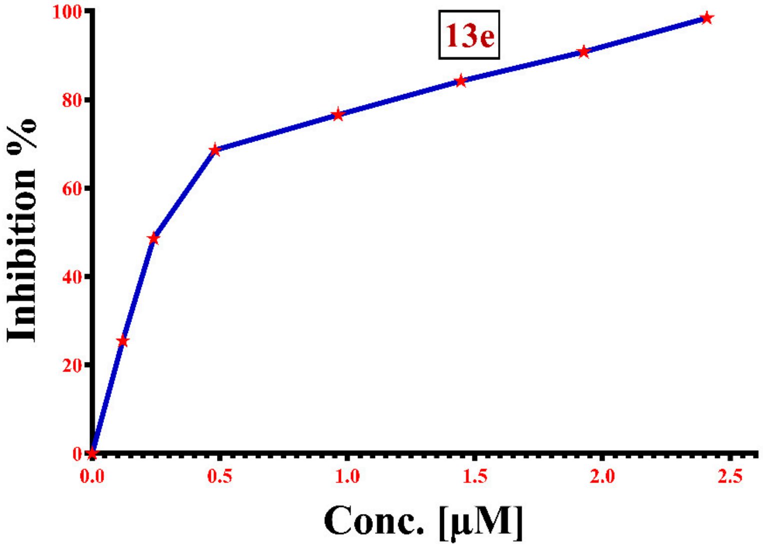
13e
Inhibition %
Conc. [µM]
0
20
40
60
80
100
0.0
0.5
1.0
1.5
2.0
2.5

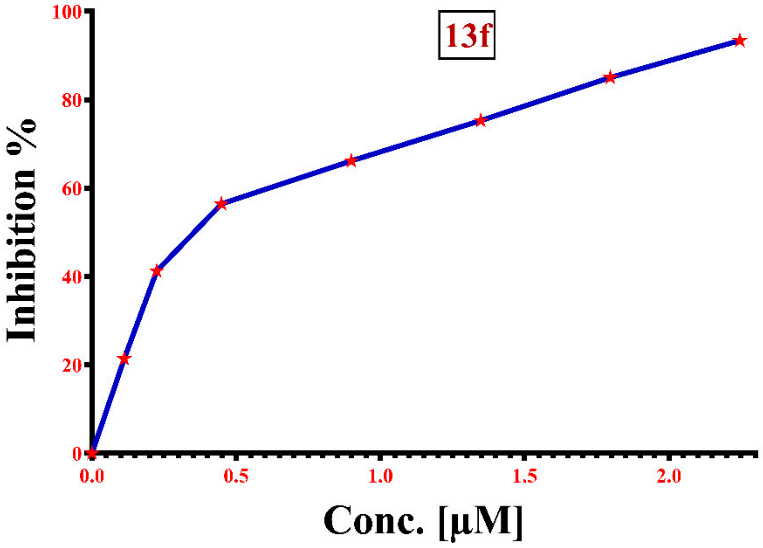
13f
Inhibition %
Conc. [µM]
0
20
40
60
80
100
0.0
0.5
1.0
1.5
2.0

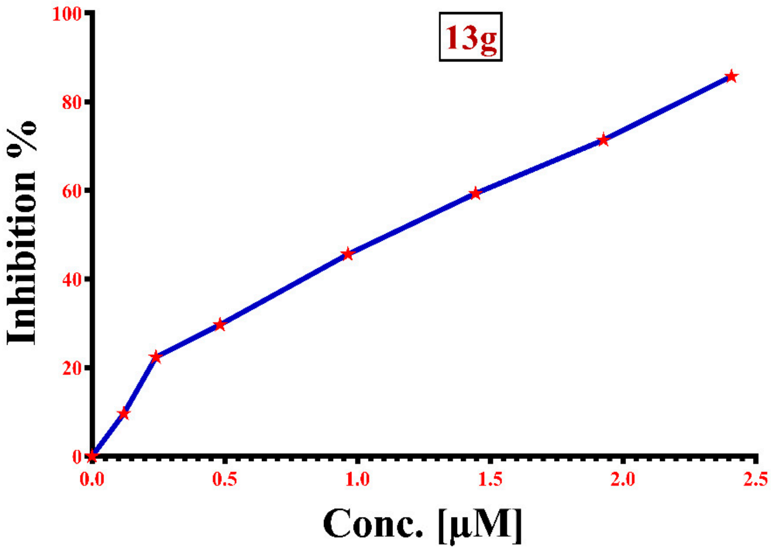
13g
Inhibition %
100
80
60
40
20
0
0.0
0.5
1.0
1.5
2.0
2.5
Conc. [µM]

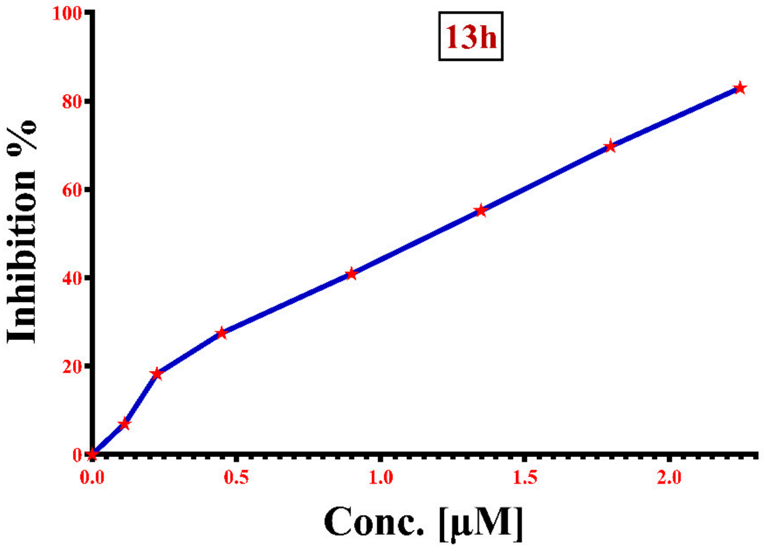
13h
Inhibition %
100
80
60
40
20
0
0.0
0.5
1.0
1.5
2.0
Conc. [µM]

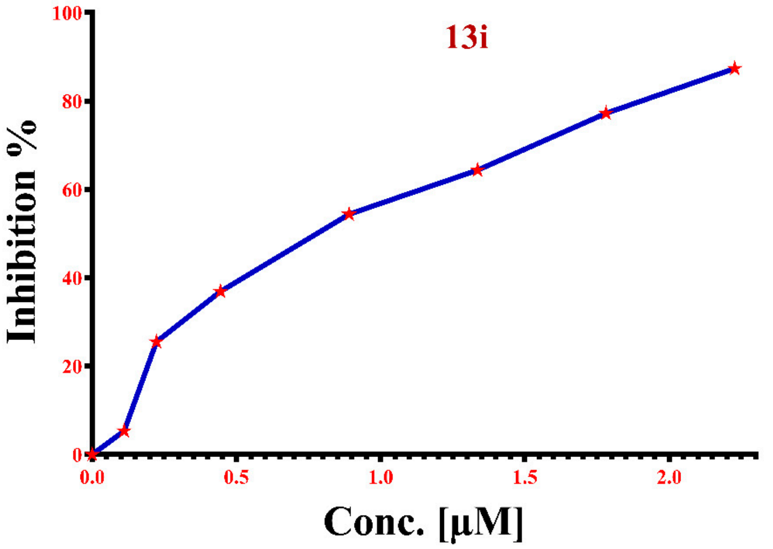
13i
Inhibition %
100
80
60
40
20
0
0.0
0.5
1.0
1.5
2.0
Conc. [µM]

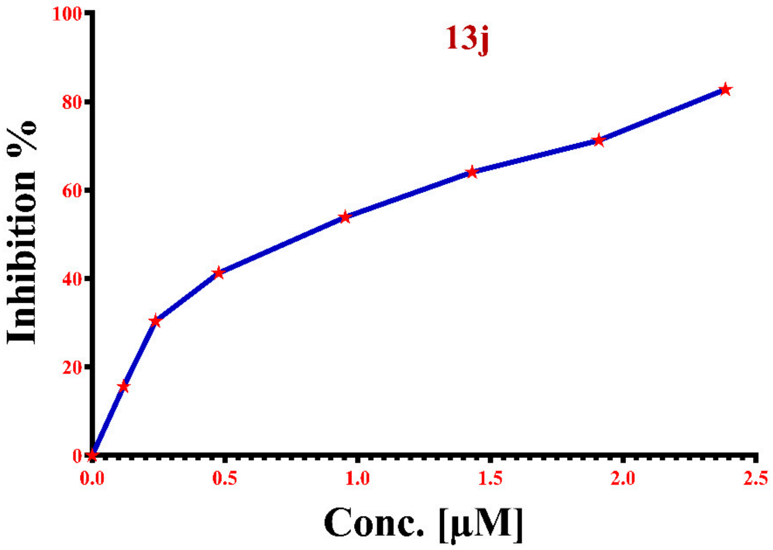

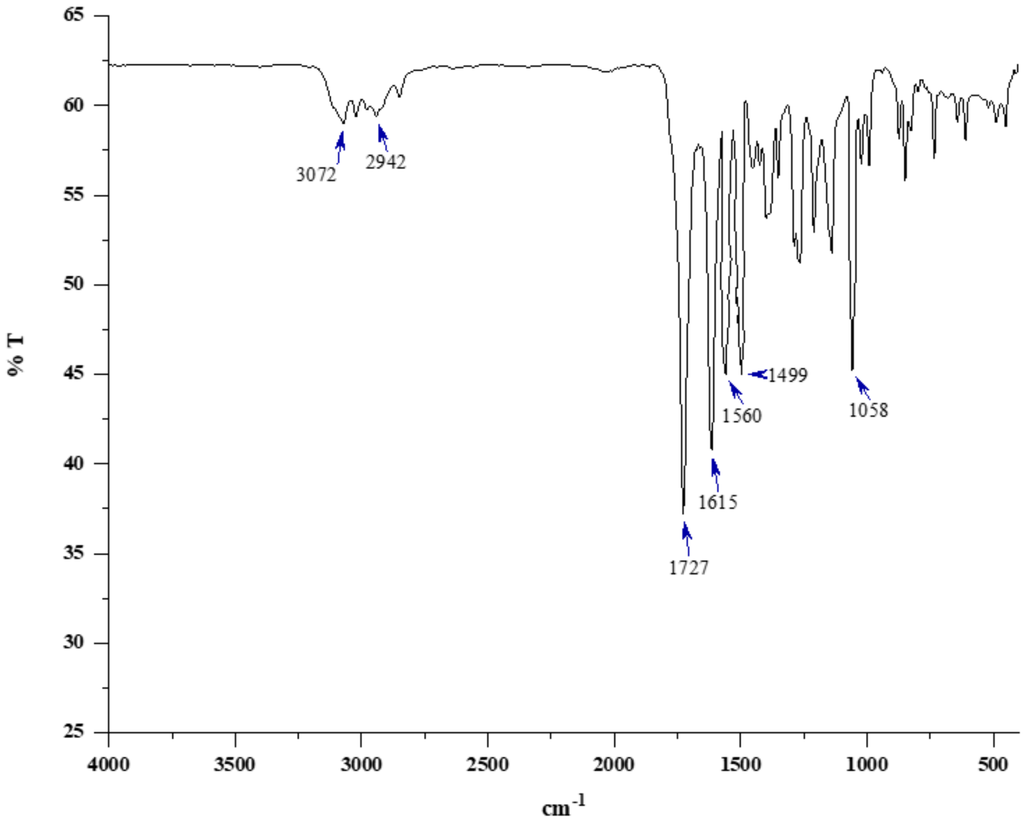

IR spectrum of 4-(chloromethyl)-7-methoxy-2*H*-chromen-2-one **(9)**

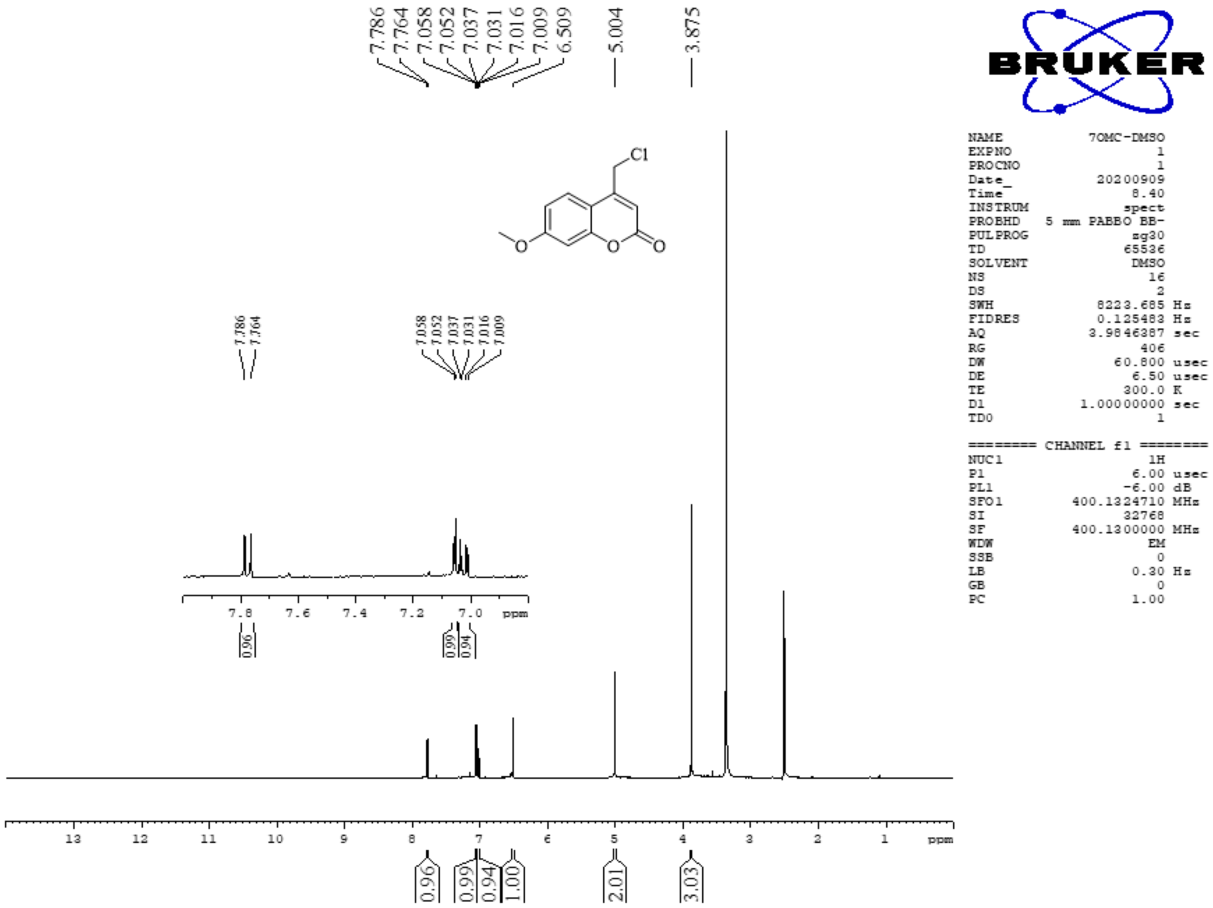

^{1}H NMR spectrum of 4-(chloromethyl)-7-methoxy-2*H*-chromen-2-one **(9)**

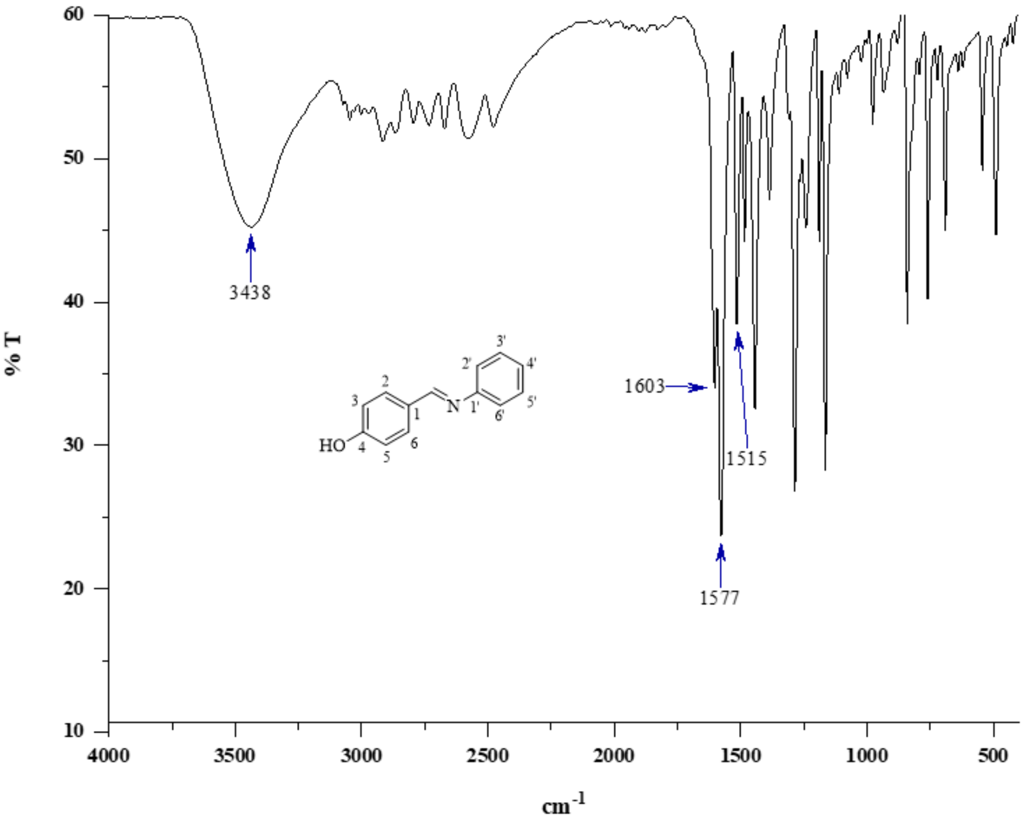

IR spectrum of (*E*)-4-((phenylimino)methyl)phenol **(12a)**

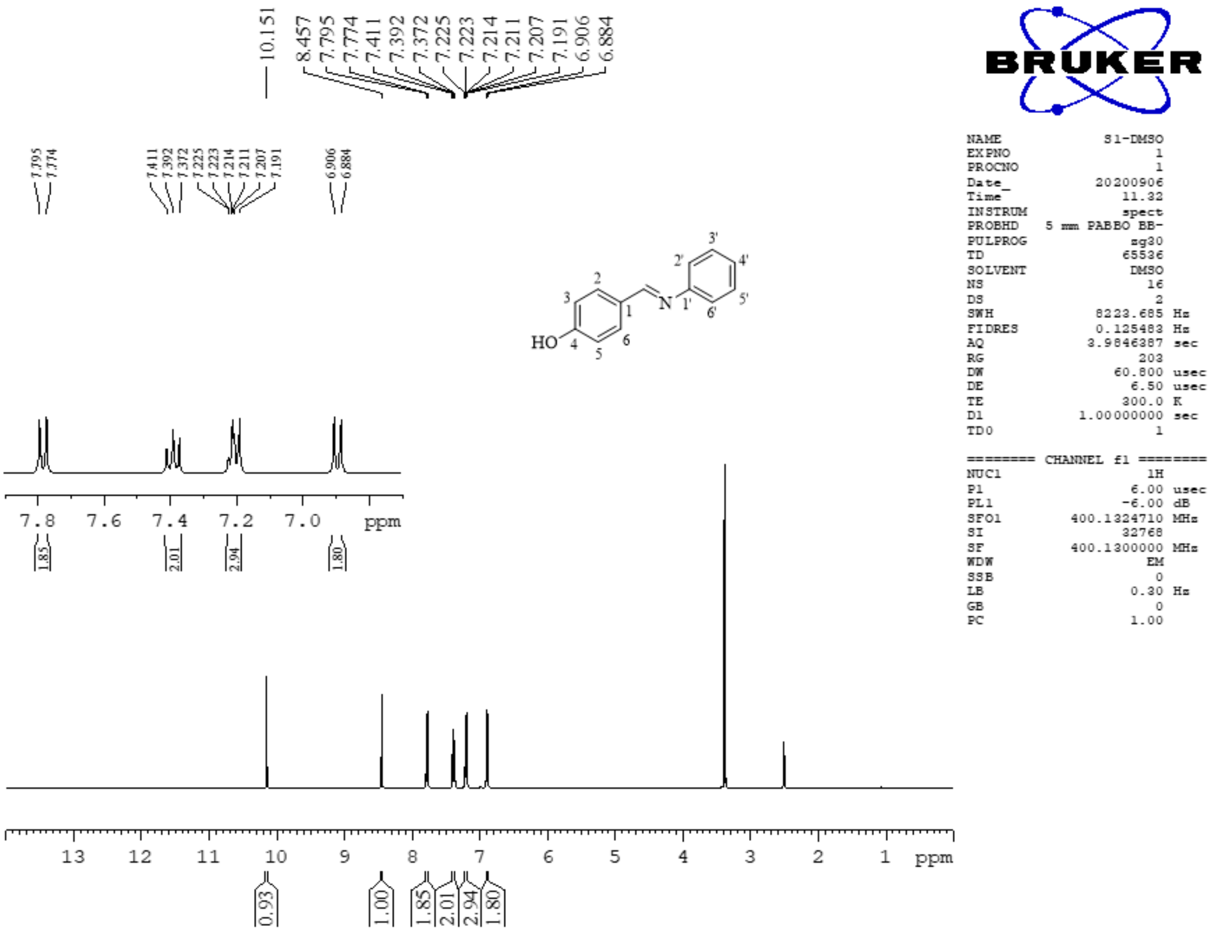

^{1}H NMR spectrum of 4-((phenylimino)methyl)phenol **(12a)**

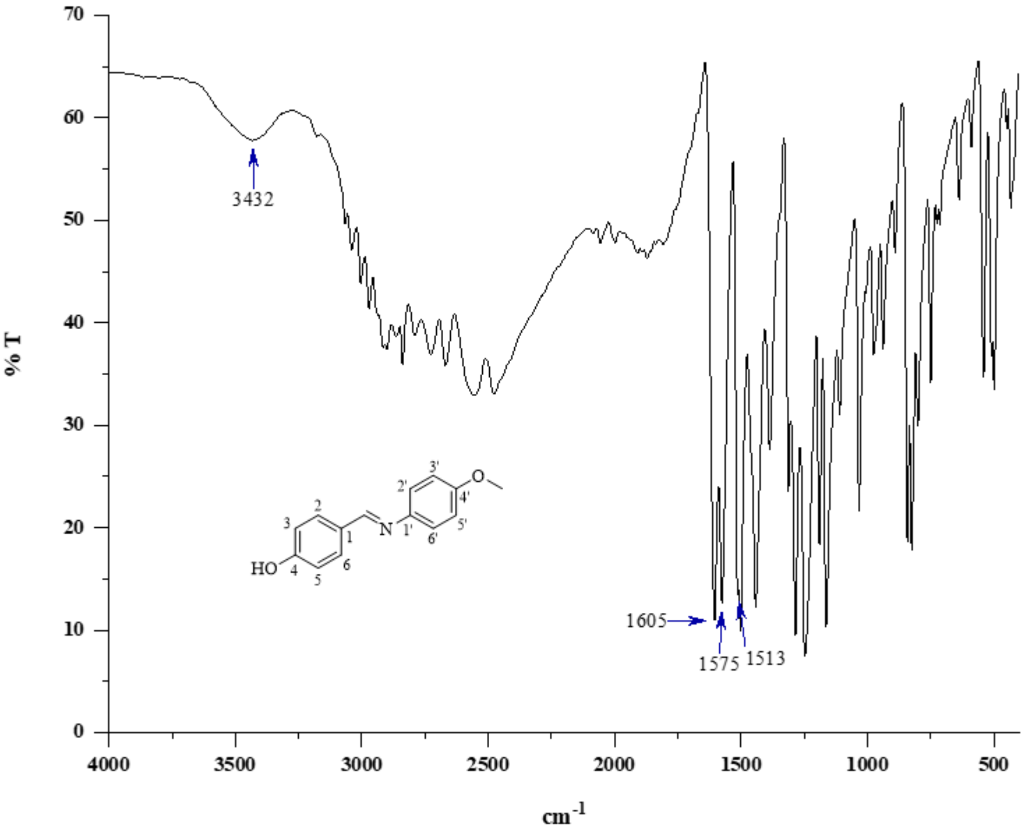

IR spectrum of (*E*)-4-(((4-methoxyphenyl)imino)methyl)phenol **(12b)**

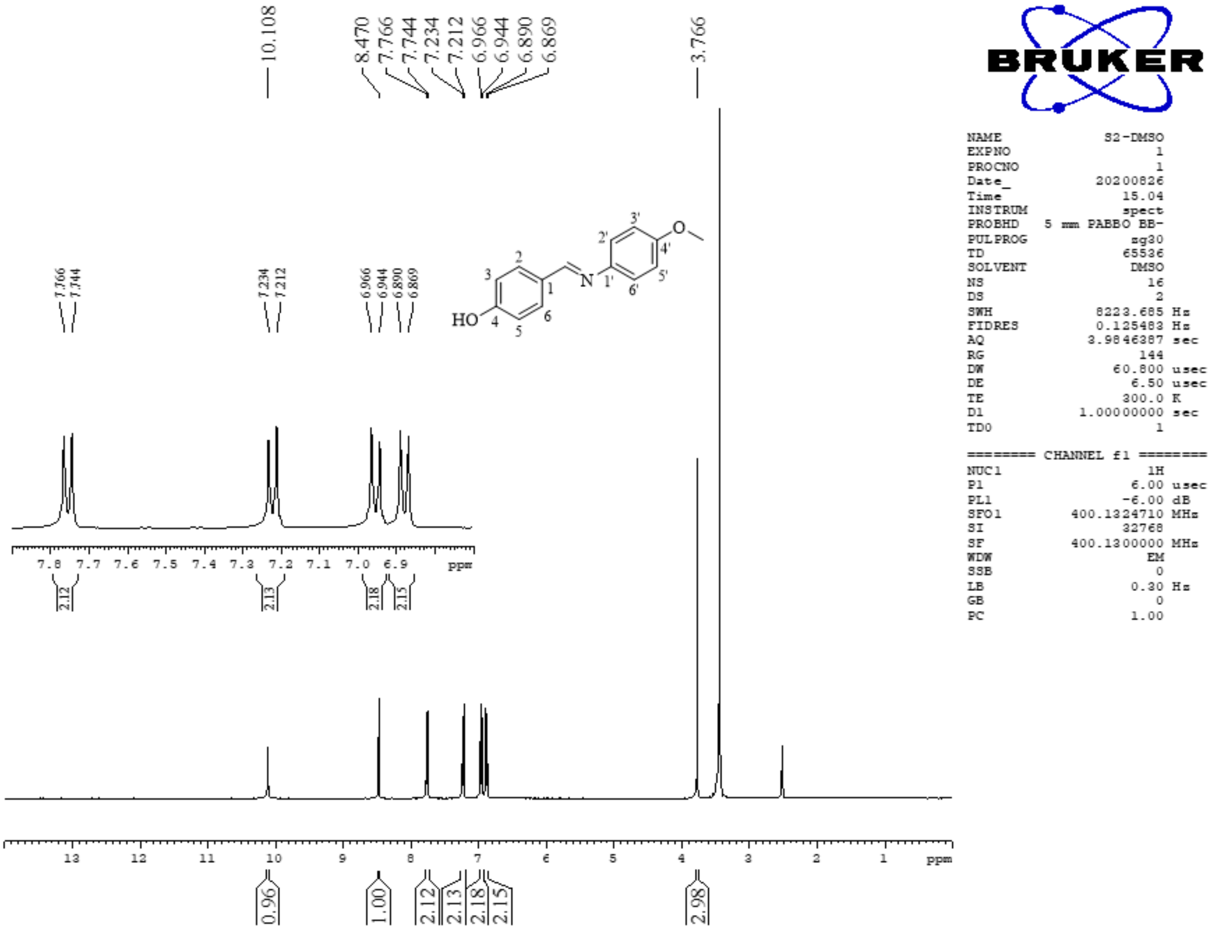

^{1}H NMR spectrum of (*E*)-4-(((4-methoxyphenyl)imino)methyl)phenol **(12b)**

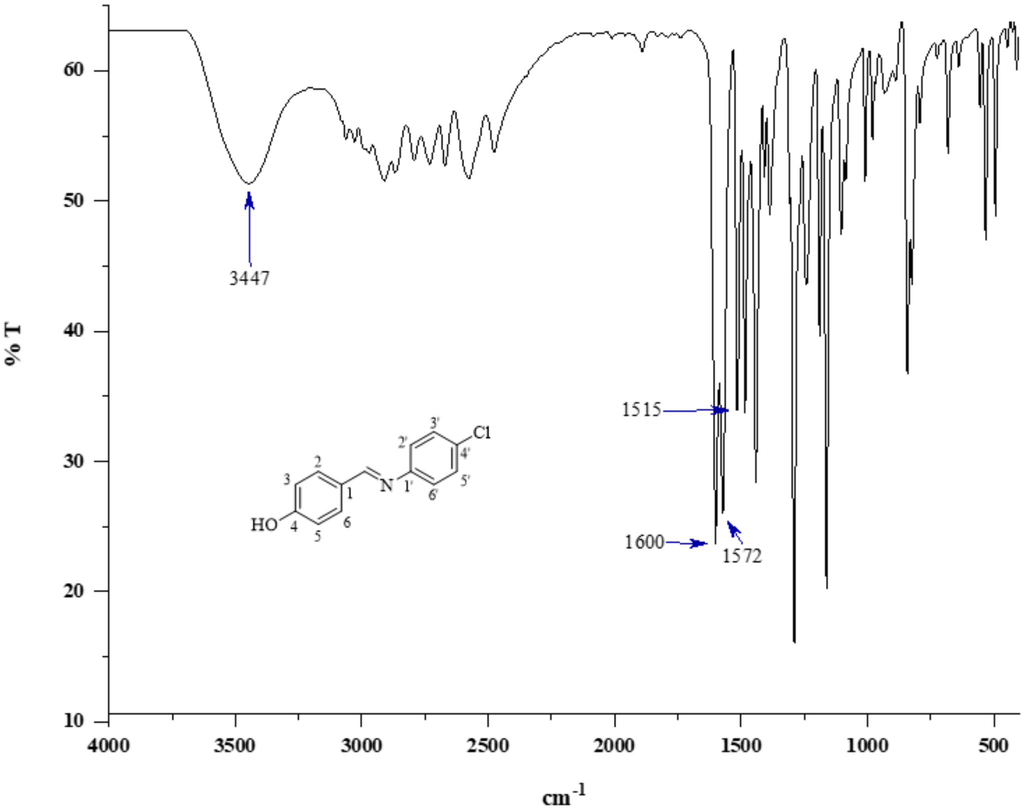

IR spectrum of (*E*)-4-(((4-chlorophenyl)imino)methyl)phenol **(12c)**

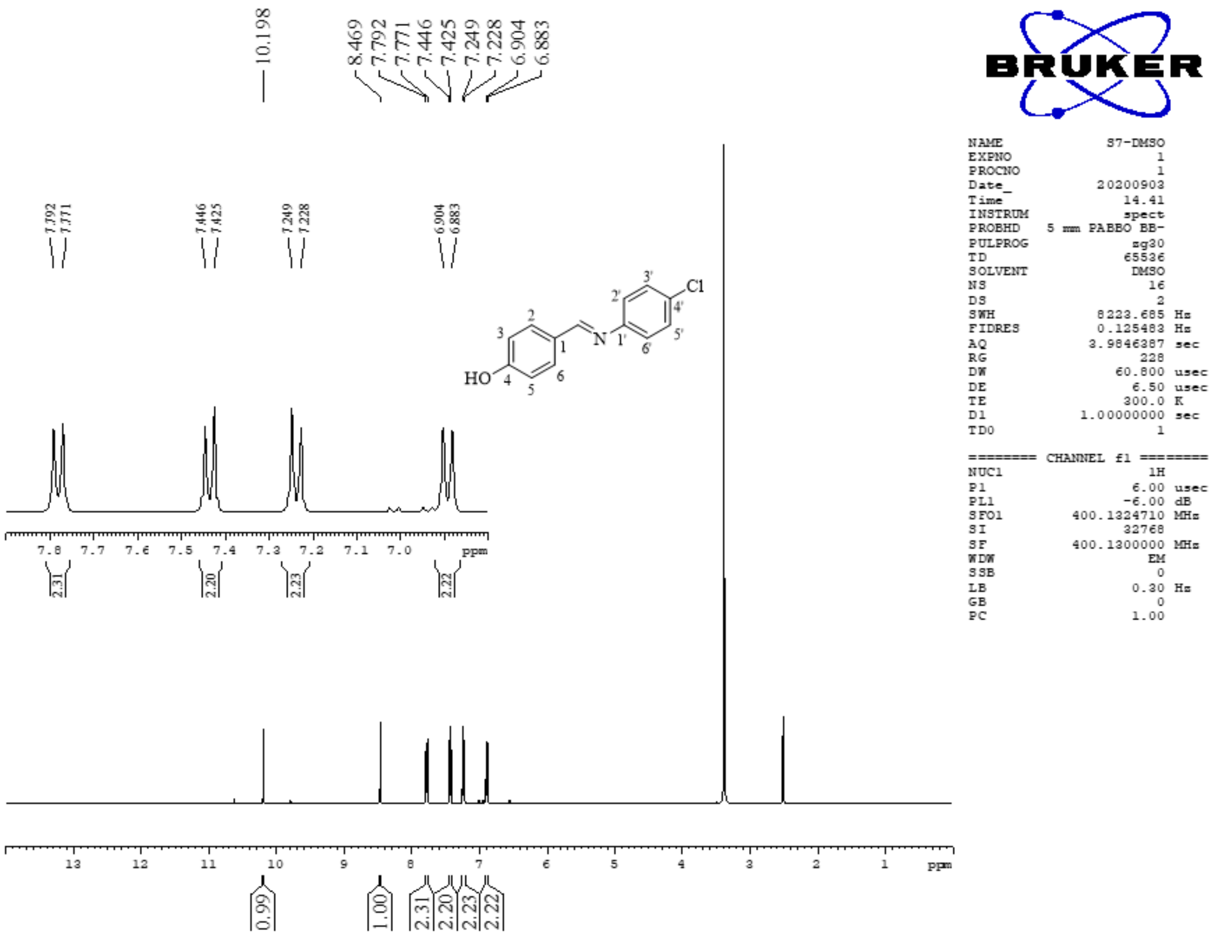

^{1}H NMR spectrum of (*E*)-4-(((4-chlorophenyl)imino)methyl)phenol **(12c)**

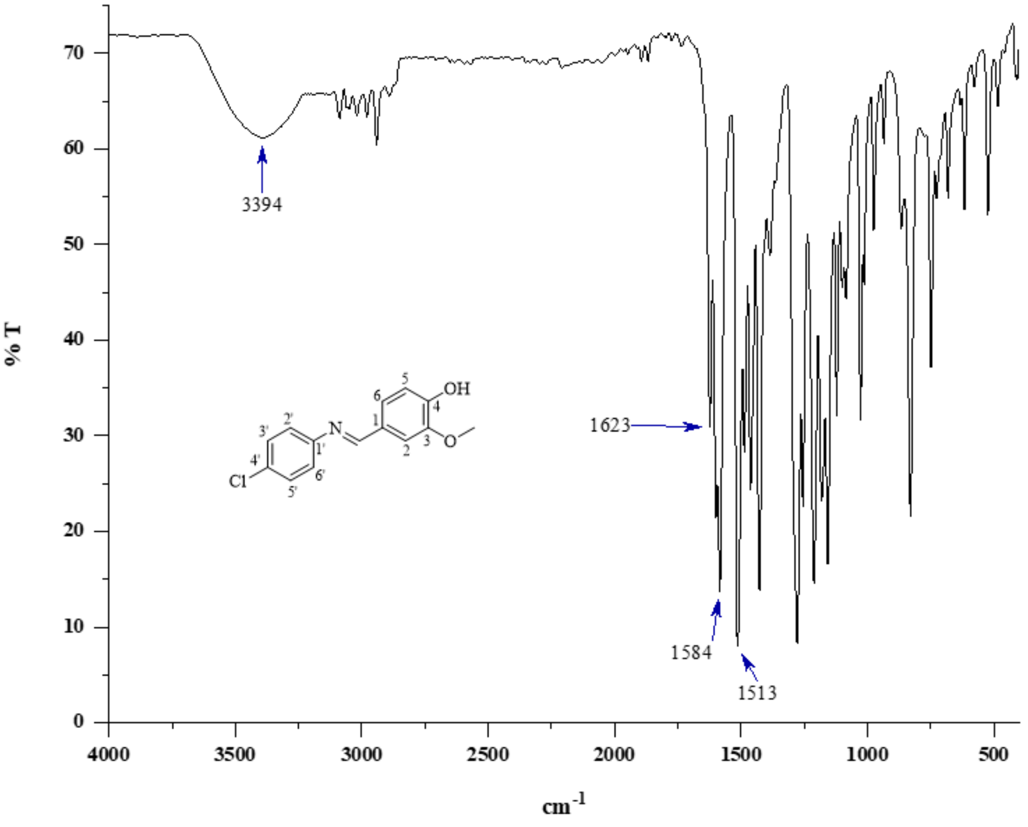

IR spectrum of (*E*)-4-(((4-chlorophenyl)imino)methyl)-2-methoxyphenol **(12d)**

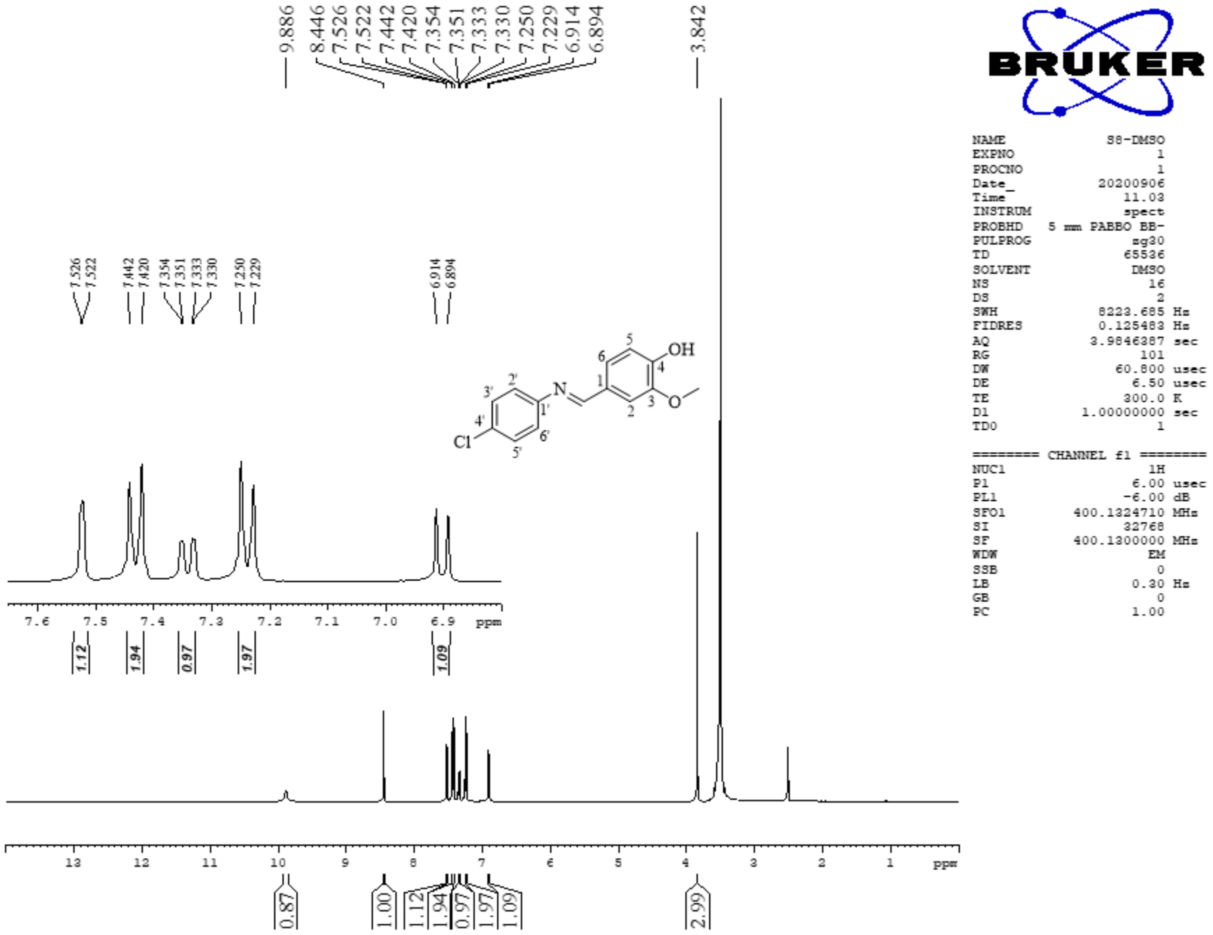

^{1}H NMR spectrum of (*E*)-4-(((4-chlorophenyl)imino)methyl)-2-methoxyphenol **(12d)**

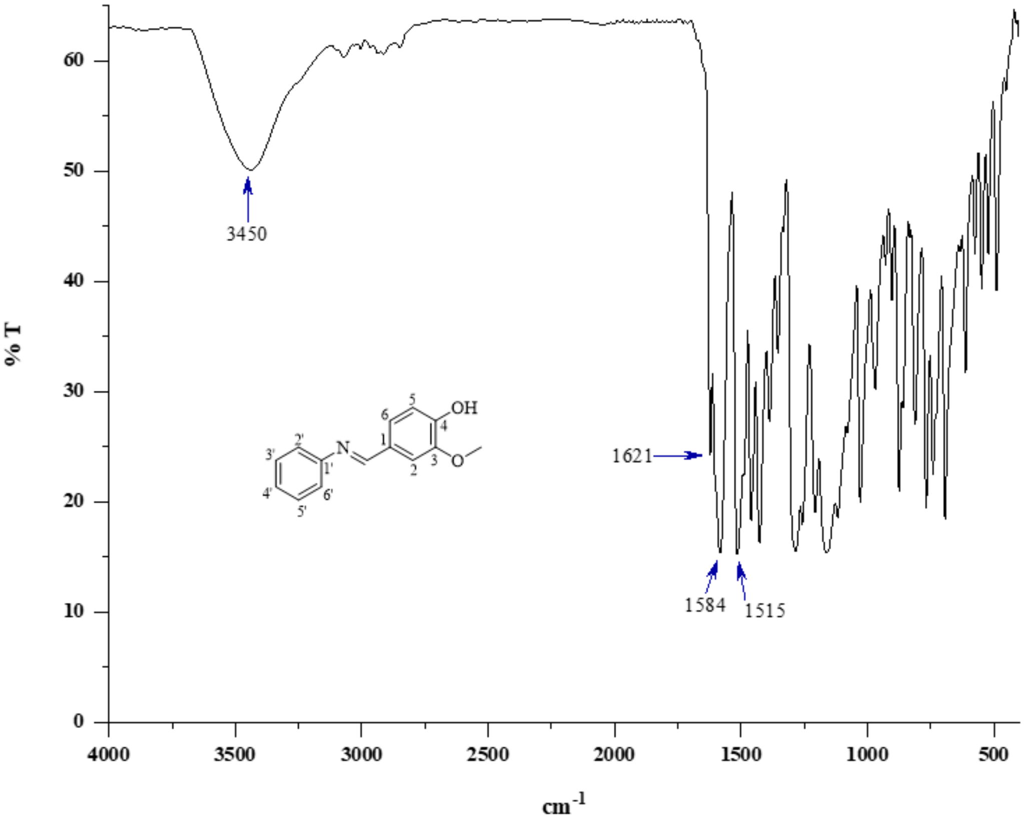

IR spectrum of (*E*)-2-methoxy-4-((phenylimino)methyl)phenol **(12e)**

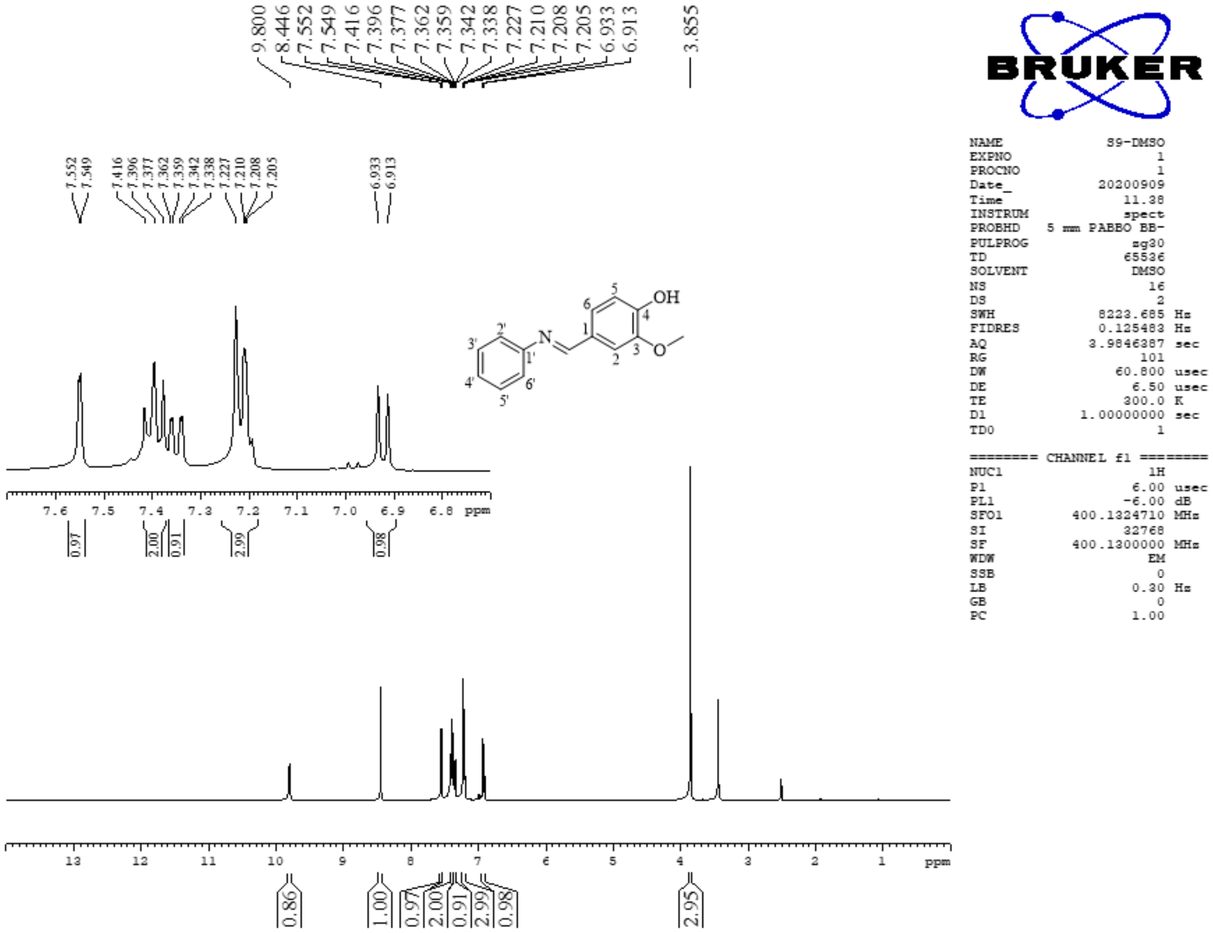

^{1}H NMR spectrum of (*E*)-2-methoxy-4-((phenylimino)methyl)phenol **(12e)**

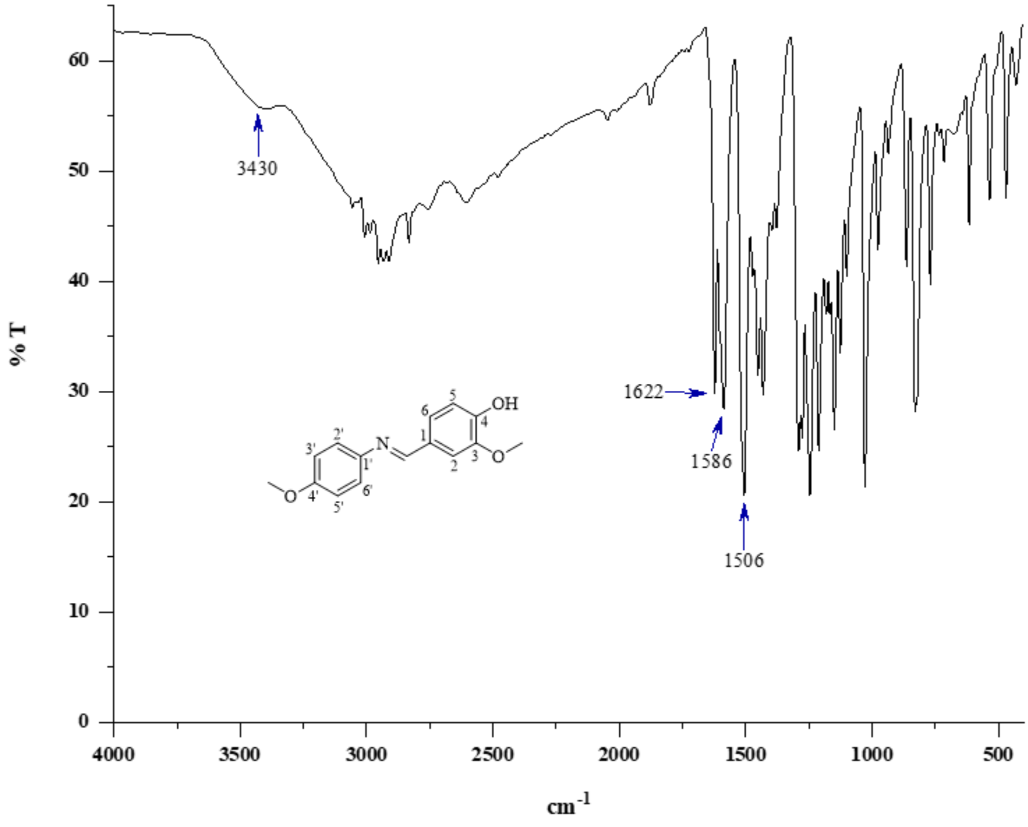

IR spectrum of (*E*)-2-methoxy-4-(((4-methoxyphenyl)imino)methyl)phenol **(12f)**

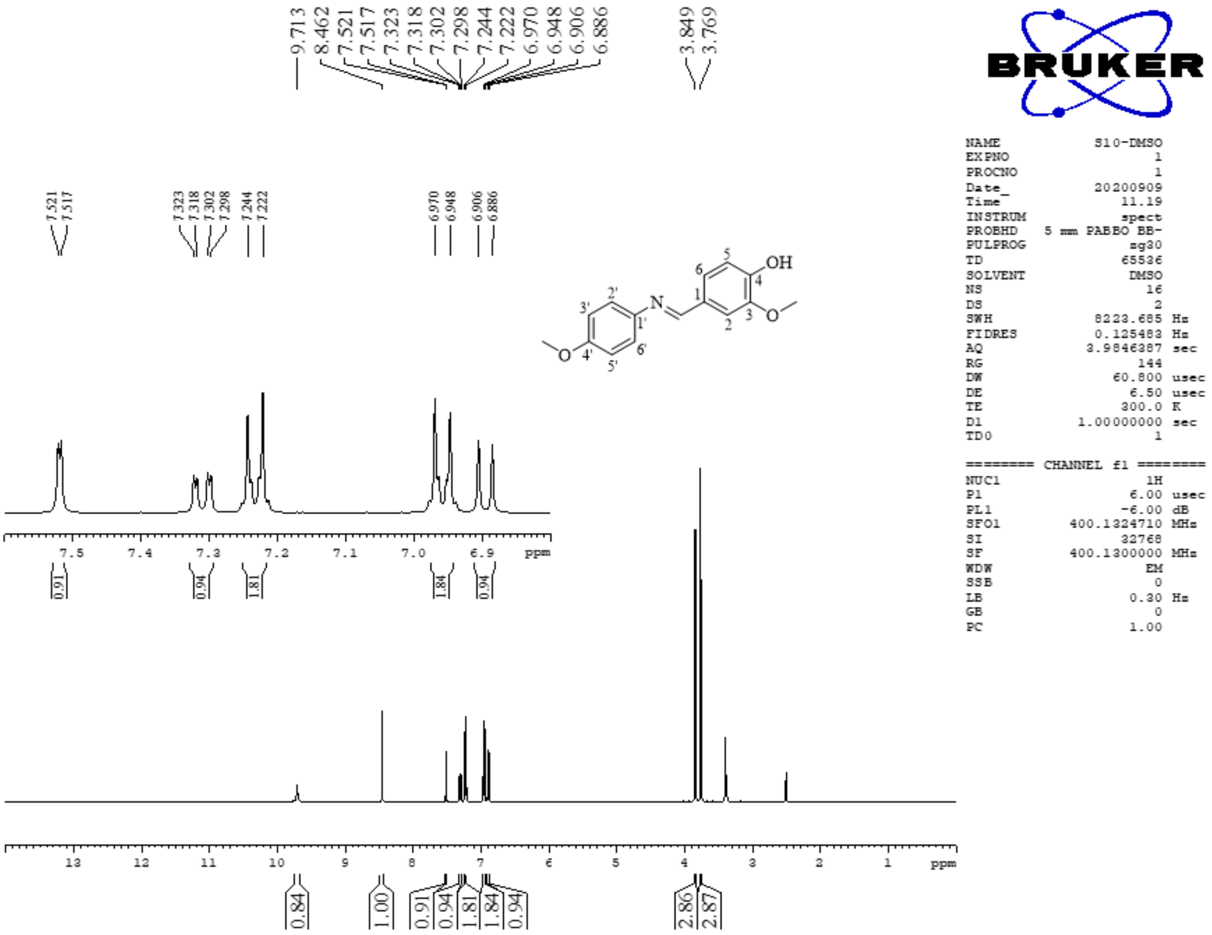

^{1}H NMR spectrum of (*E*)-2-methoxy-4-(((4-methoxyphenyl)imino)methyl)phenol **(12f)**

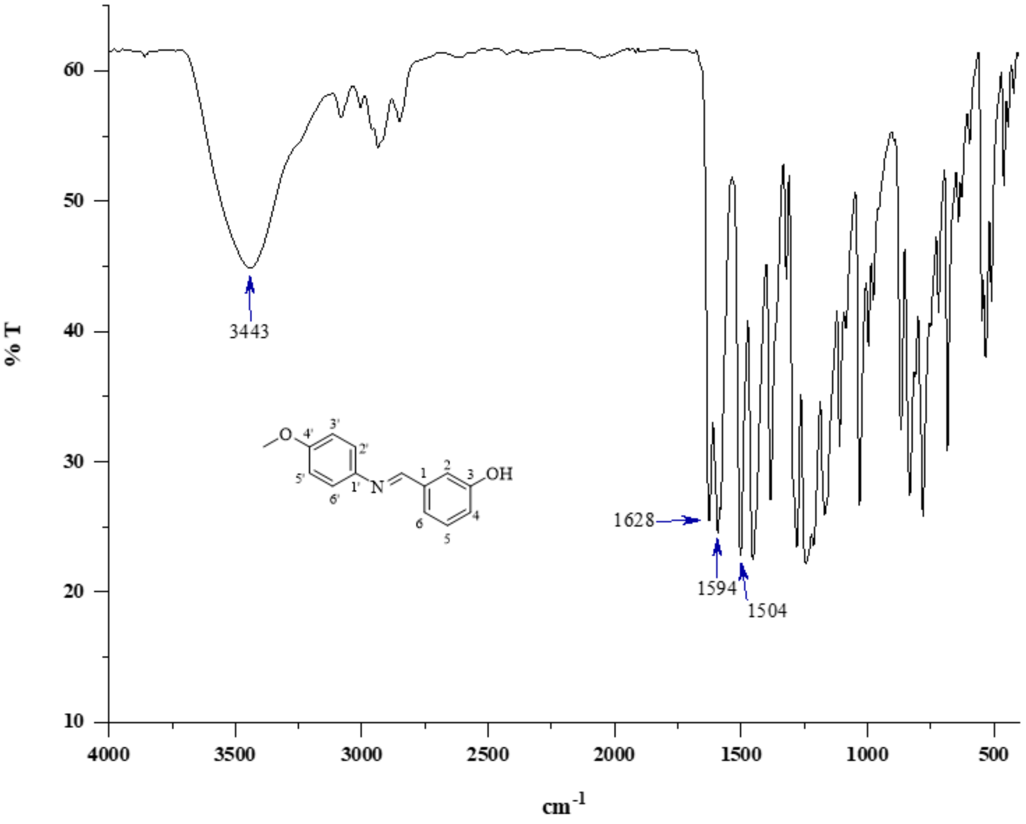

IR spectrum of (*E*)-3-(((4-methoxyphenyl)imino)methyl)phenol **(12g)**

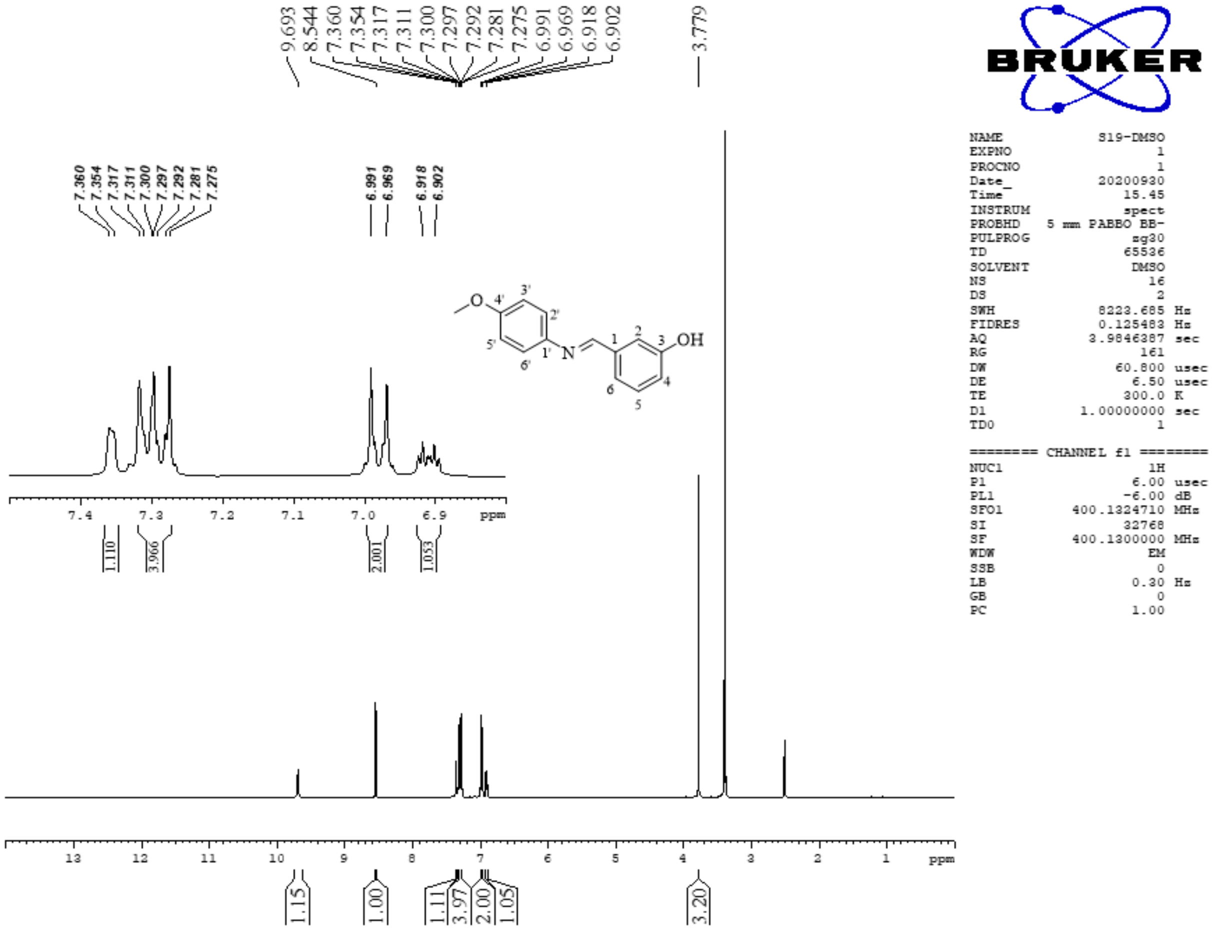

^{1}H NMR spectrum of (*E*)-3-(((4-methoxyphenyl)imino)methyl)phenol **(12g)**

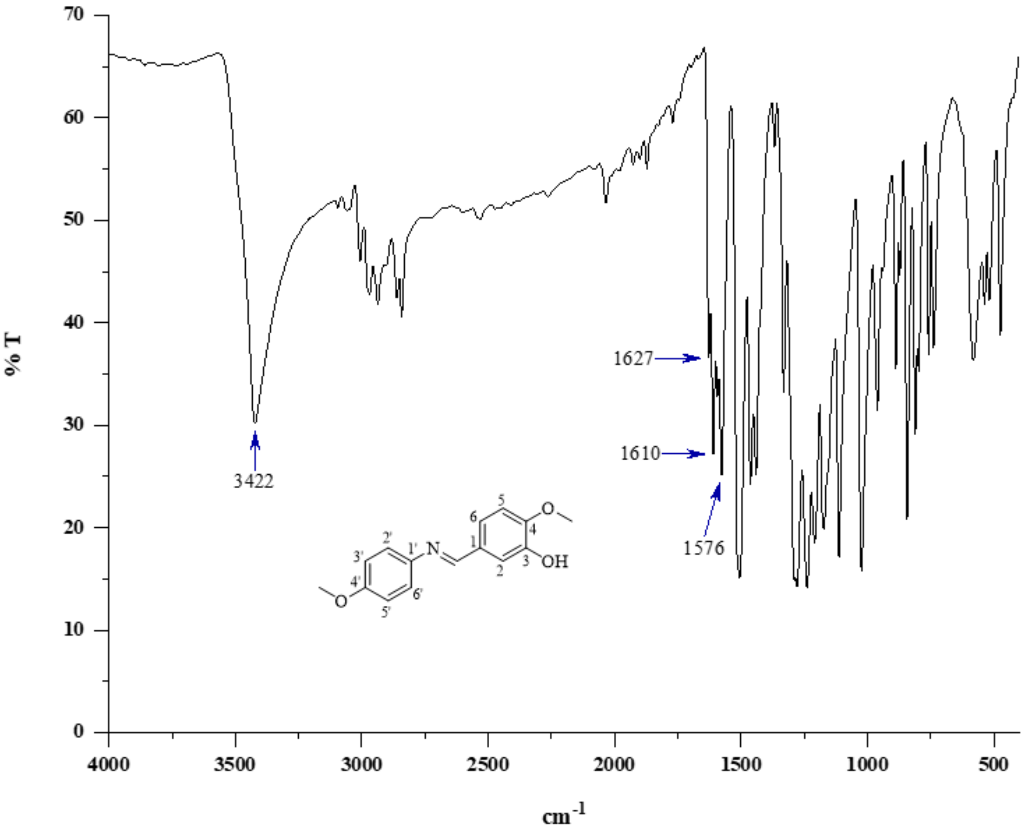

IR spectrum of (*E*)-2-methoxy-5-(((4-methoxyphenyl)imino)methyl) phenol **(12h)**

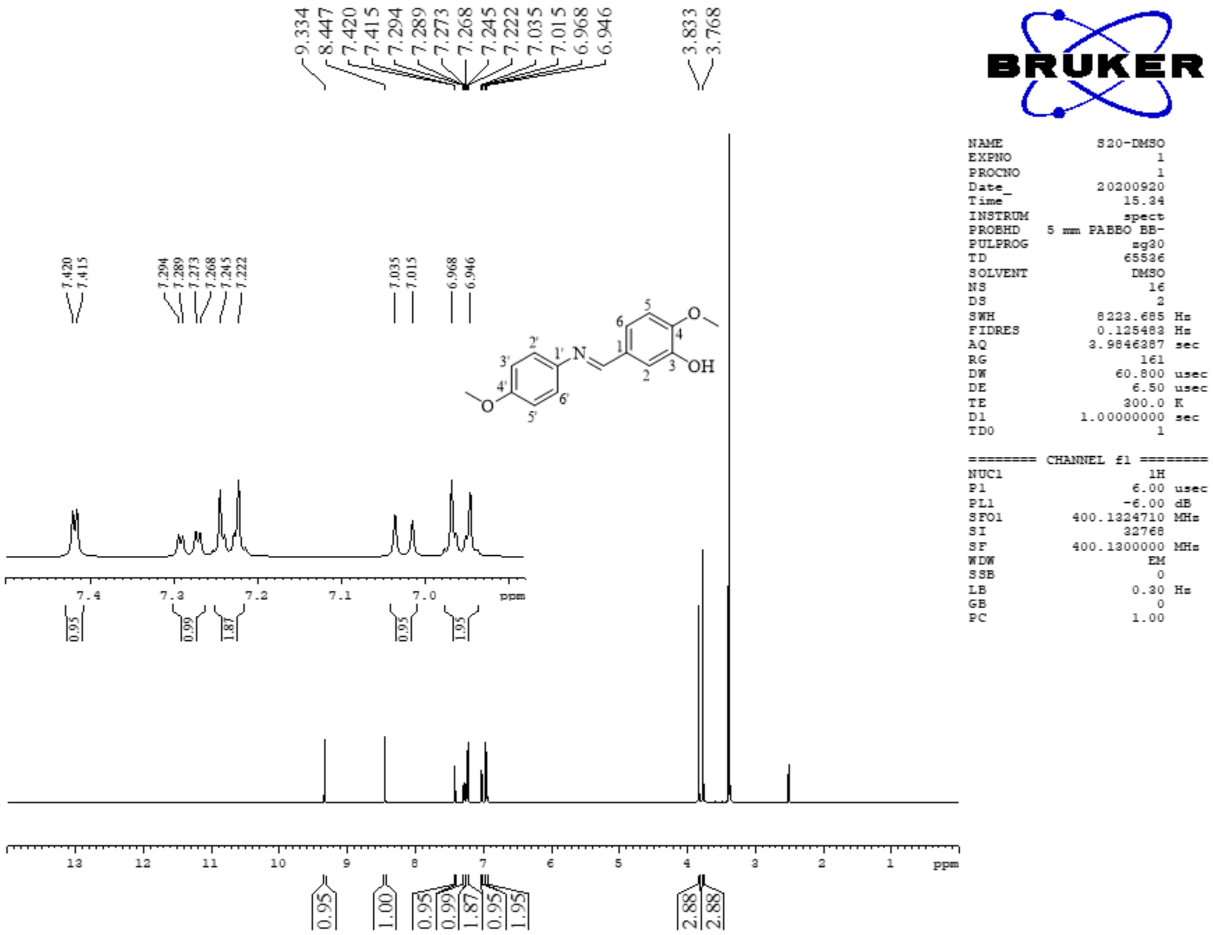

^{1}H NMR spectrum of (*E*)-2-methoxy-5-(((4-methoxyphenyl)imino)methyl) phenol **(12h)**

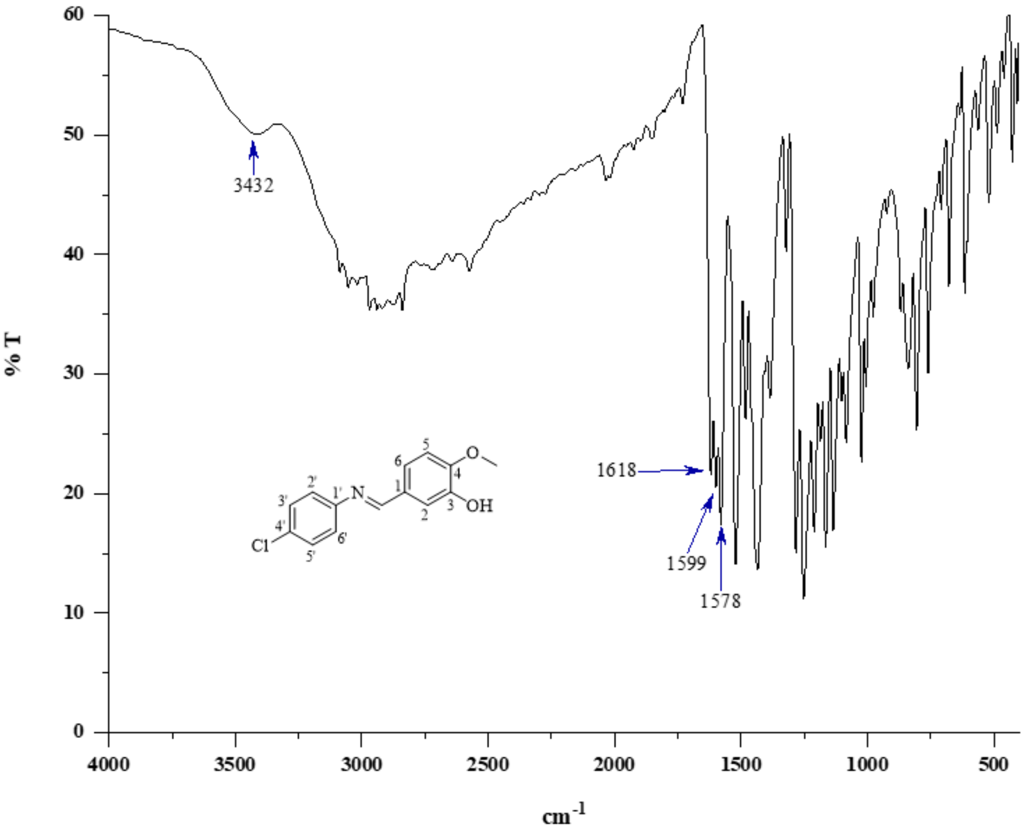

IR spectrum of (*E*)-5-(((4-chlorophenyl)imino)methyl)-2-methoxyphenol **(12i)**

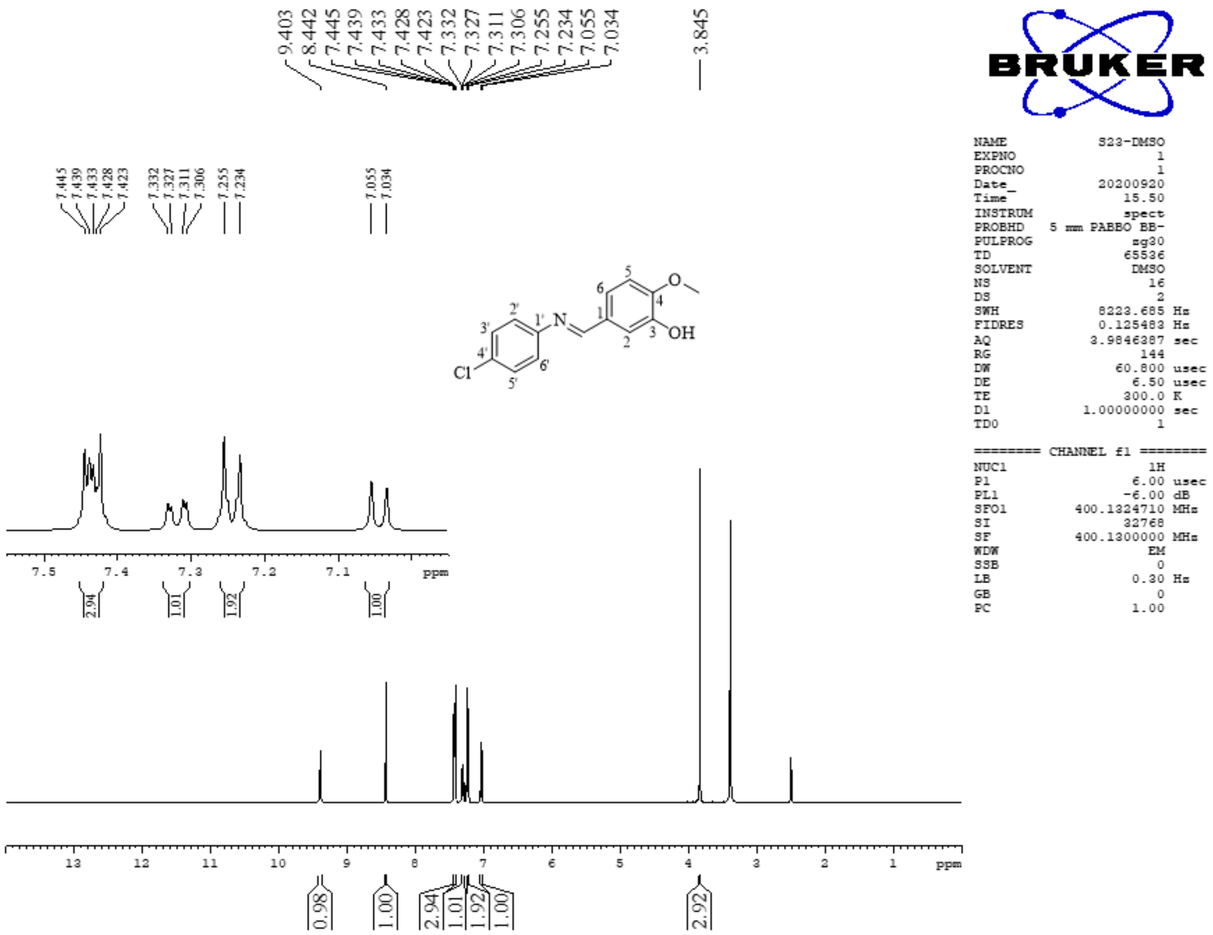

^{1}H NMR spectrum of (*E*)-5-(((4-chlorophenyl)imino)methyl)-2-methoxyphenol **(12i)**

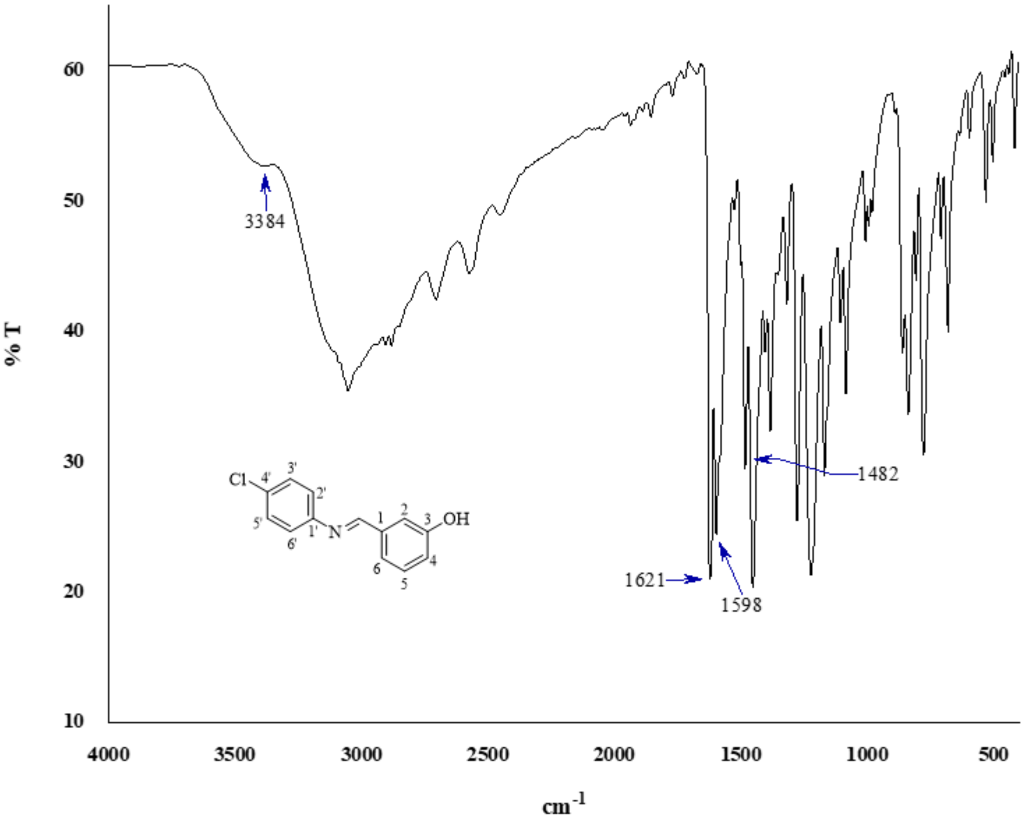

IR spectrum of (*E*)-3-(((4-chlorophenyl)imino)methyl)phenol **(12j)**

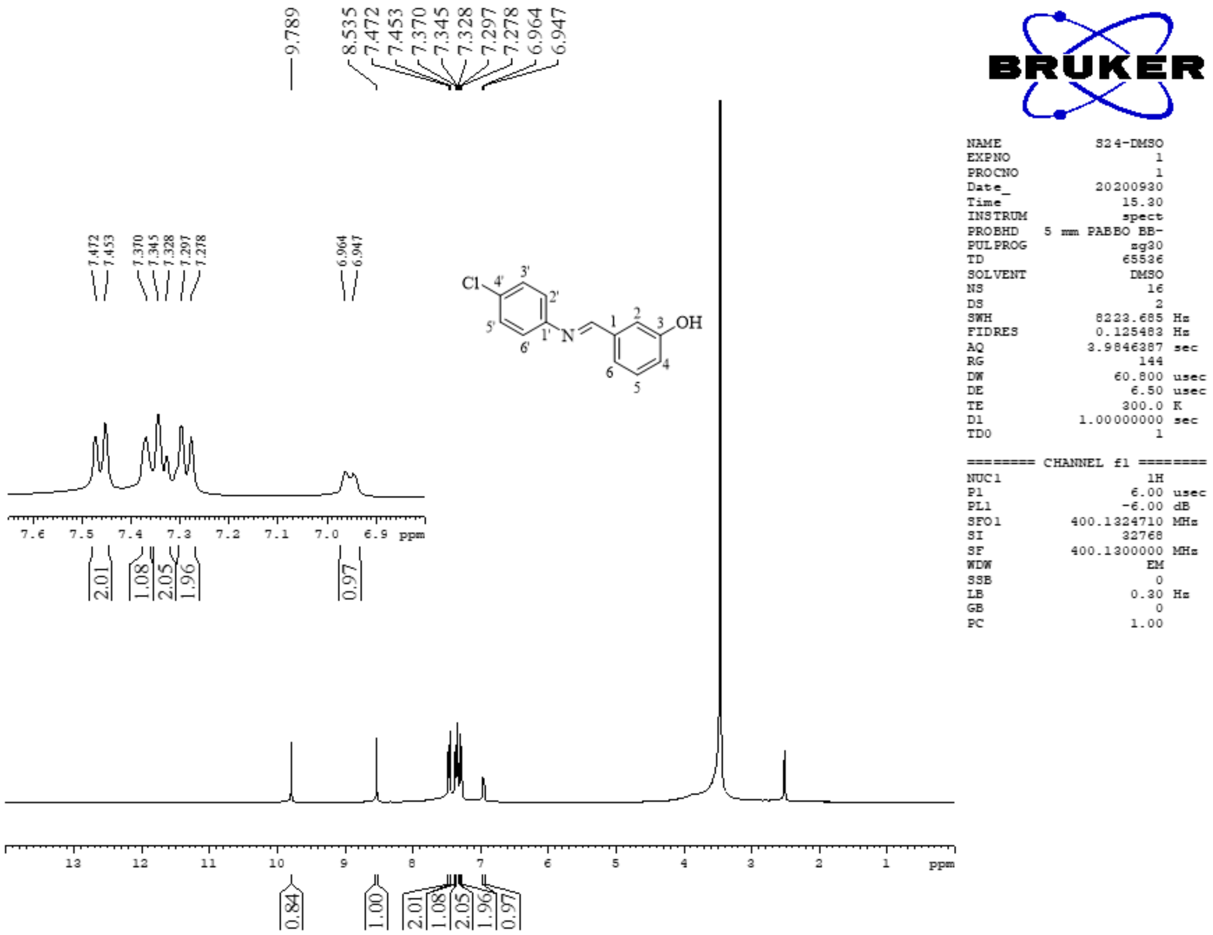

^{1}H NMR spectrum of (*E*)-3-(((4-chlorophenyl)imino)methyl)phenol **(12j)**

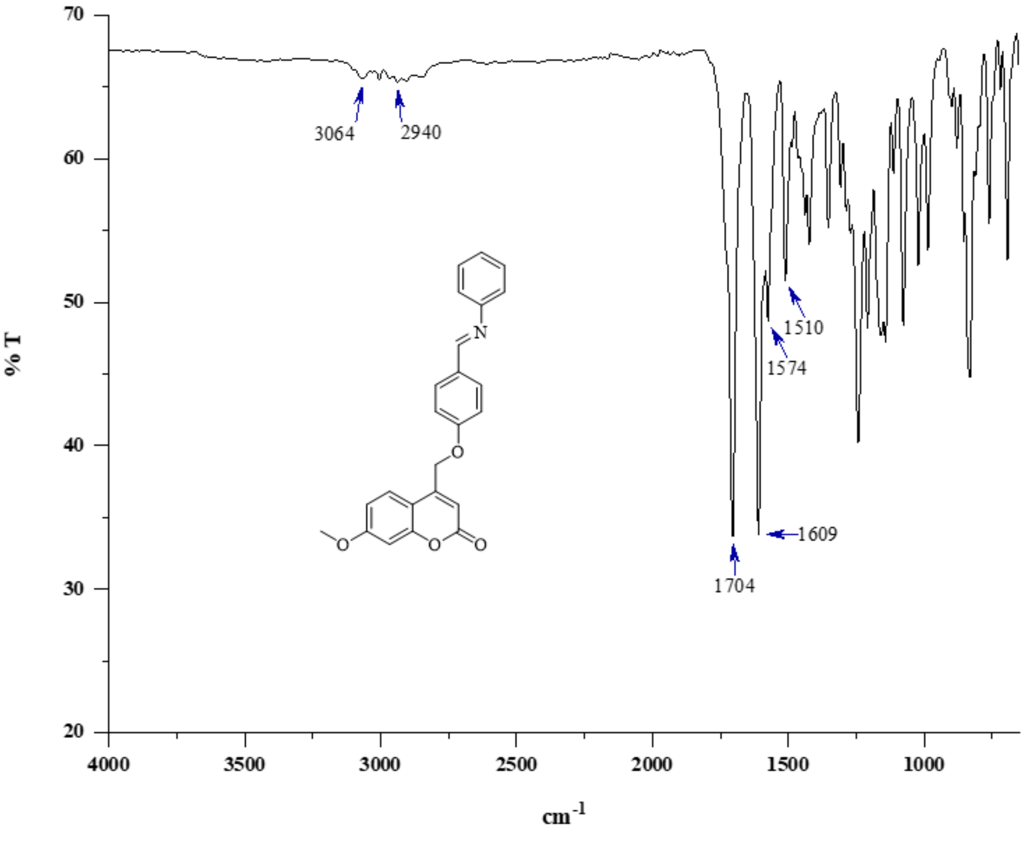

IR spectrum of (*E*)-7-methoxy-4-((4-((phenylimino)methyl)phenoxy)methyl) -2*H*-chromen-2-one **(13a)**

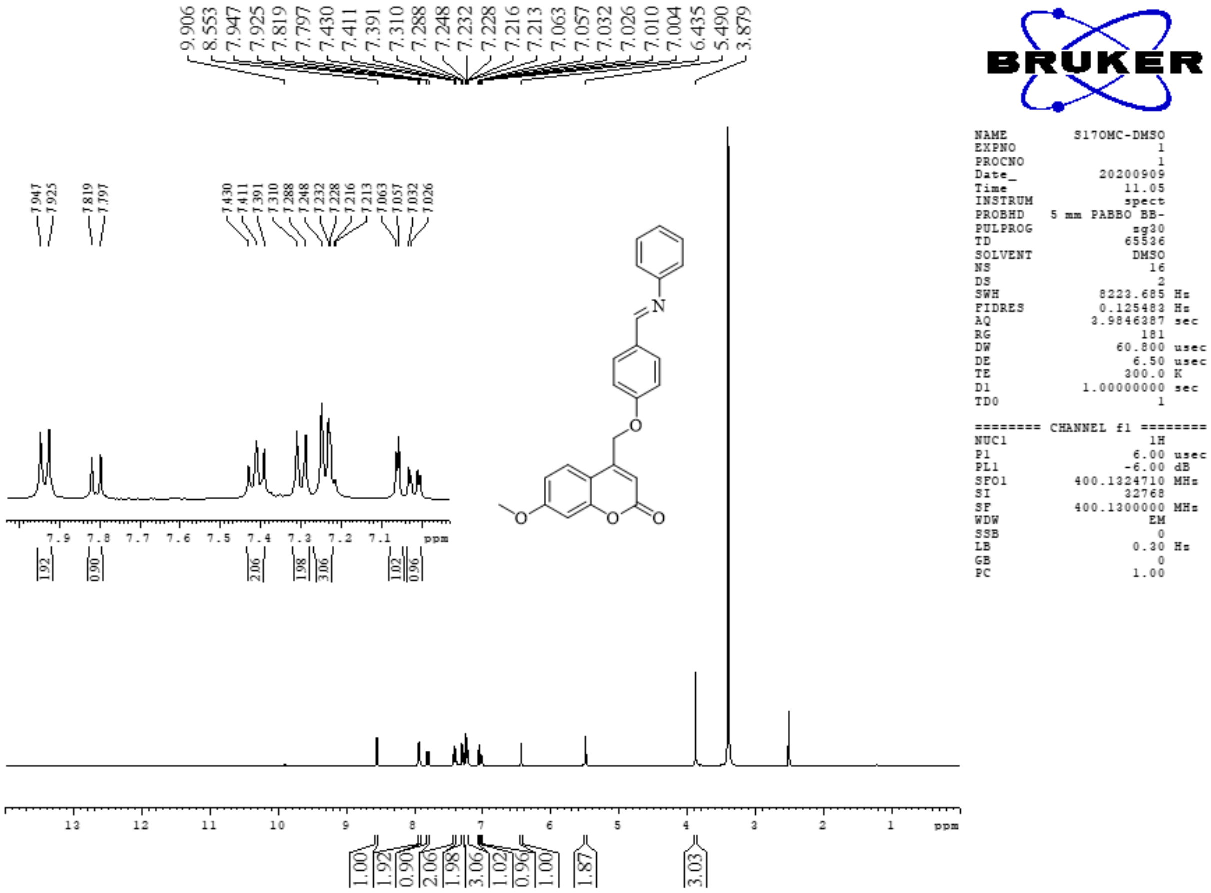

^{1}H NMR spectrum of (*E*)-7-methoxy-4-((4-((phenylimino)methyl)phenoxy)methyl) -2*H*-chromen-2-one **(13a)**

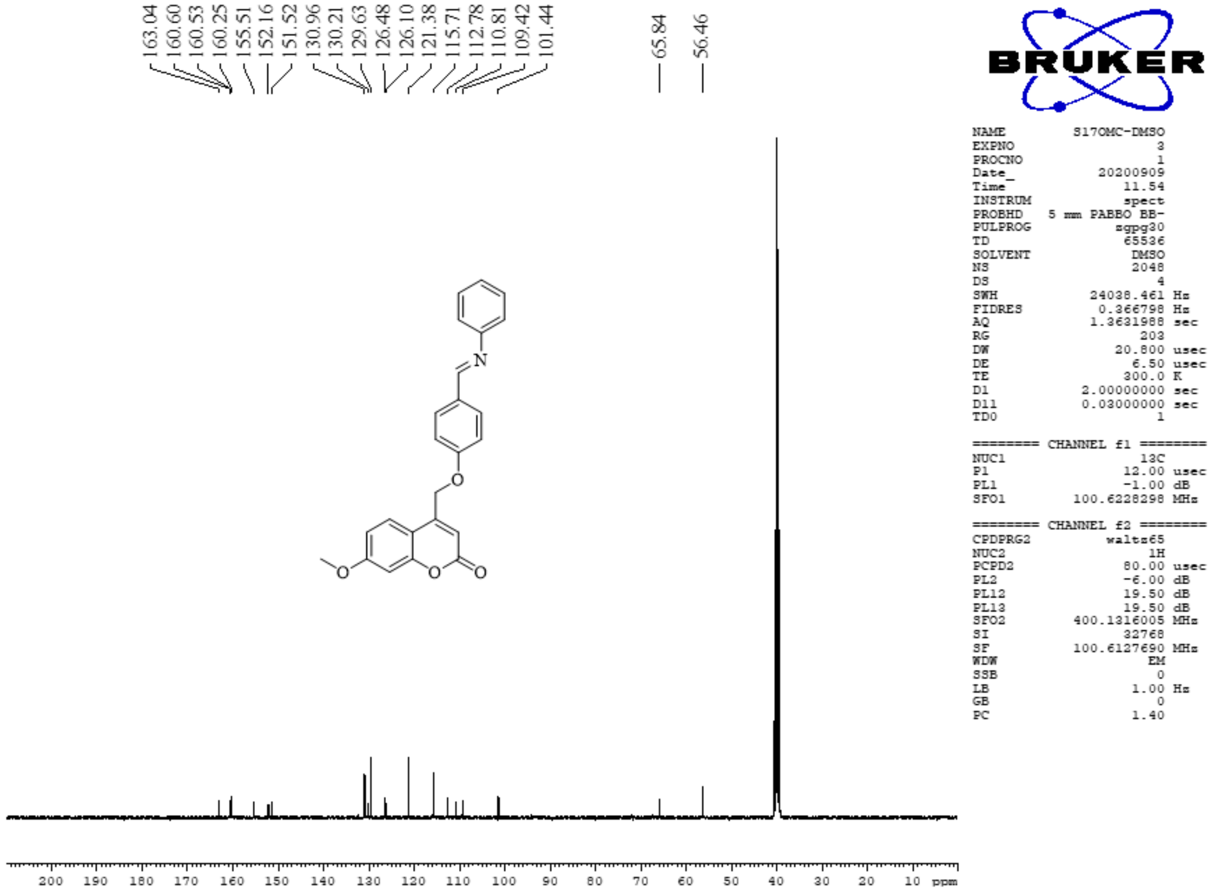

^{13}C NMR spectrum of (*E*)-7-methoxy-4-((4-((phenylimino)methyl)phenoxy)methyl) -2*H*-chromen-2-one **(13a)**

Compound Table

Compound Label	RT	Mass	Abund	Formula	Tgt Mass	Diff (ppm)
Cpd 1: C24 H19 N O4	0.232	385.1312	113	C24 H19 N O4	385.1314	-0.56

Compound Label	*m/z*	RT	Algorithm	Mass
Cpd 1: C24 H19 N O4	390.113	0.232	Find By Formula	385.1312

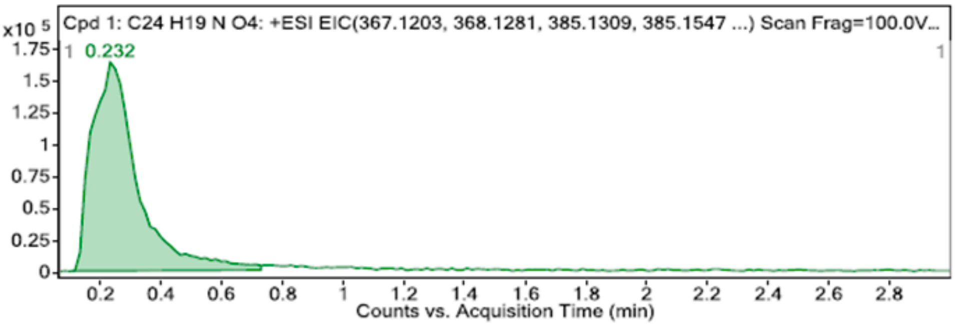

MS Zoomed Spectrum

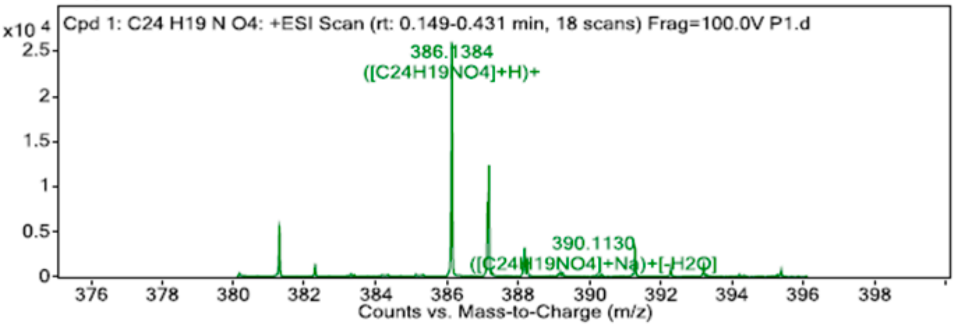

HRMS of (*E*)-7-methoxy-4-((4-((phenylimino)methyl)phenoxy)methyl) -2*H*-chromen-2-one **(13a)**

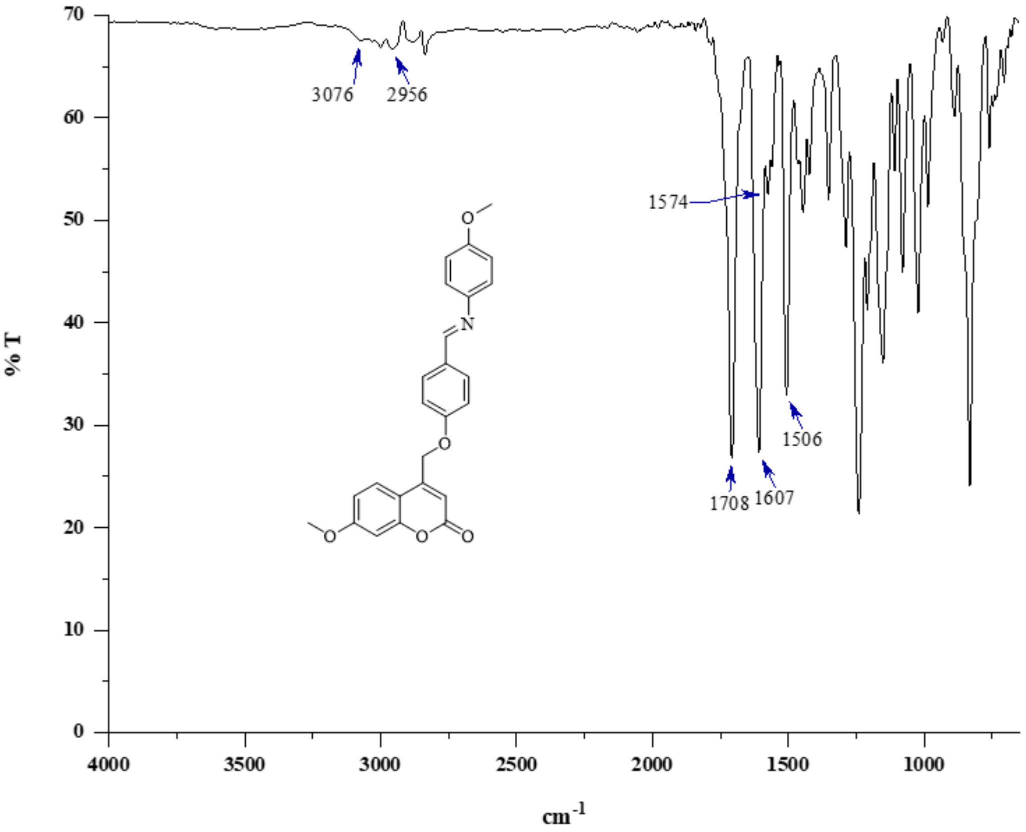

IR spectrum of (*E*)-7-methoxy-4-((4-(((4-methoxyphenyl)imino)methyl) phenoxy)methyl)-2*H*-chromen-2-one **(13b)**

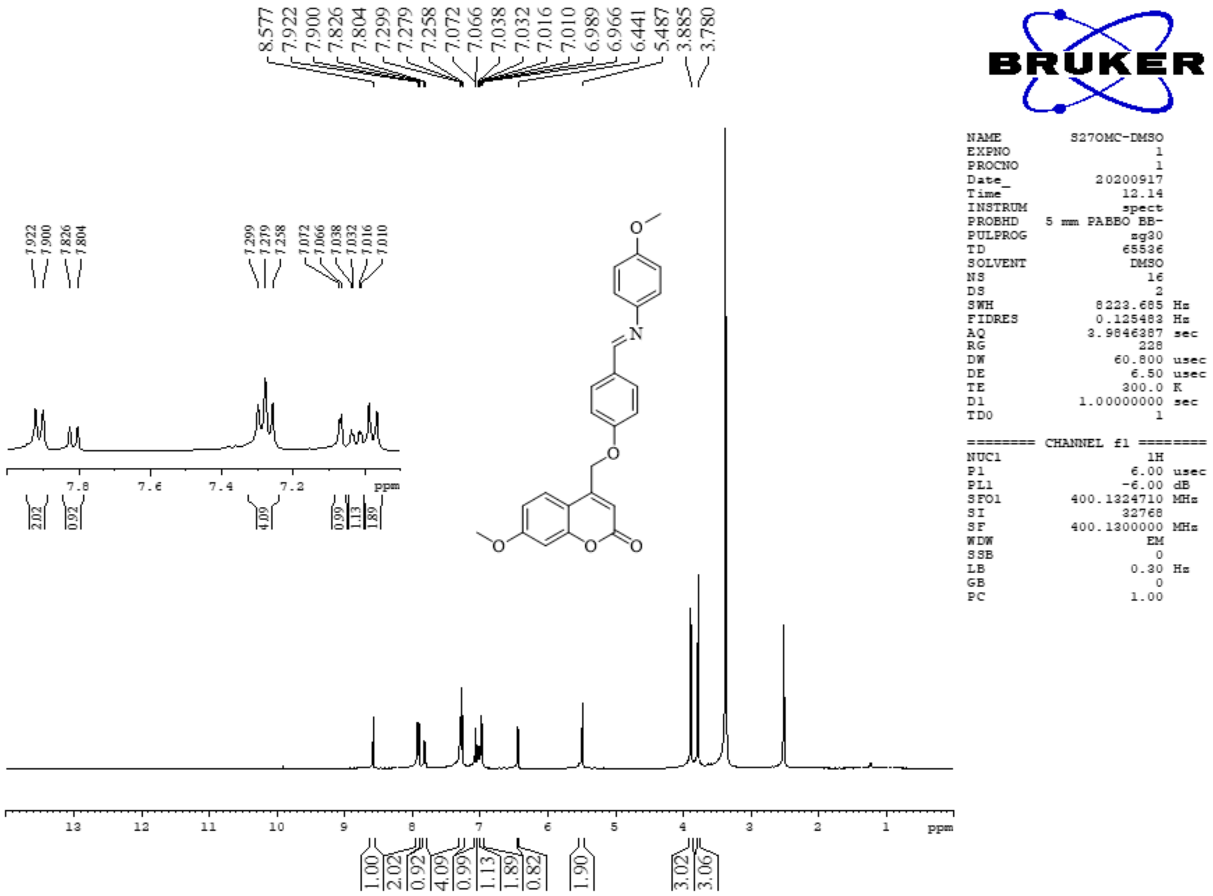

^{1}H NMR spectrum of (*E*)-7-methoxy-4-((4-(((4-methoxyphenyl)imino)methyl) phenoxy)methyl)-2*H*-chromen-2-one **(13b)**

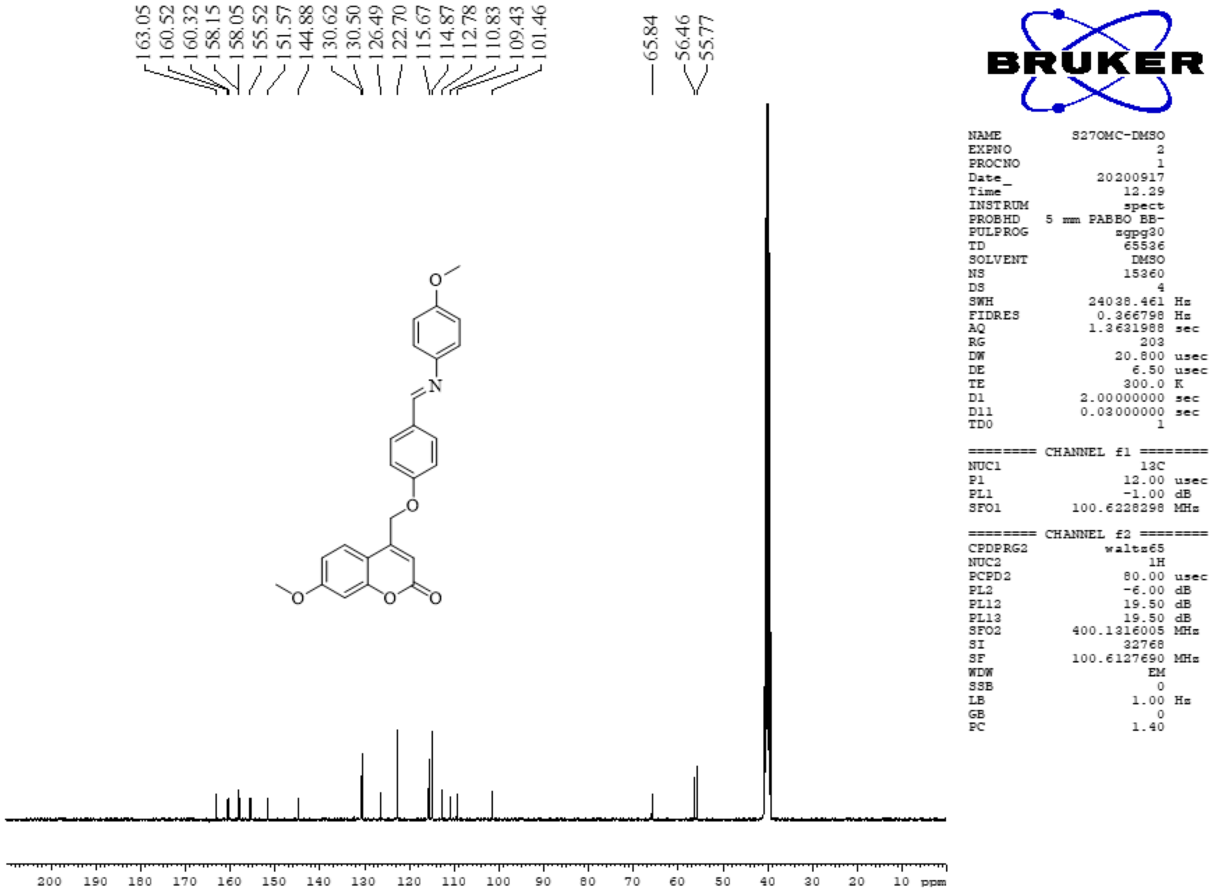

^{13}C NMR spectrum of (*E*)-7-methoxy-4-((4-(((4-methoxyphenyl)imino)methyl) phenoxy)methyl)-2*H*-chromen-2-one **(13b)**

Compound Table

Compound Label	RT	Mass	Abund	Formula	Tgt Mass	Diff (ppm)
Cpd 1: C25 H21 N O5	0.198	415.1424	432183	C25 H21 N O5	415.142	1.13

Compound Label	*m/z*	RT	Algorithm	Mass
Cpd 1: C25 H21 N O5	416.1498	0.198	Find By Formula	415.1424

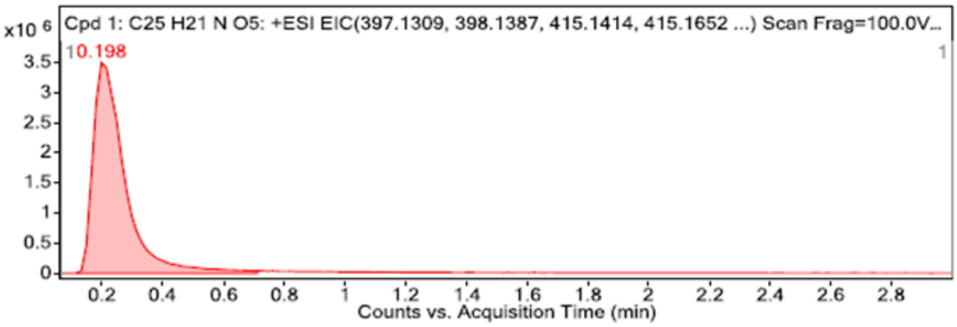

MS Zoomed Spectrum

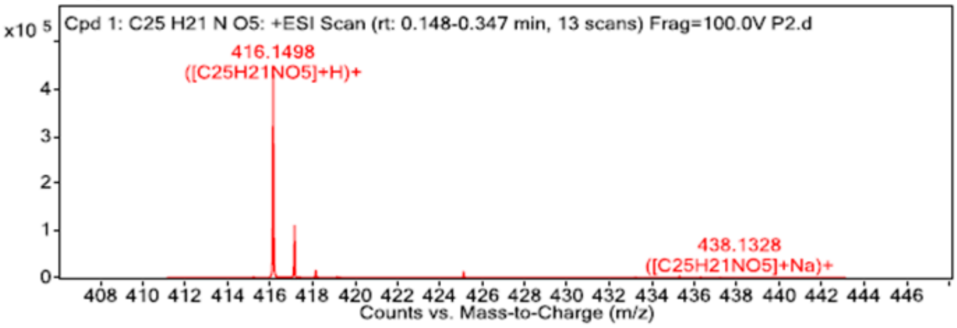

HRMS of (*E*)-7-methoxy-4-((4-(((4-methoxyphenyl)imino)methyl) phenoxy)methyl)-2*H*-chromen-2-one **(13b)**

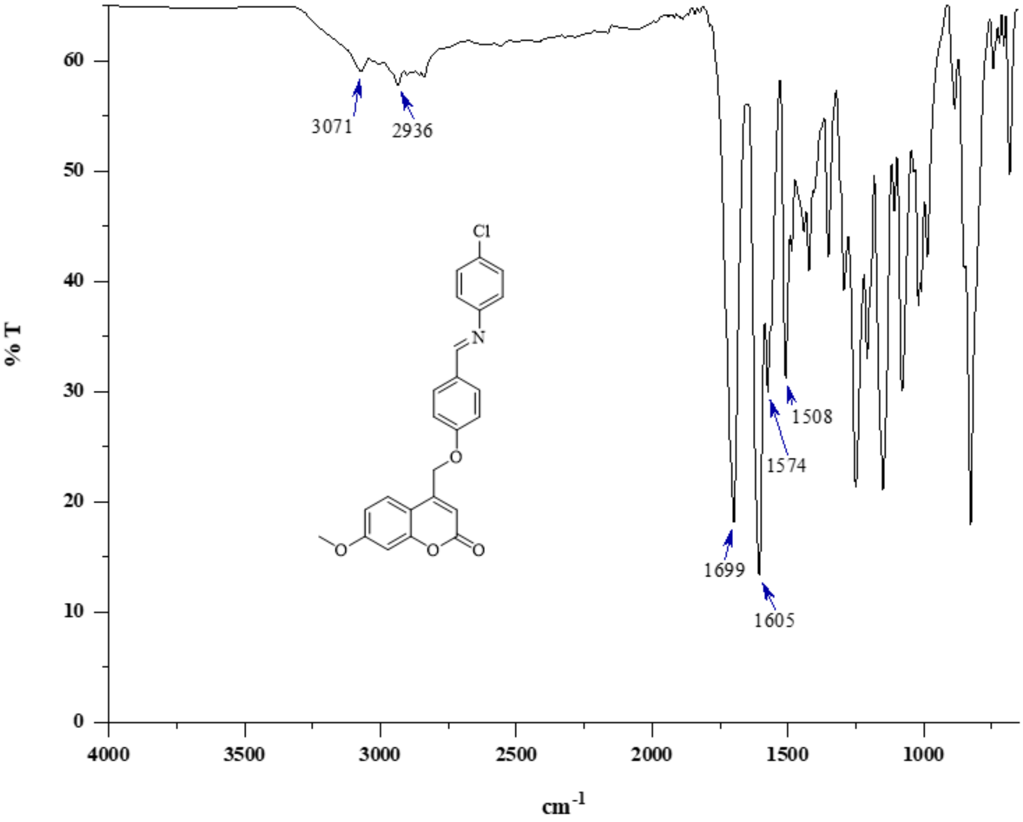

IR spectrum of (*E*)-4-((4-(((4-chlorophenyl)imino)methyl)phenoxy)methyl)-7-methoxy-2*H*-chromen-2-one **(13c)**

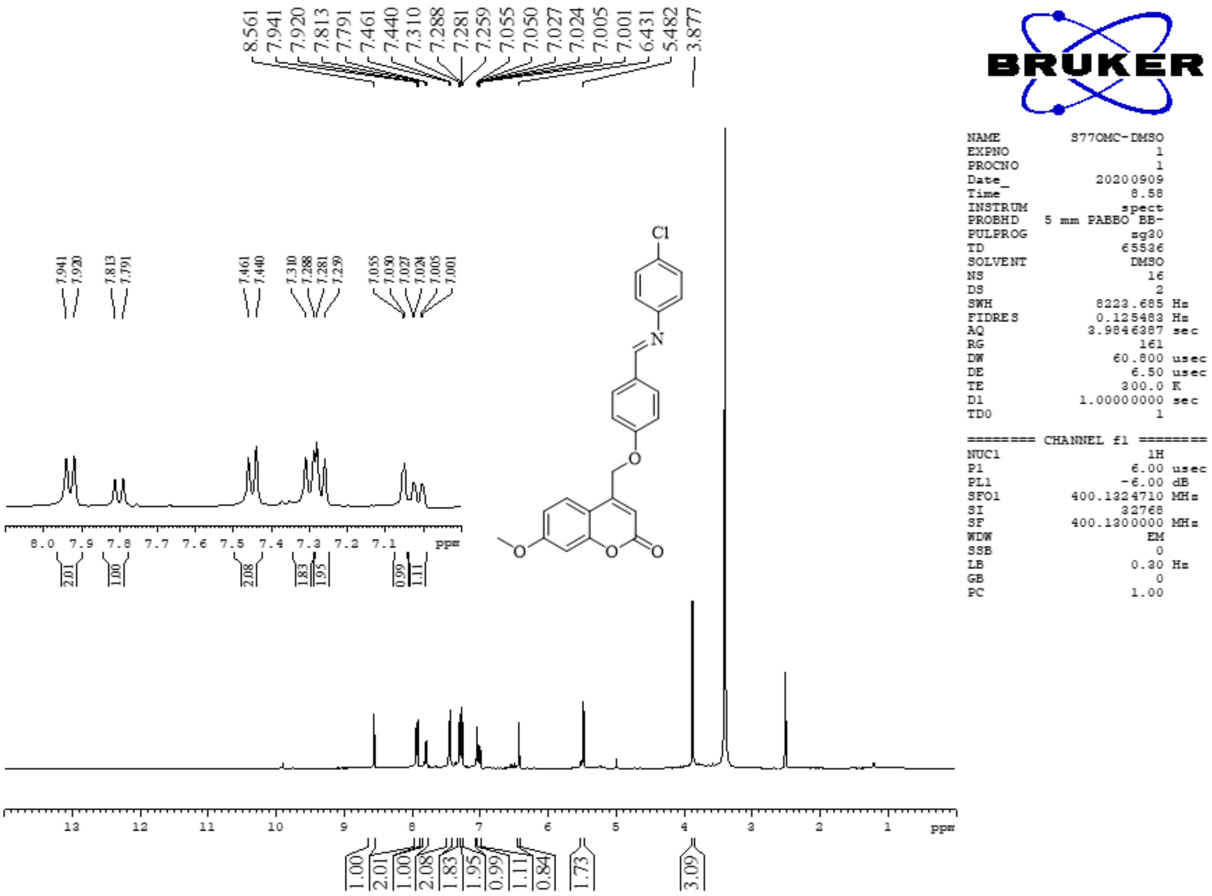

^{1}H NMR spectrum of (*E*)-4-((4-(((4-chlorophenyl)imino)methyl)phenoxy)methyl)-7-methoxy-2*H*-chromen-2-one
(13c)

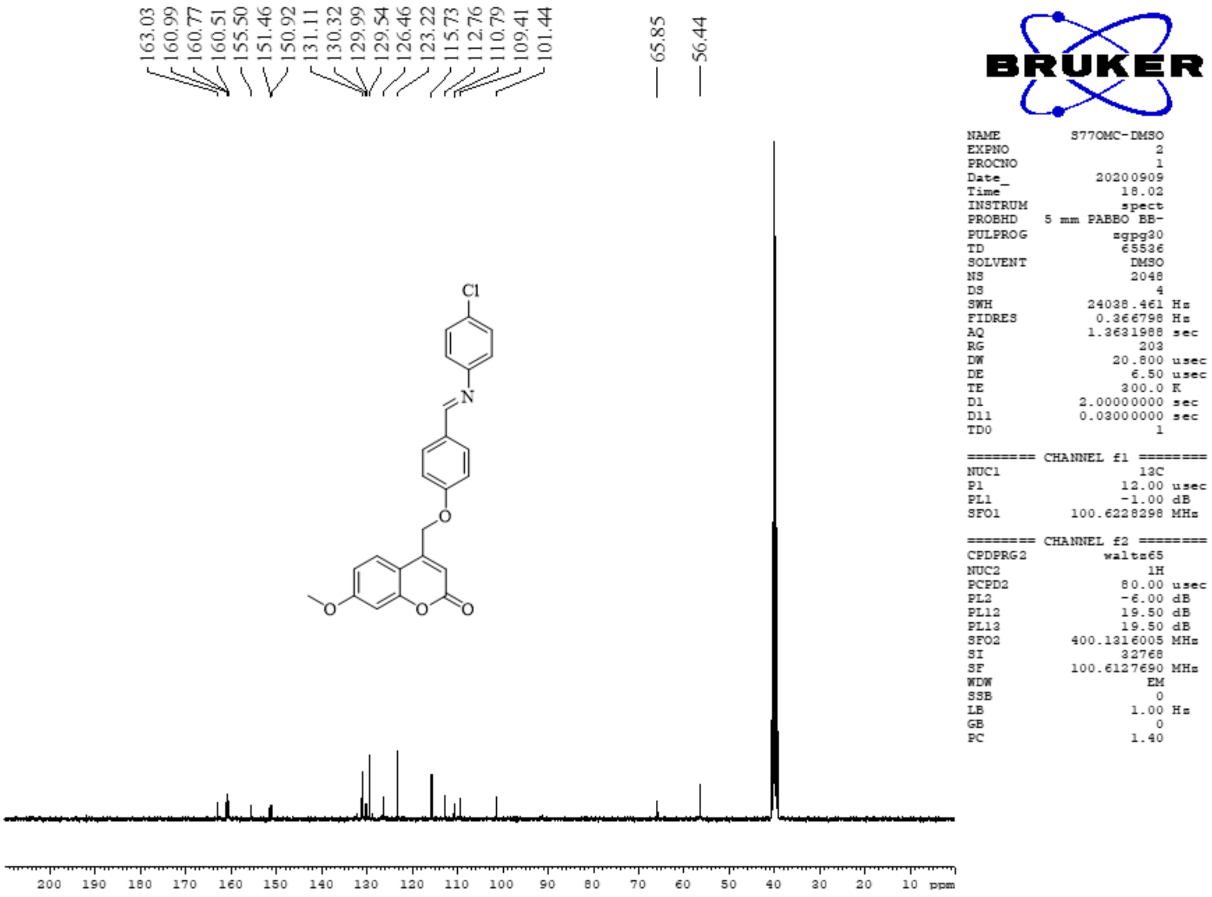

^{13}C NMR spectrum of (*E*)-4-((4-(((4-chlorophenyl)imino)methyl)phenoxy)methyl)-7-methoxy-2*H*-chromen-2-one
(13c)

Compound Table

Compound Label	RT	Mass	Abund	Formula	Tgt Mass	Diff (ppm)
Cpd 1: C24 H18 Cl N O4	0.254	419.092	49039	C24 H18 Cl N O4	419.0924	-1.06

Compound Label	*m/z*	RT	Algorithm	Mass
Cpd 1: C24 H18 Cl N O4	420.0995	0.254	Find By Formula	419.092

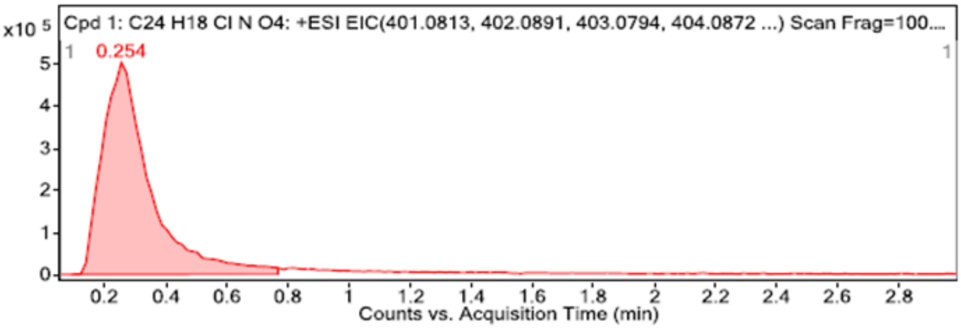

MS Zoomed Spectrum

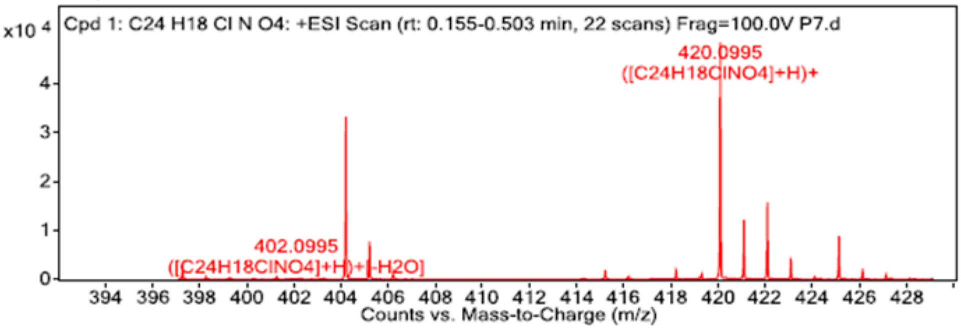

HRMS of (*E*)-4-((4-(((4-chlorophenyl)imino)methyl)phenoxy)methyl)-7-methoxy-2*H*-chromen-2-one **(13c)**

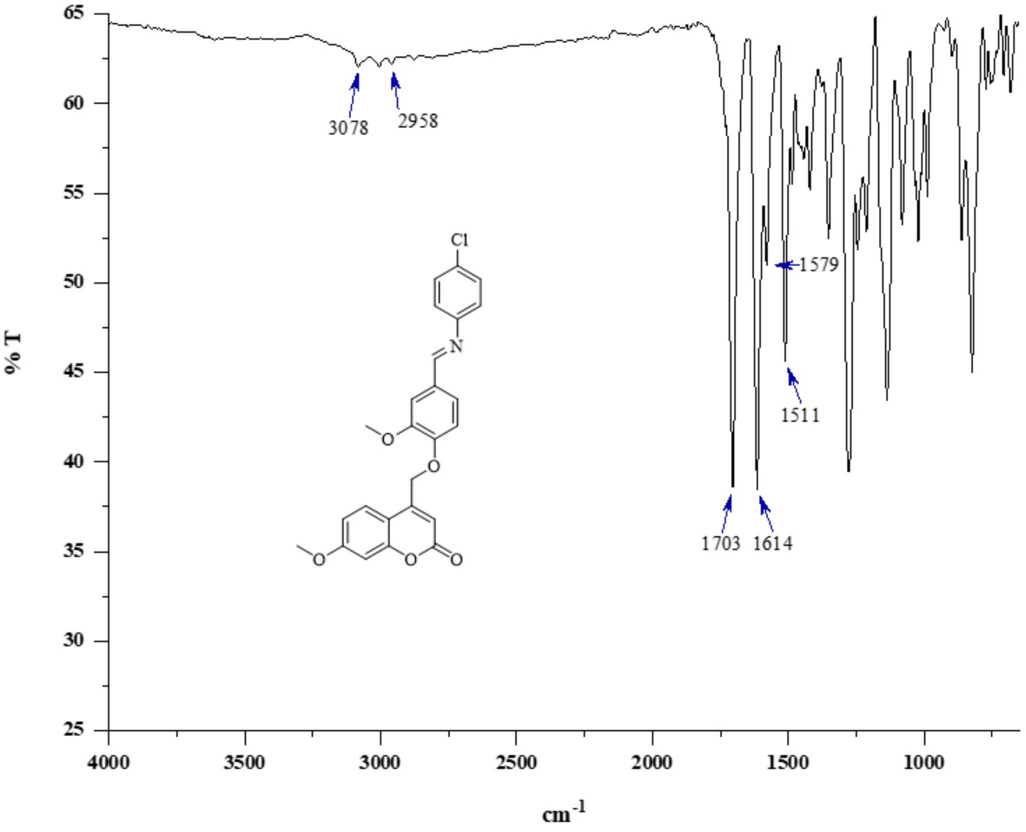

IR spectrum of (*E*)-4-((4-(((4-chlorophenyl)imino)methyl)-2-methoxy phenoxy)methyl)-7-methoxy-2*H*-chromen-2-one **(13d)**

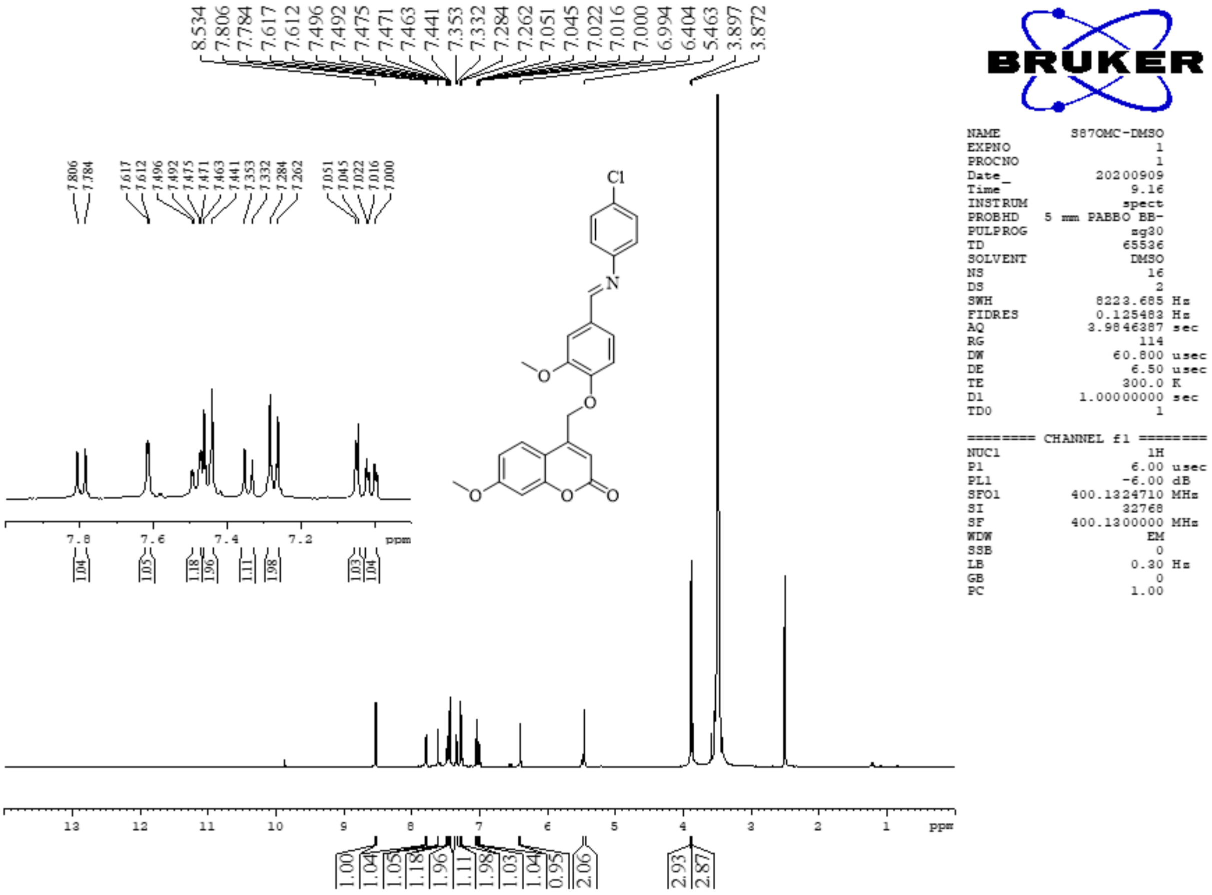

^{1}H NMR spectrum of (*E*)-4-((4-(((4-chlorophenyl)imino)methyl)-2-methoxy phenoxy)methyl)-7-methoxy-2*H*-chromen-2-one **(13d)**

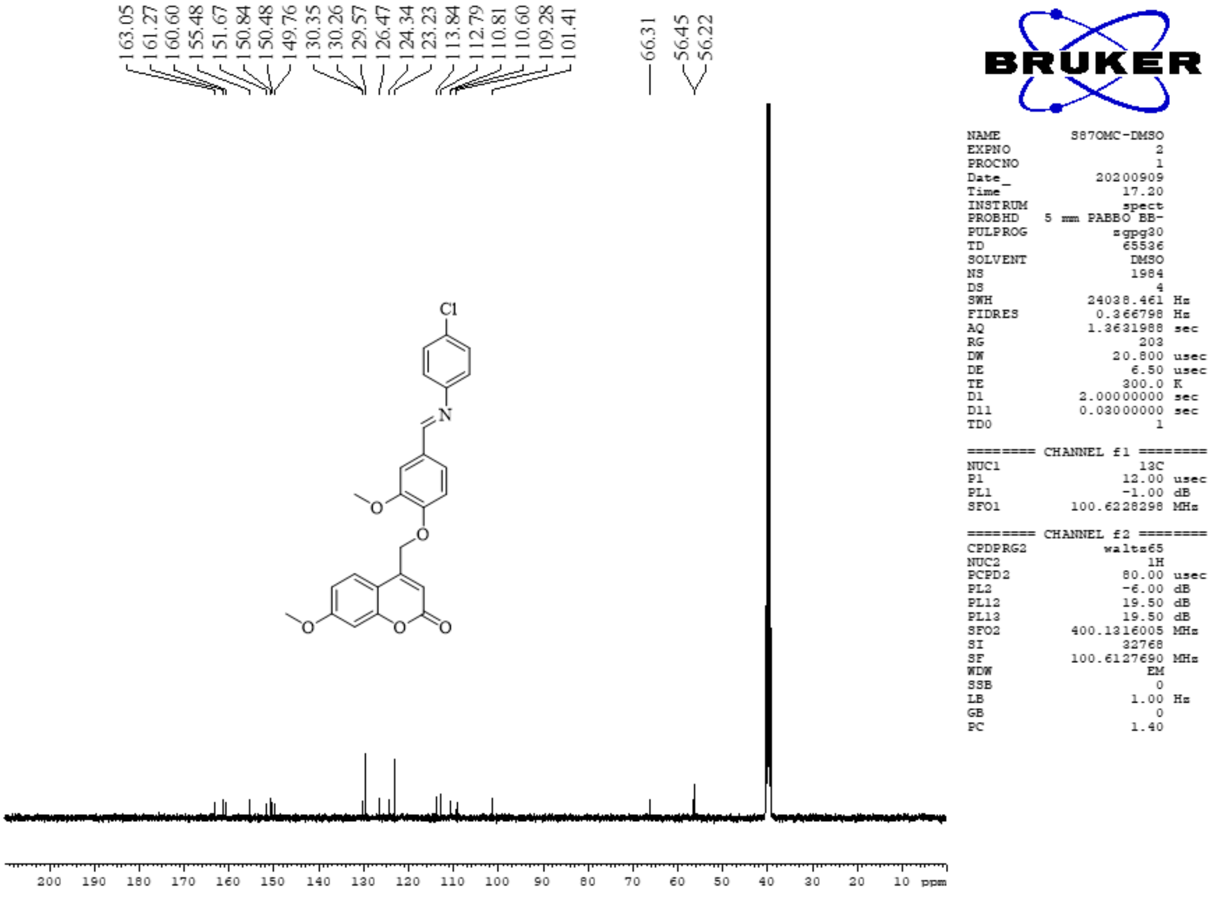

^{13}C NMR spectrum of (*E*)-4-((4-(((4-chlorophenyl)imino)methyl)-2-methoxy phenoxy)methyl)-7-methoxy-2*H*-chromen-2-one **(13d)**

Compound Table

Compound Label	RT	Mass	Abund	Formula	Tgt Mass	Diff (ppm)
Cpd 1: C25 H20 Cl N O5	0.243	449.1028	87486	C25 H20 Cl N O5	449.103	-0.47

Compound Label	*m/z*	RT	Algorithm	Mass
Cpd 1: C25 H20 Cl N O5	450.1102	0.243	Find By Formula	449.1028

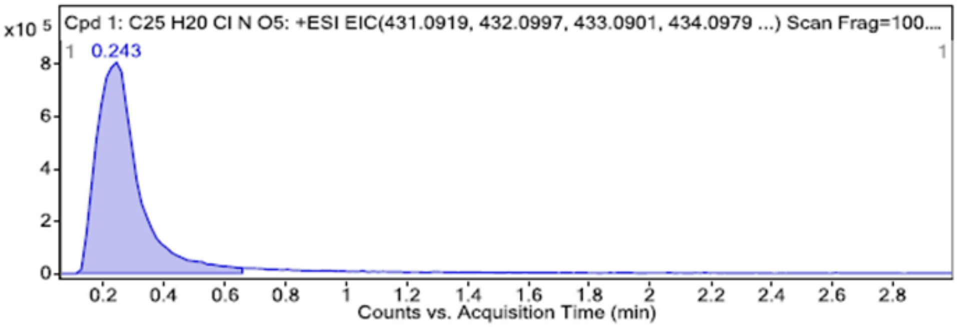

MS Zoomed Spectrum

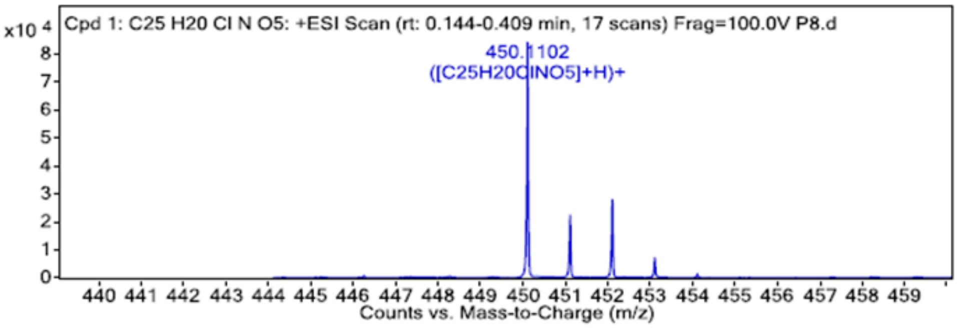

HRMS of (*E*)-4-((4-(((4-chlorophenyl)imino)methyl)-2-methoxy phenoxy)methyl)-7-methoxy-2*H*-chromen-2-one **(13d)**

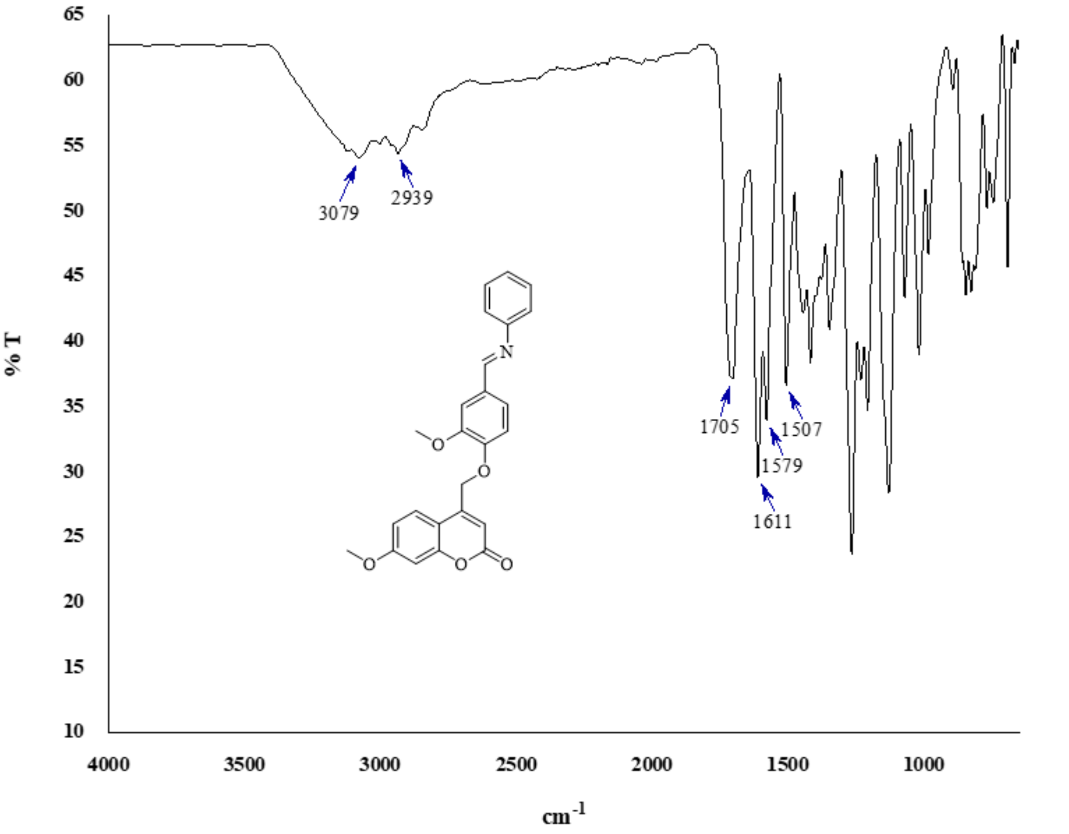

IR spectrum of (*E*)-7-methoxy-4-((2-methoxy-4-((phenylimino)methyl) phenoxy)methyl)-2*H*-chromen-2-one **(13e)**

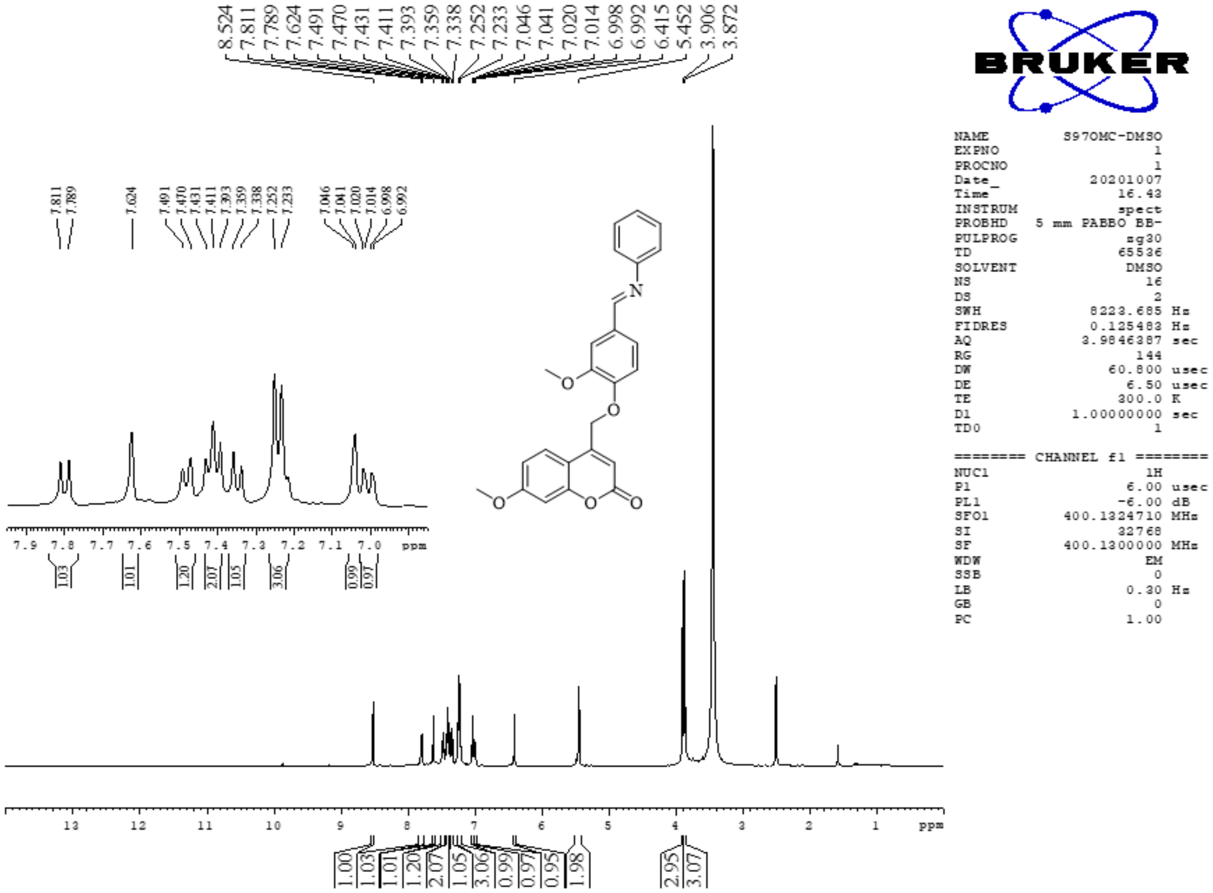

^{1}H NMR spectrum of (*E*)-7-methoxy-4-((2-methoxy-4-((phenylimino)methyl) phenoxy)methyl)-2*H*-chromen-2-one **(13e)**

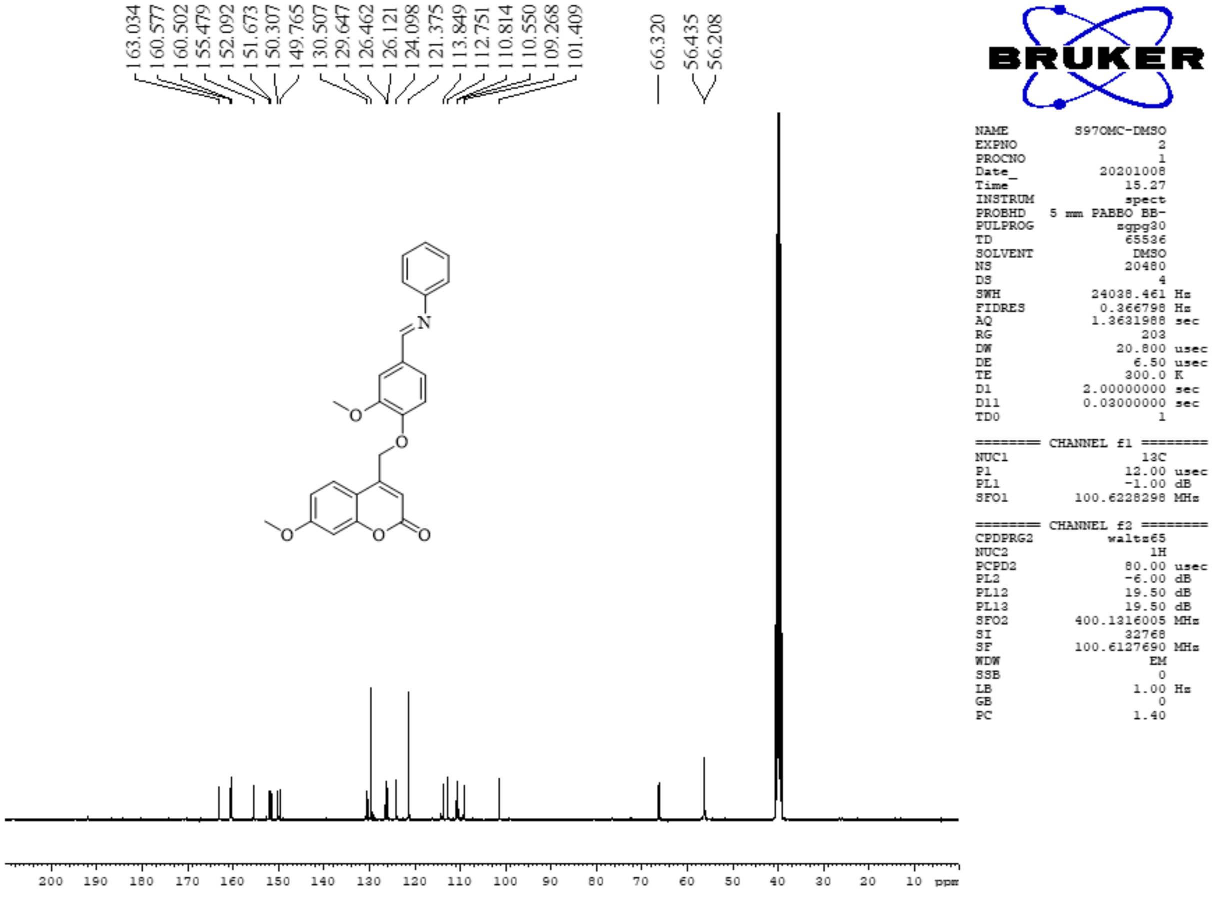

^{13}C NMR spectrum of (*E*)-7-methoxy-4-((2-methoxy-4-((phenylimino)methyl) phenoxy)methyl)-2*H*-chromen-2-one **(13e)**

Compound Table

Compound Label	RT	Mass	Abund	Formula	Tgt Mass	Diff (ppm)
Cpd 1: C25 H21 N O5	0.206	415.1421	287628	C25 H21 N O5	415.142	0.37

Compound Label	*m/z*	RT	Algorithm	Mass
Cpd 1: C25 H21 N O5	416.1495	0.206	Find By Formula	415.1421

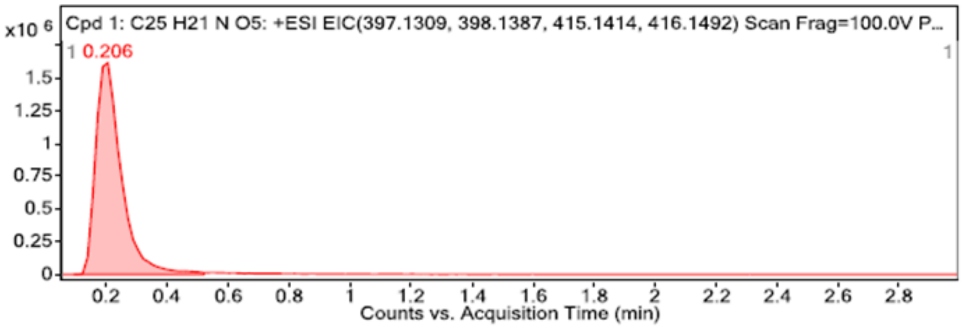

MS Zoomed Spectrum

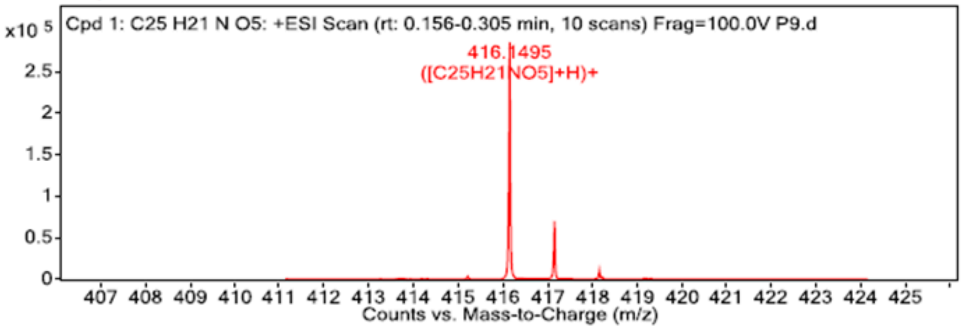

HRMS of (*E*)-7-methoxy-4-((2-methoxy-4-((phenylimino)methyl) phenoxy)methyl)-2*H*-chromen-2-one **(13e)**

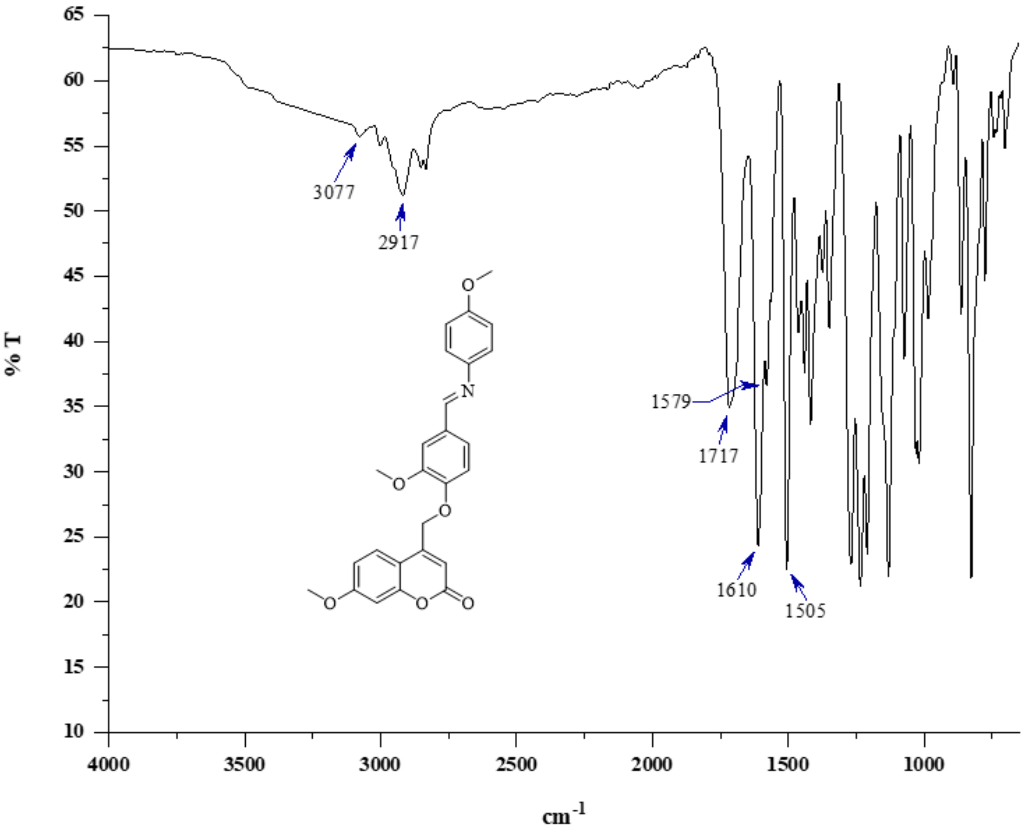

IR spectrum of (*E*)-7-methoxy-4-((2-methoxy-4-(((4-methoxyphenyl)imino) methyl)phenoxy)methyl)-2*H*-chromen-2-one **(13f)**

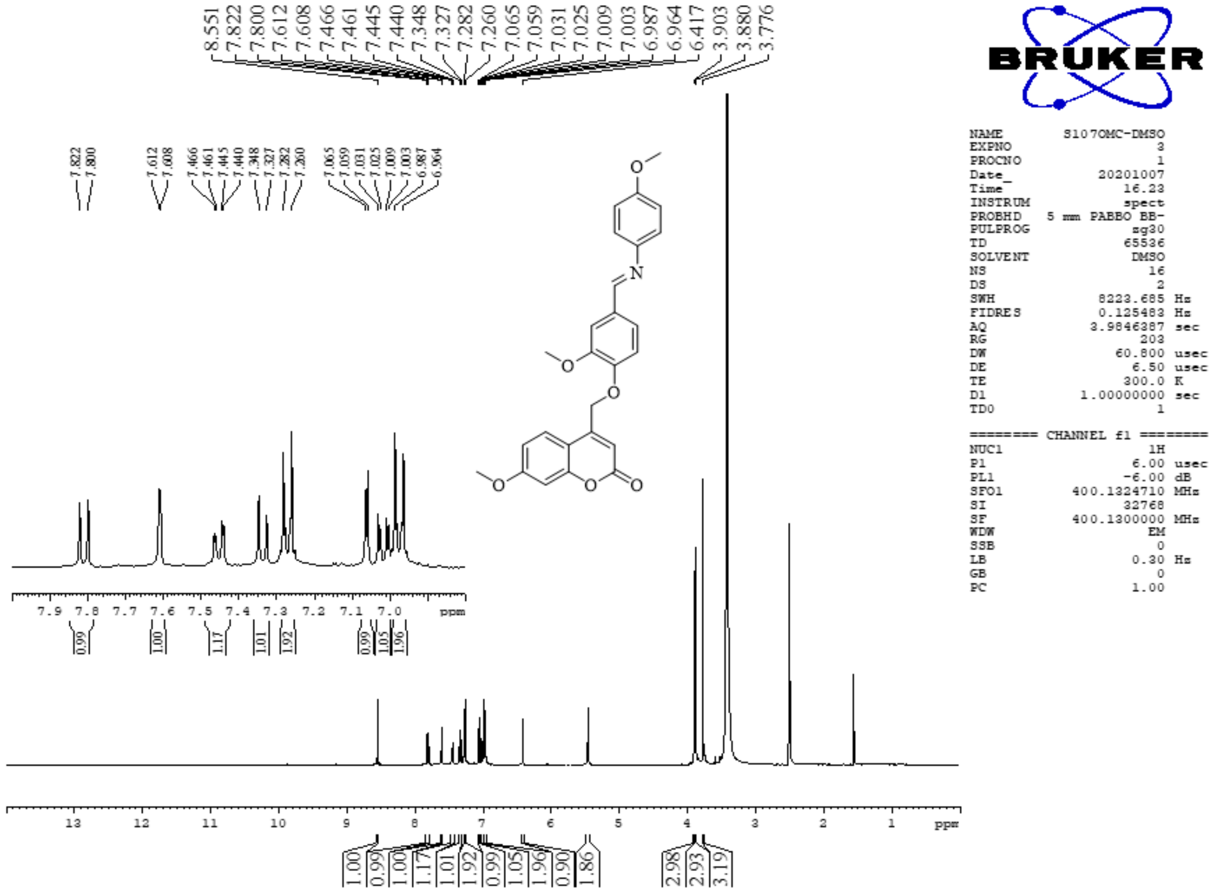

^{1}H NMR spectrum of (*E*)-7-methoxy-4-((2-methoxy-4-(((4-methoxyphenyl)imino) methyl)phenoxy)methyl)-2*H*-chromen-2-one **(13f)**

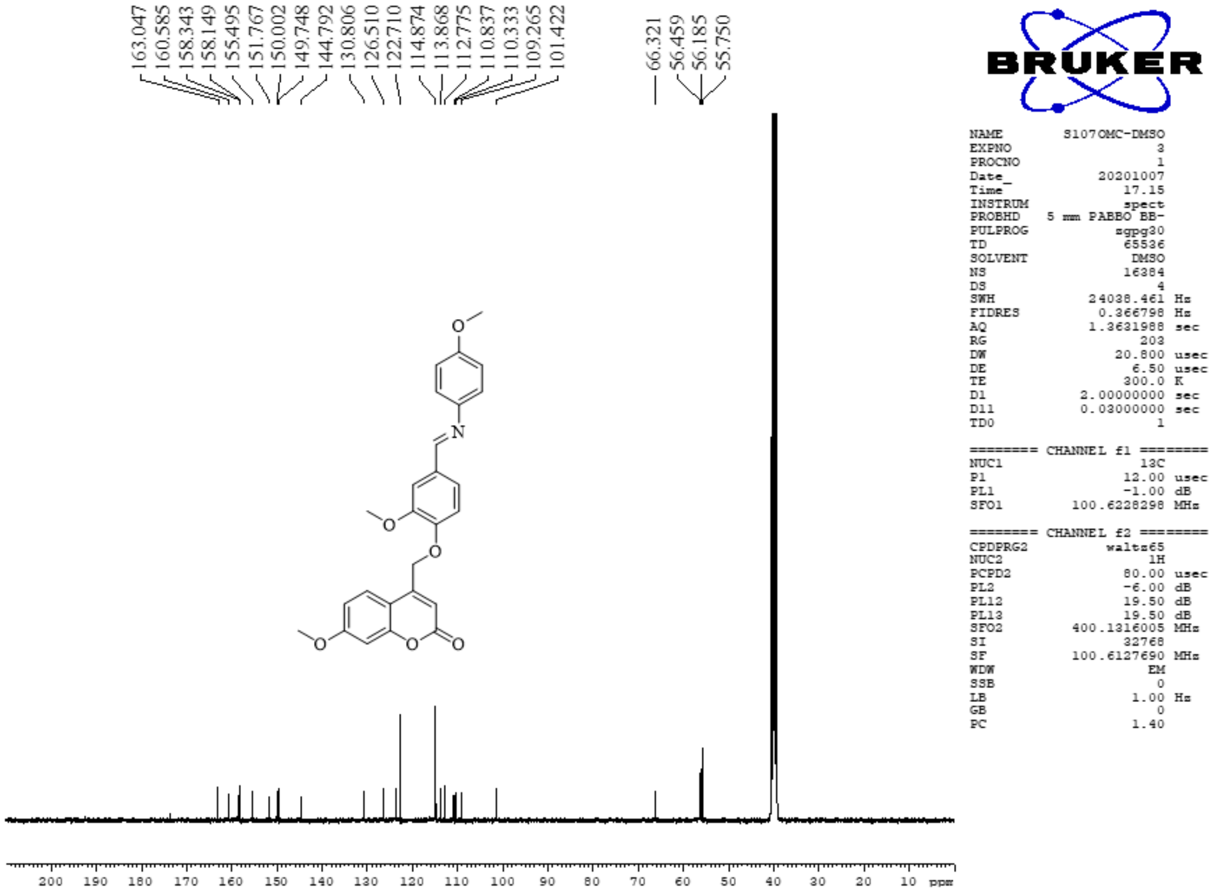

^{13}C NMR spectrum of (*E*)-7-methoxy-4-((2-methoxy-4-(((4-methoxyphenyl)imino) methyl)phenoxy)methyl)-2*H*-chromen-2-one **(13f)**

Compound Table

Compound Label	RT	Mass	Abund	Formula	Tgt Mass	Diff (ppm)
Cpd 1: C26 H23 N O6	0.193	445.1519	14309	C26 H23 N O6	445.1525	-1.53

Compound Label	*m/z*	RT	Algorithm	Mass
Cpd 1: C26 H23 N O6	446.1594	0.193	Find By Formula	445.1519

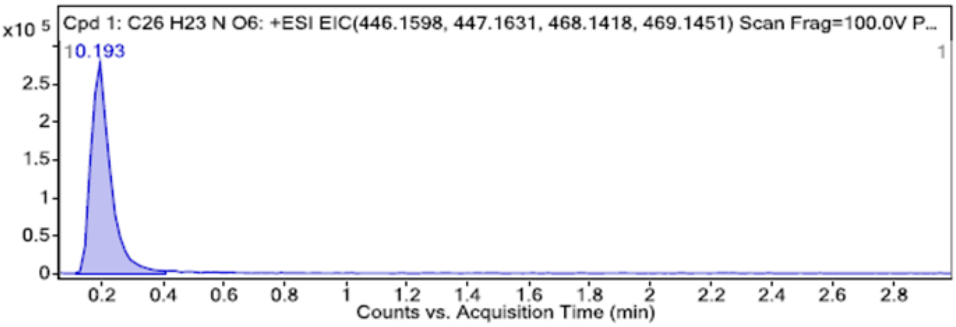

MS Zoomed Spectrum

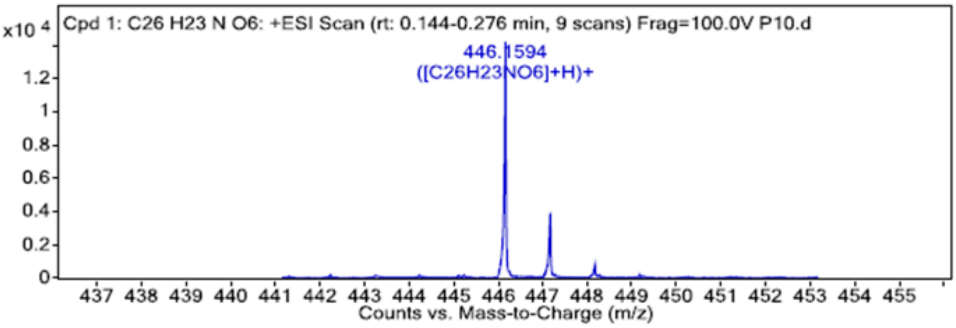

HRMS of (*E*)-7-methoxy-4-((2-methoxy-4-(((4-methoxyphenyl)imino) methyl)phenoxy)methyl)-2*H*-chromen-2-one **(13f)**

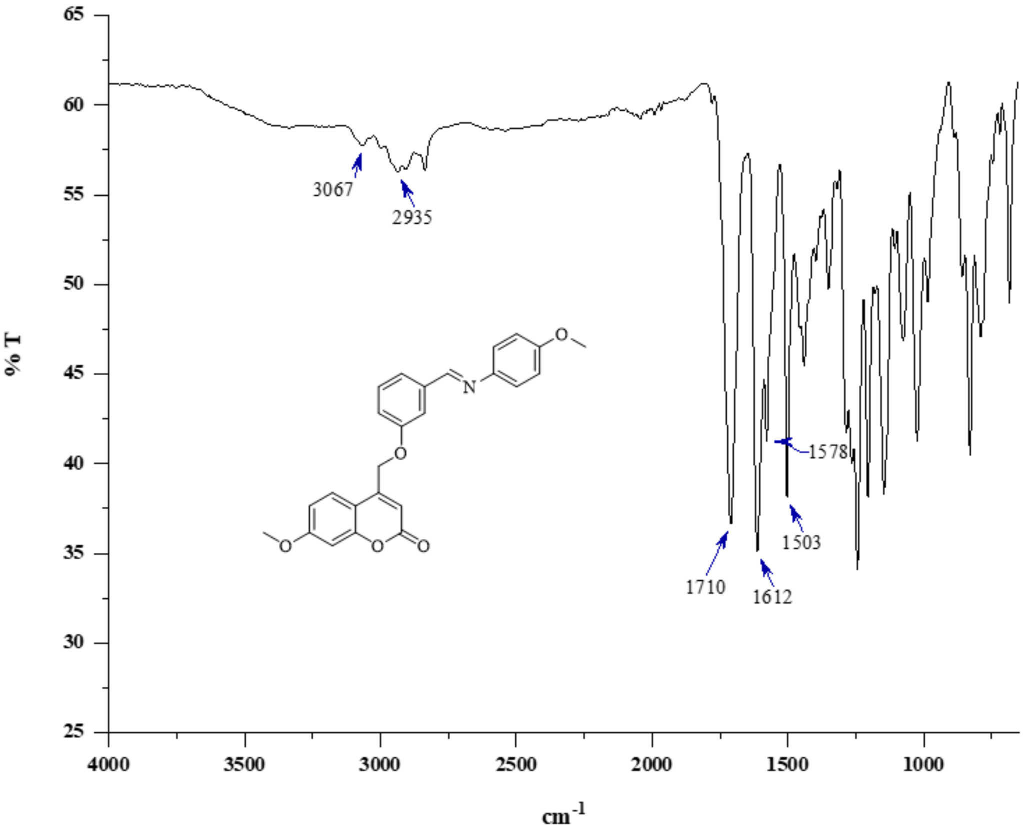

IR spectrum of (*E*)-7-methoxy-4-((3-(((4-methoxyphenyl)imino)methyl) phenoxy)methyl)-2*H*-chromen-2-one **(13g)**

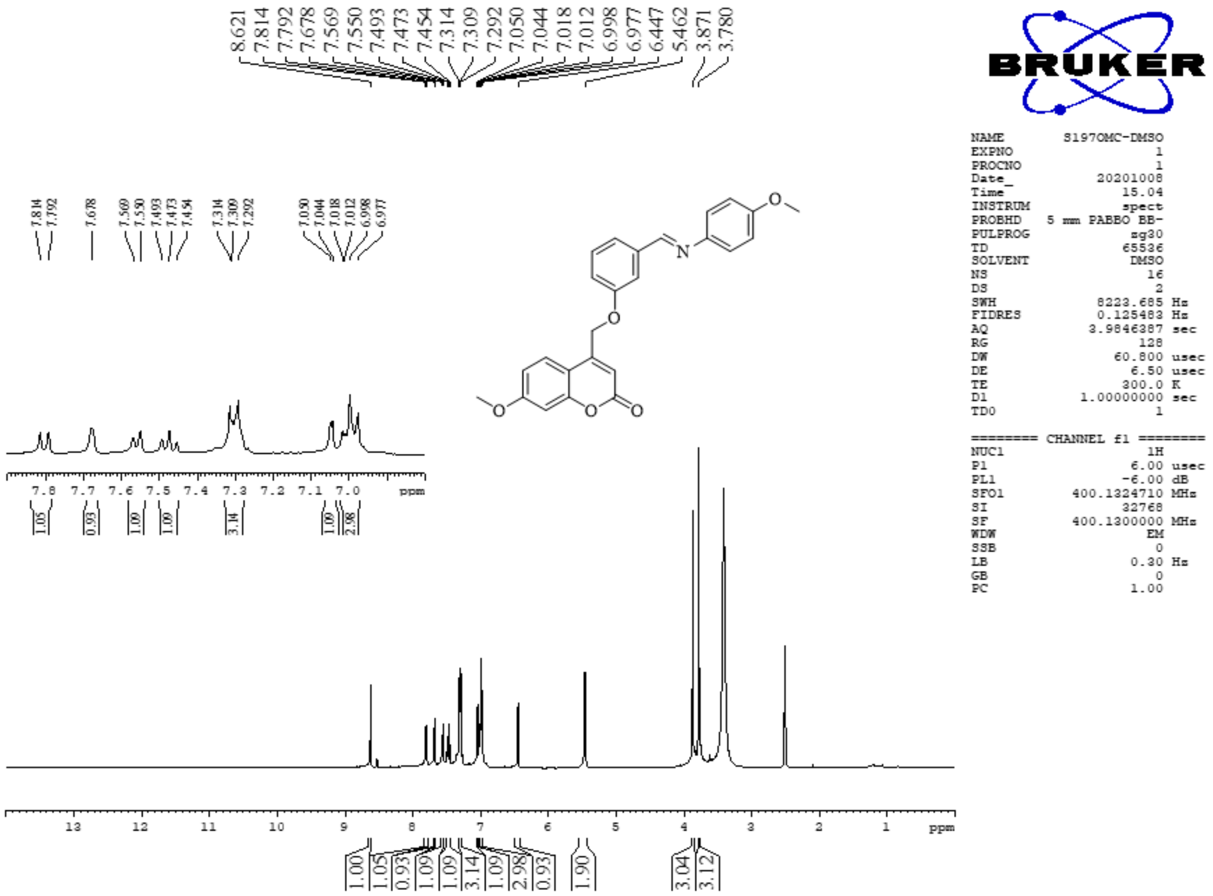

^{1}H NMR spectrum of (*E*)-7-methoxy-4-((3-(((4-methoxyphenyl)imino)methyl) phenoxy)methyl)-2*H*-chromen-2-one **(13g)**

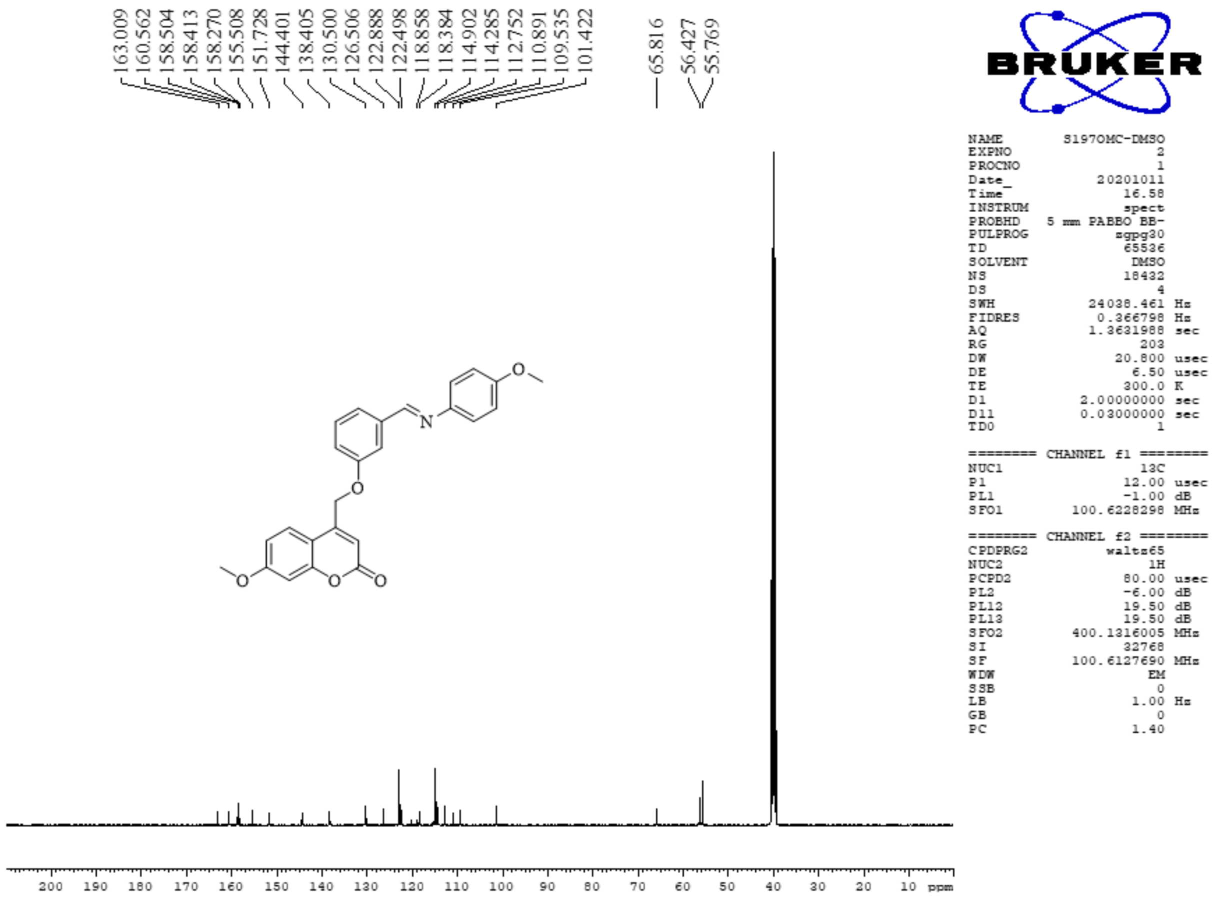

^{13}C NMR spectrum of (*E*)-7-methoxy-4-((3-(((4-methoxyphenyl)imino)methyl) phenoxy)methyl)-2*H*-chromen-2-one **(13g)**

Compound Table

Compound Label	RT	Mass	Abund	Formula	Tgt Mass	Diff (ppm)
Cpd 1: C25 H21 N O5	0.191	415.1417	22797	C25 H21 N O5	415.142	-0.69

Compound Label	*m/z*	RT	Algorithm	Mass
Cpd 1: C25 H21 N O5	416.1491	0.191	Find By Formula	415.1417

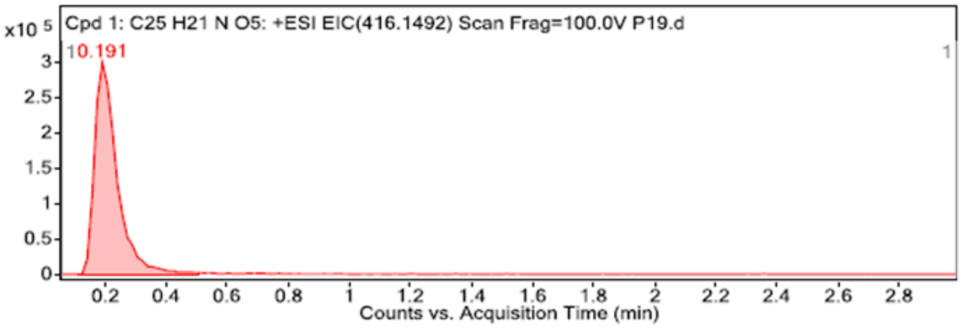

MS Zoomed Spectrum

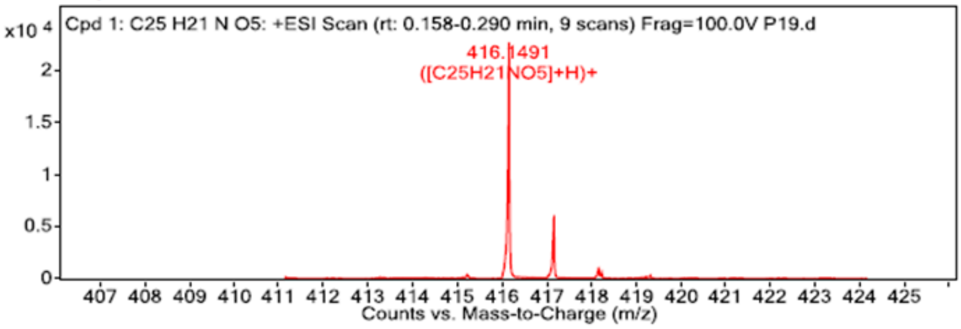

HRMS of (*E*)-7-methoxy-4-((3-(((4-methoxyphenyl)imino)methyl) phenoxy)methyl)-2*H*-chromen-2-one **(13g)**

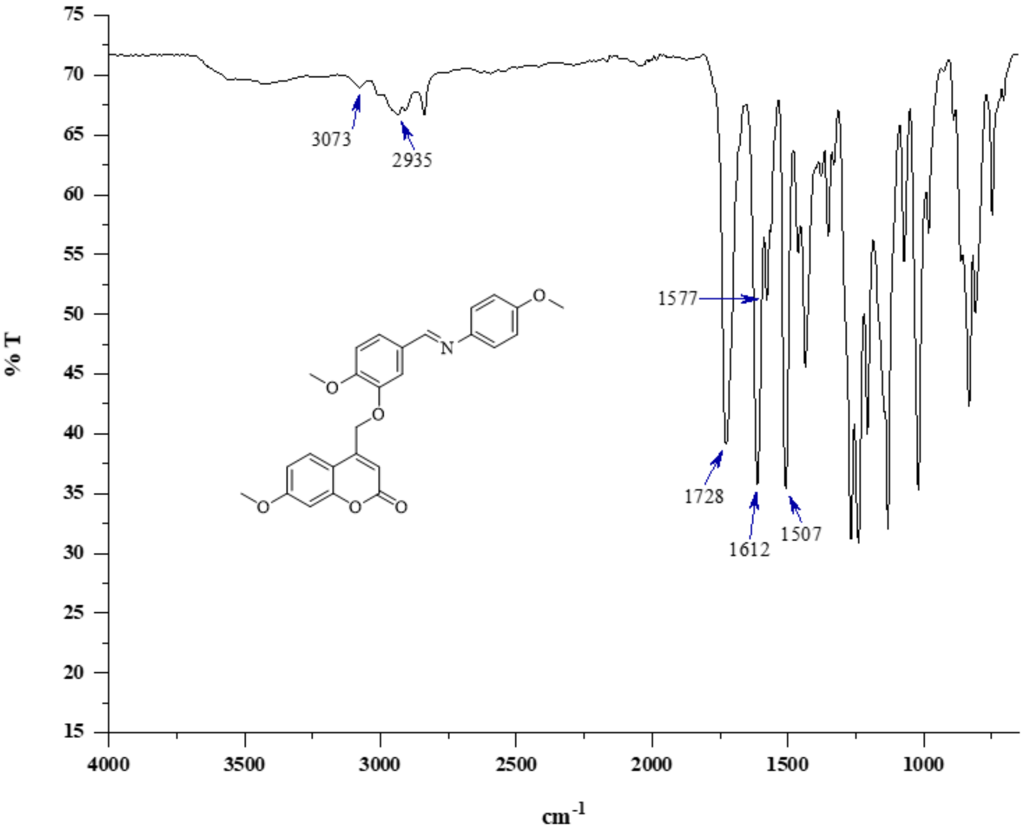

IR spectrum of (*E*)-7-methoxy-4-((2-methoxy-5-(((4-methoxyphenyl)imino) methyl)phenoxy)methyl)-2*H*-chromen-2-one **(13h)**

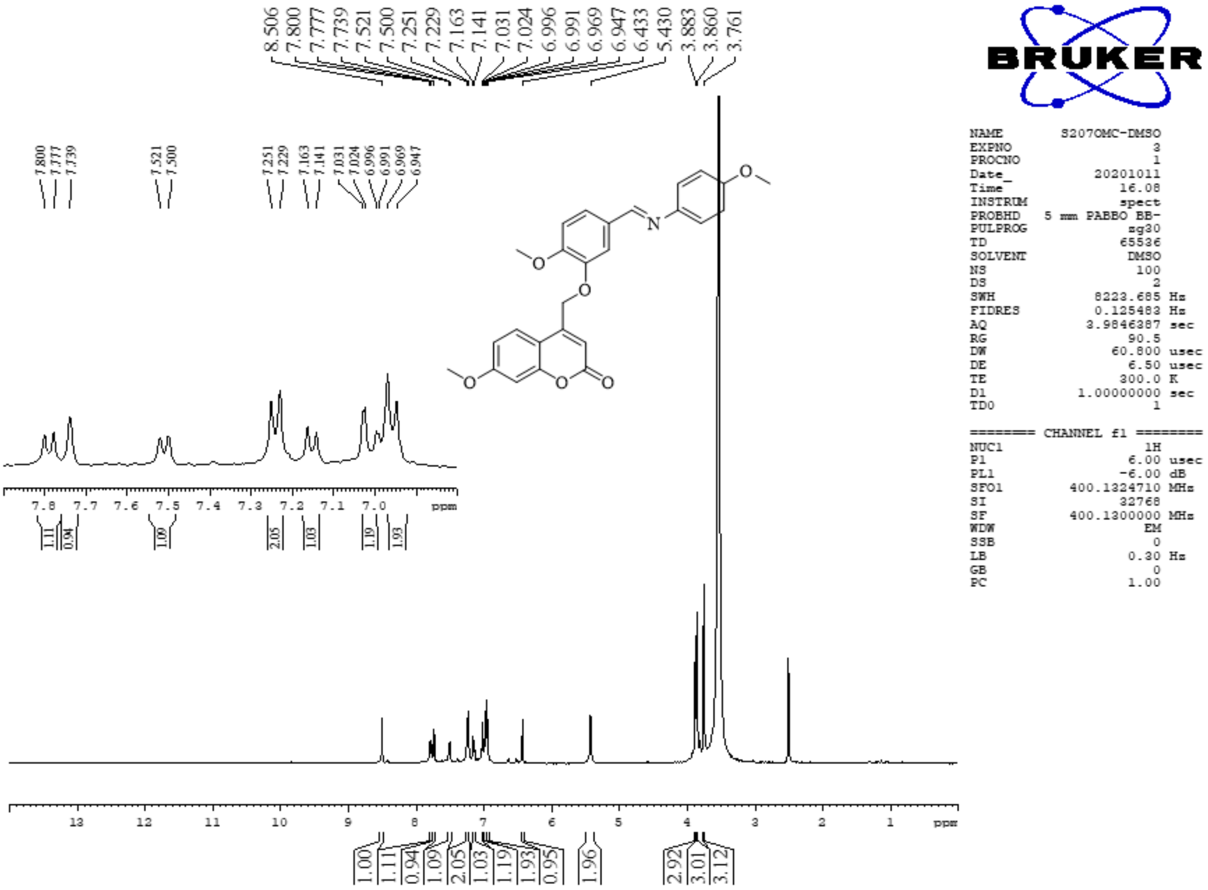

^{1}H NMR spectrum of (*E*)-7-methoxy-4-((2-methoxy-5-(((4-methoxyphenyl)imino) methyl)phenoxy)methyl)-2*H*-chromen-2-one **(13h)**

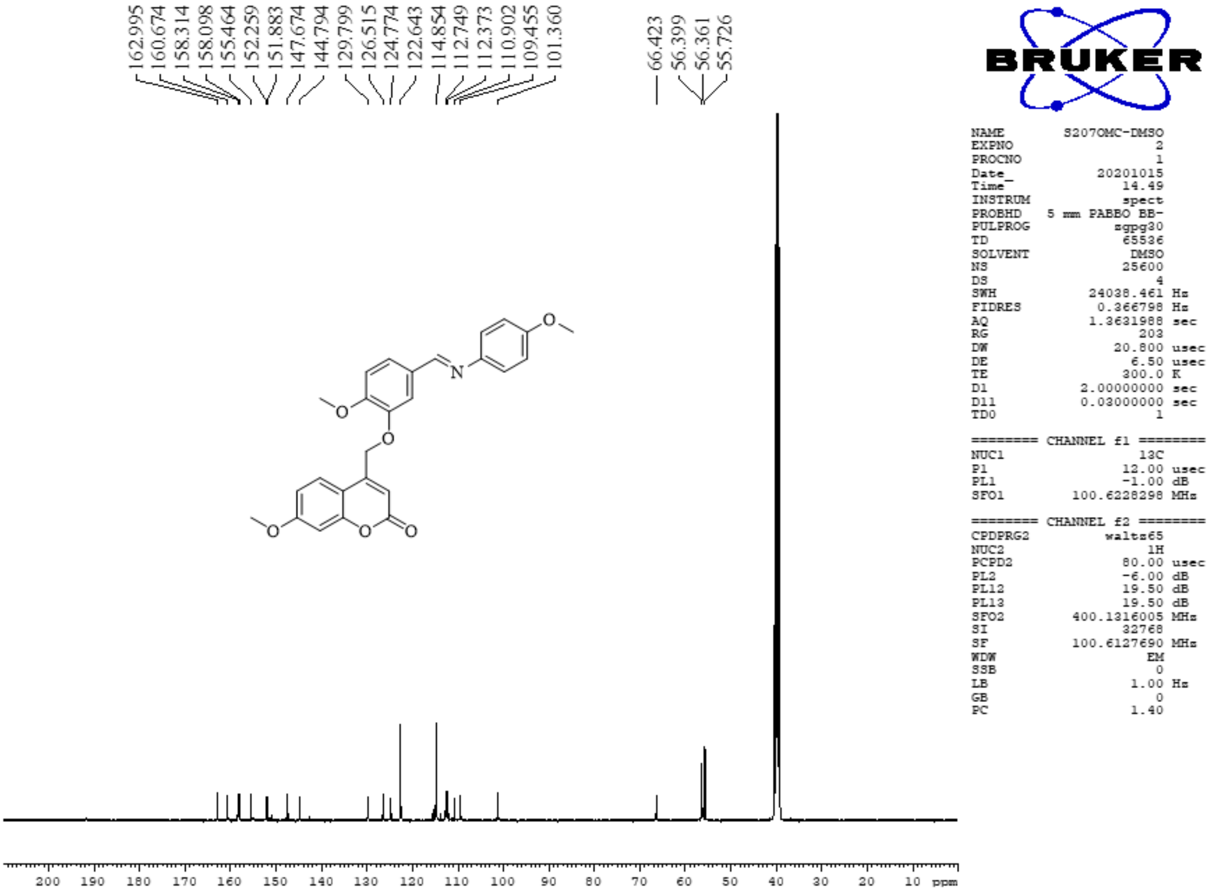

^{13}C NMR spectrum of (*E*)-7-methoxy-4-((2-methoxy-5-(((4-methoxyphenyl)imino) methyl)phenoxy)methyl)-2*H*-chromen-2-one **(13h)**

Compound Table

Compound Label	RT	Mass	Abund	Formula	Tgt Mass	Diff (ppm)
Cpd 1: C26 H23 N O6	0.191	445.1512	8662	C26 H23 N O6	445.1525	-3

Compound Label	*m/z*	RT	Algorithm	Mass
Cpd 1: C26 H23 N O6	446.1585	0.191	Find By Formula	445.1512

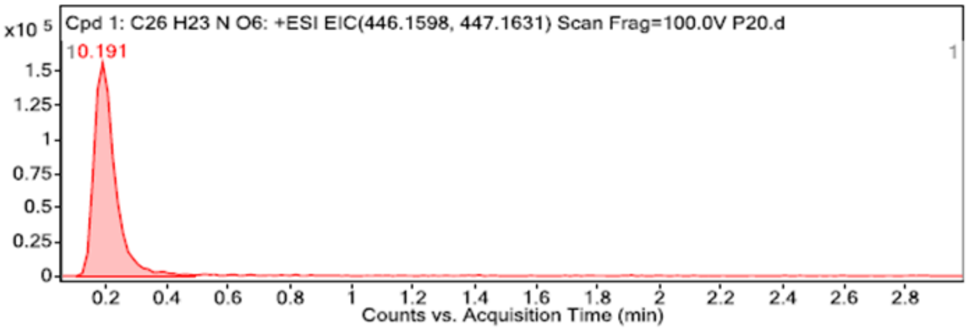

MS Zoomed Spectrum

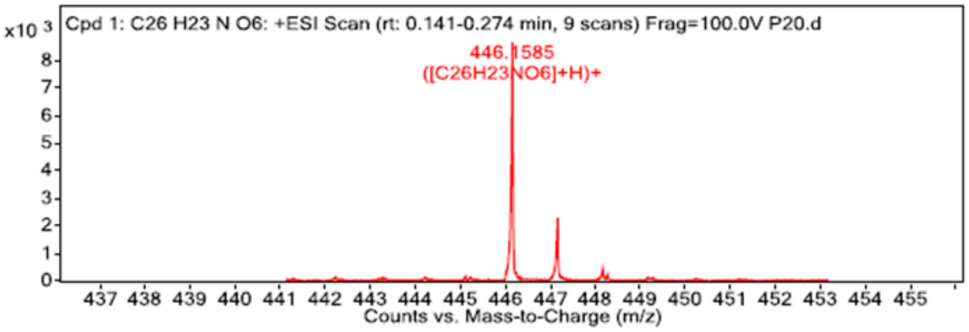

HRMS of (*E*)-7-methoxy-4-((2-methoxy-5-(((4-methoxyphenyl)imino) methyl)phenoxy)methyl)-2*H*-chromen-2-one **(13h)**

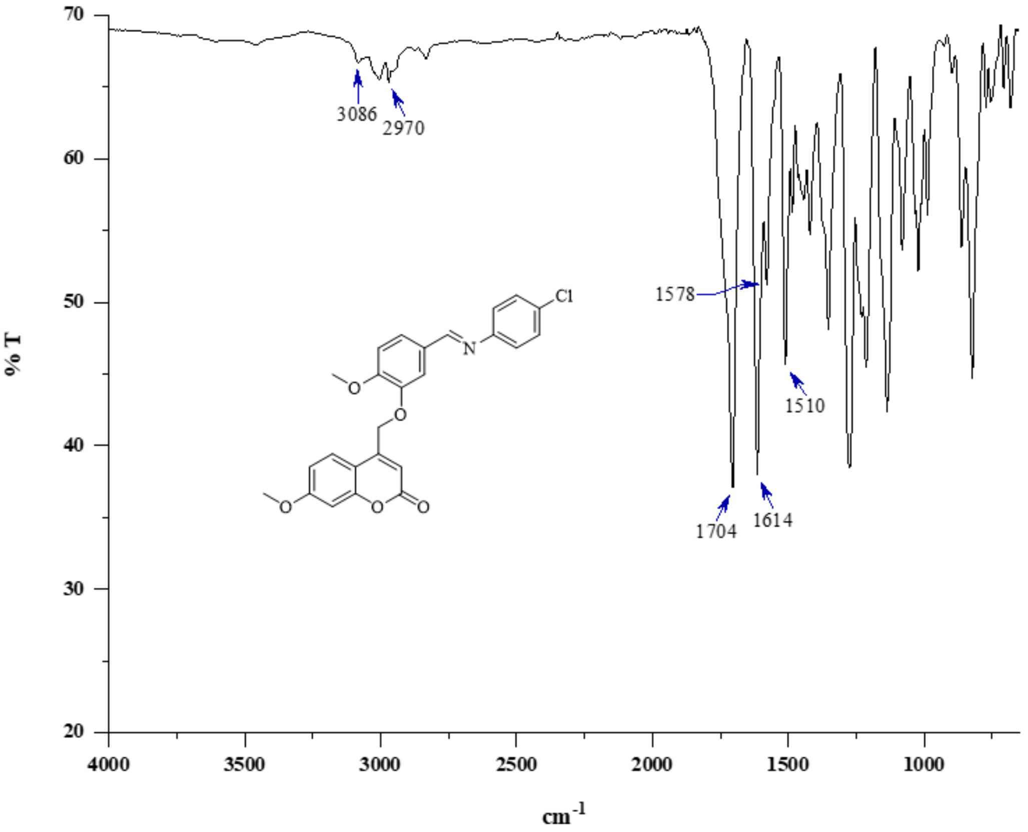

IR spectrum of (*E*)-4-((5-(((4-chlorophenyl)imino)methyl)-2-methoxy phenoxy)methyl)-7-methoxy-2*H*-chromen-2-one **(13i)**

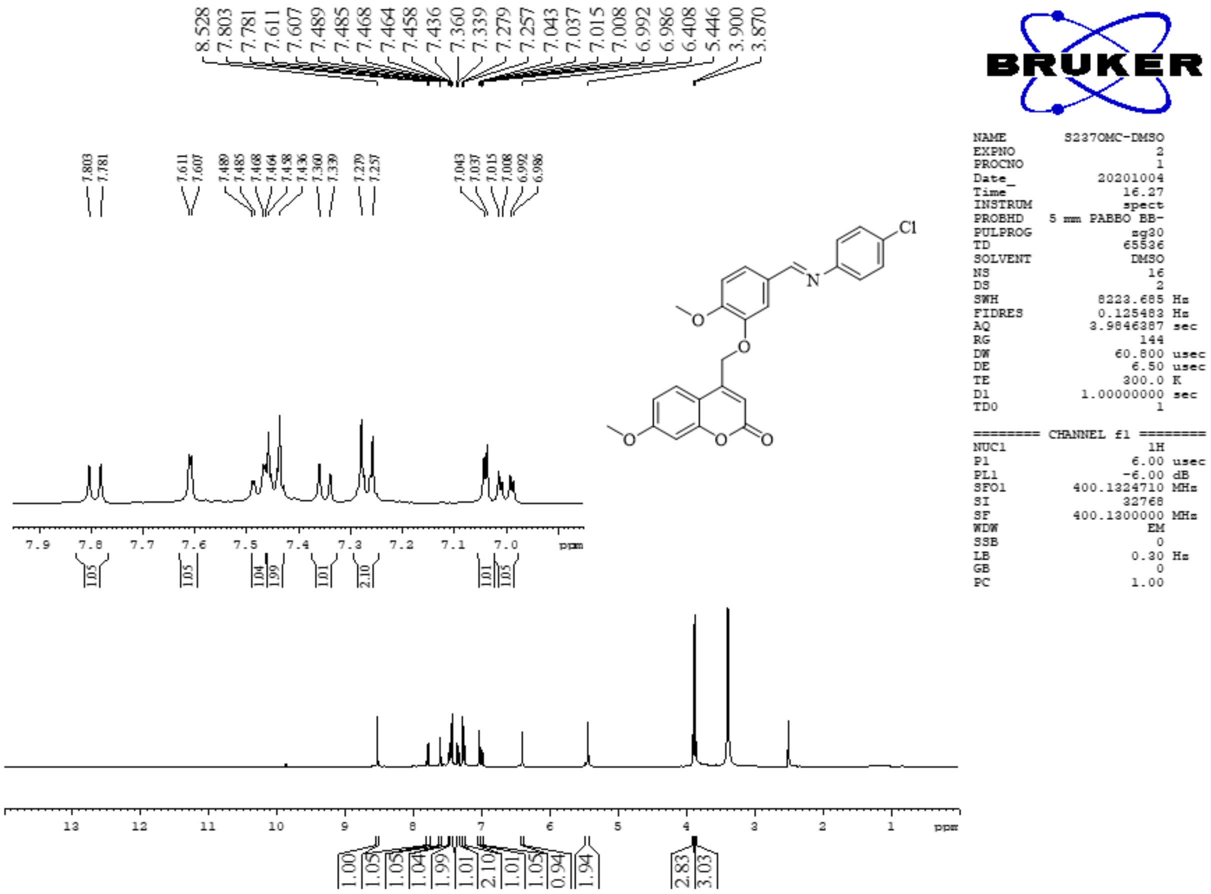

^{1}H NMR spectrum of (*E*)-4-((5-(((4-chlorophenyl)imino)methyl)-2-methoxy phenoxy)methyl)-7-methoxy-2*H*-chromen-2-one **(13i)**

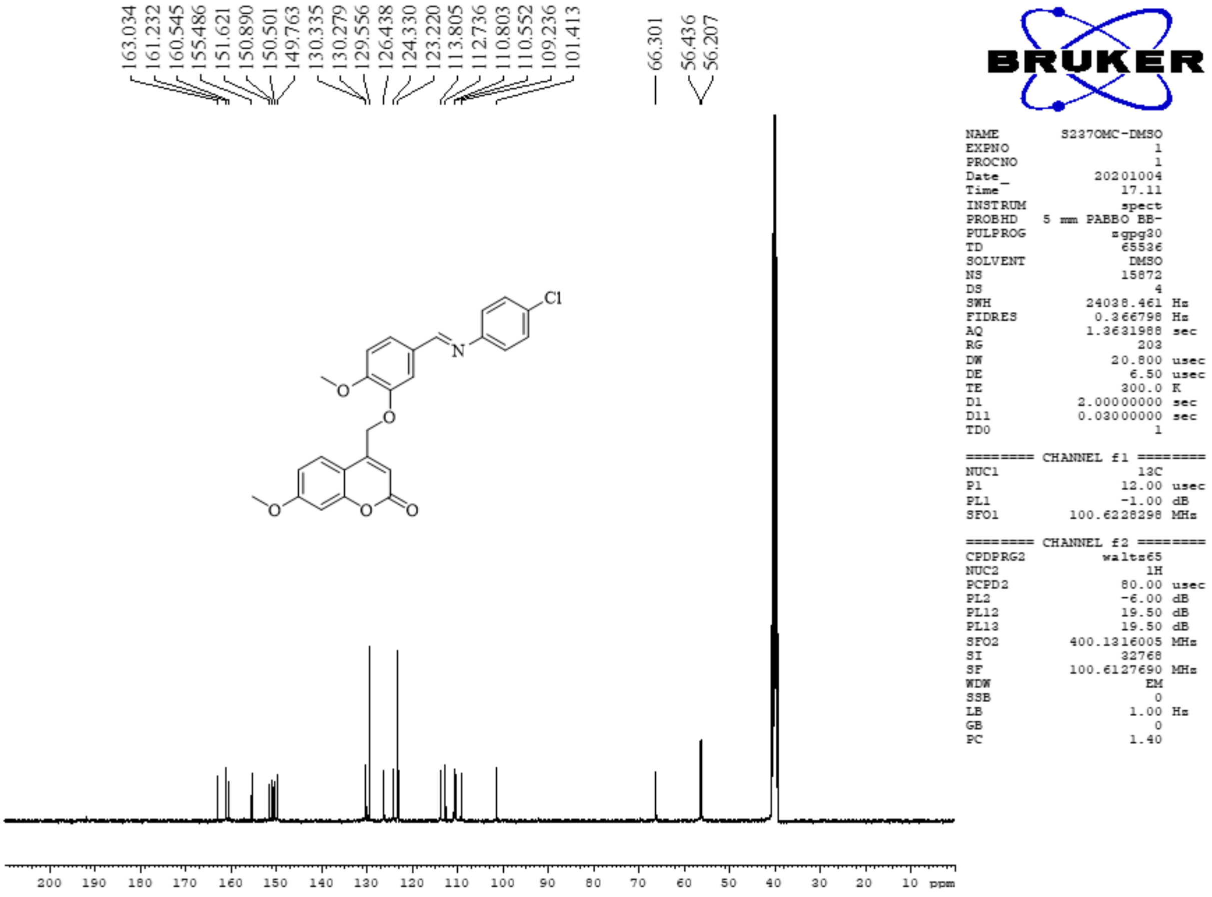

^{13}C NMR spectrum of (*E*)-4-((5-(((4-chlorophenyl)imino)methyl)-2-methoxy phenoxy)methyl)-7-methoxy-2*H*-chromen-2-one **(13i)**

Compound Table

Compound Label	RT	Mass	Abund	Formula	Tgt Mass	Diff (ppm)
Cpd 1: C25 H20 Cl N O5	0.201	449.1029	691	C25 H20 Cl N O5	449.103	-0.19

Compound Label	*m/z*	RT	Algorithm	Mass
Cpd 1: C25 H20 Cl N O5	450.1099	0.201	Find By Formula	449.1029

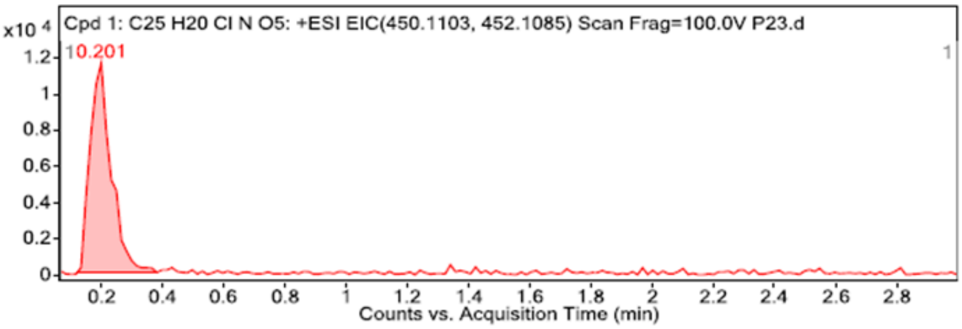

MS Zoomed Spectrum

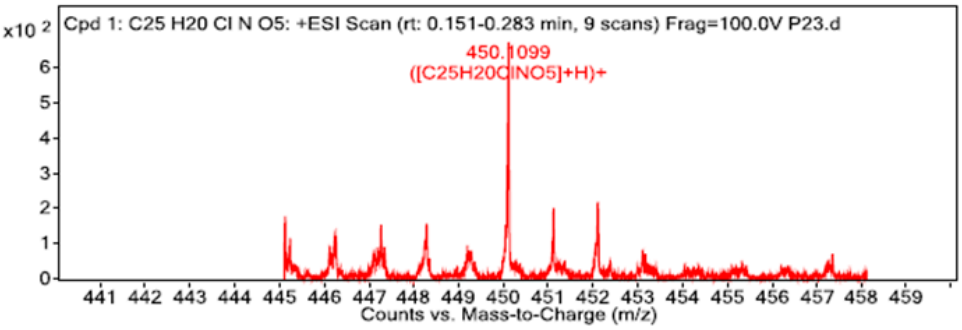

HRMS of (*E*)-4-((5-(((4-chlorophenyl)imino)methyl)-2-methoxy phenoxy)methyl)-7-methoxy-2*H*-chromen-2-one **(13i)**

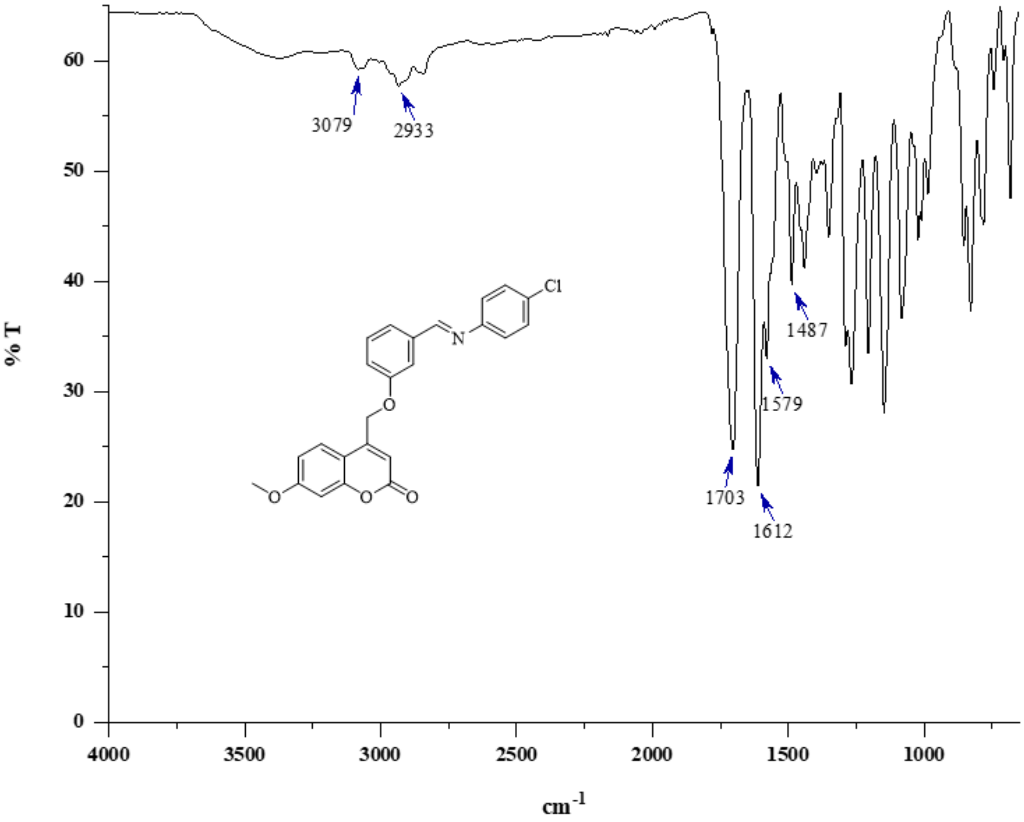

IR spectrum of (*E*)-4-((3-(((4-chlorophenyl)imino)methyl)phenoxy)methyl)-7-methoxy-2*H*-chromen-2-one **(13j)**

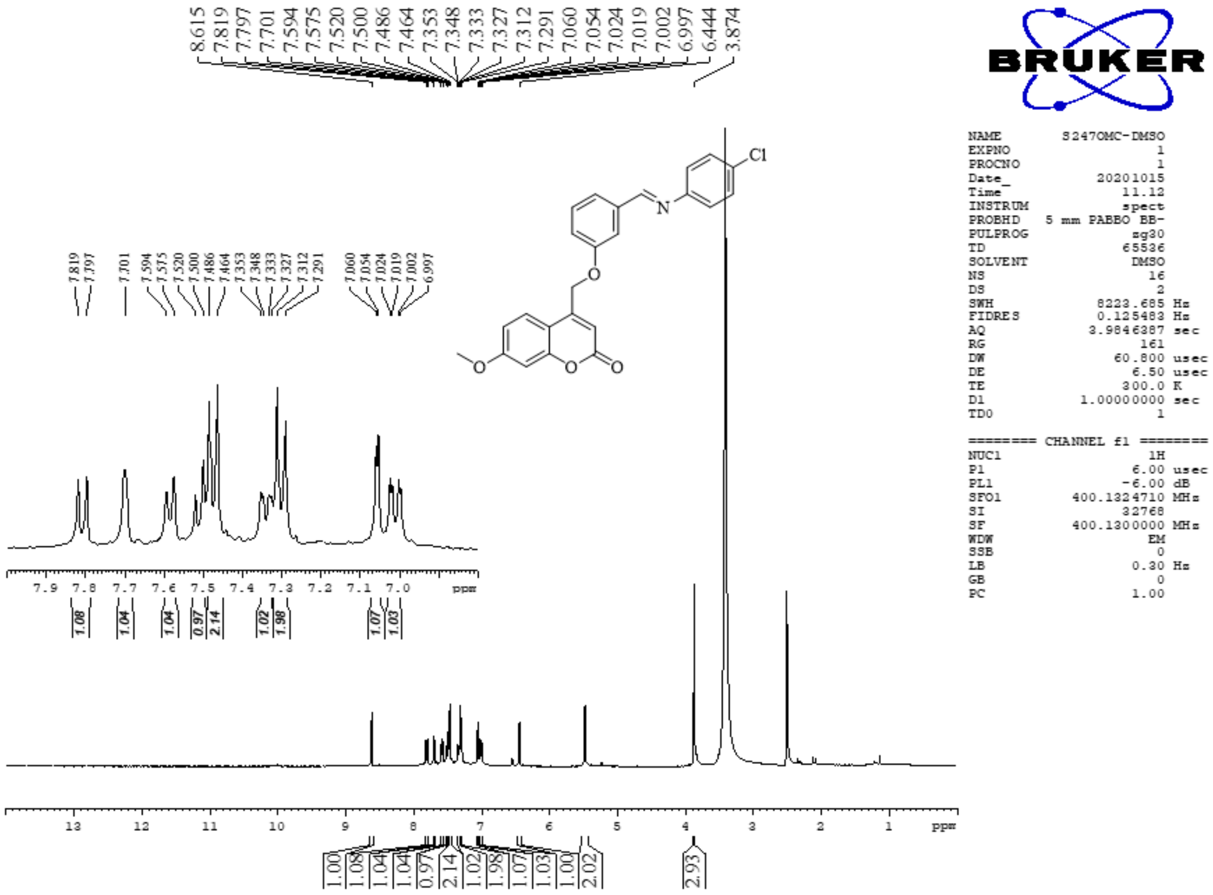

^{1}H NMR spectrum of (*E*)-4-((3-(((4-chlorophenyl)imino)methyl)phenoxy)methyl)-7-methoxy-2*H*-chromen-2-one **(13j)**

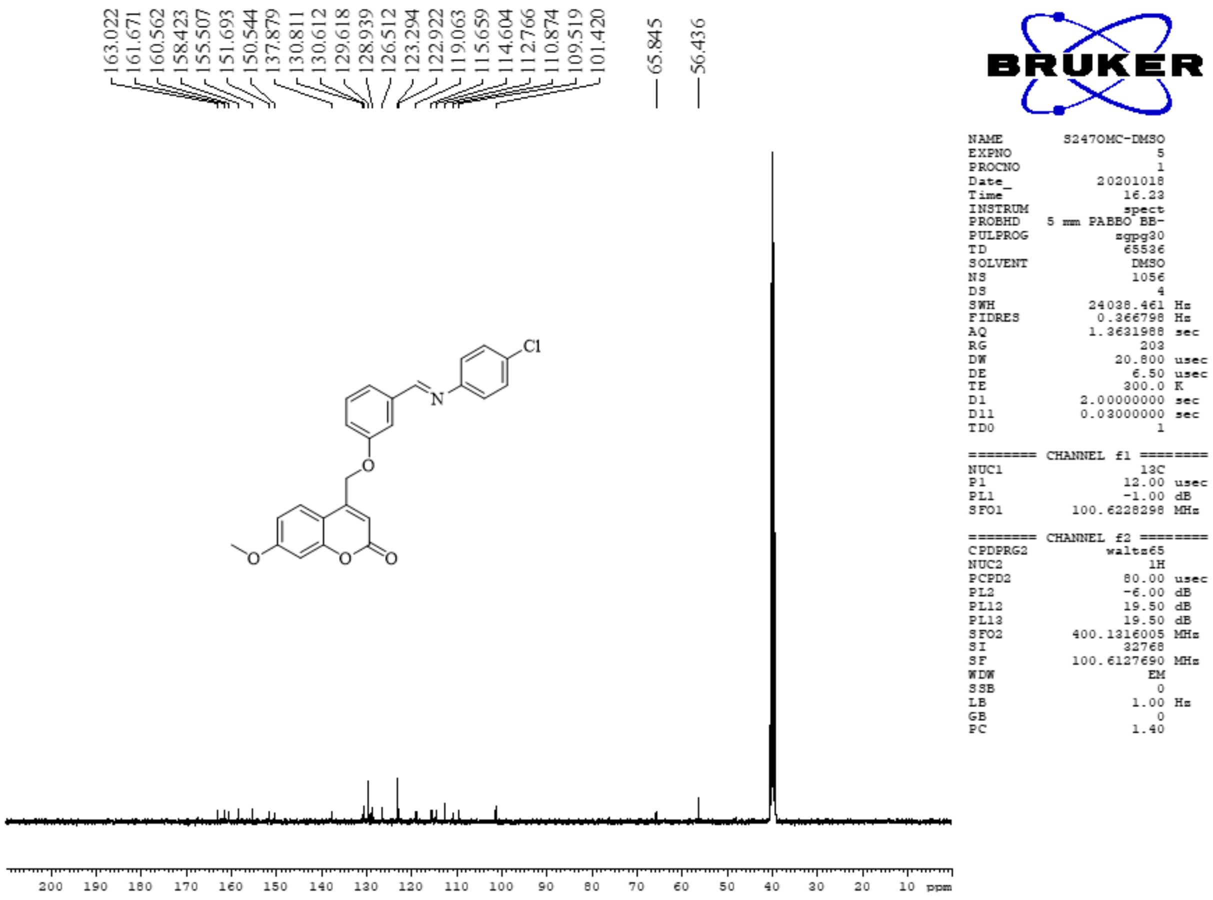

^{13}C NMR spectrum of (*E*)-4-((3-(((4-chlorophenyl)imino)methyl)phenoxy)methyl)-7-methoxy-2*H*-chromen-2-one **(13j)**

Compound Table

Compound Label	RT	Mass	Abund	Formula	Tgt Mass	Diff (ppm)
Cpd 1: C24 H18 Cl N O4	0.198	419.0908	429	C24 H18 Cl N O4	419.0924	-3.79

Compound Label	*m/z*	RT	Algorithm	Mass
Cpd 1: C24 H18 Cl N O4	420.0972	0.198	Find By Formula	419.0908

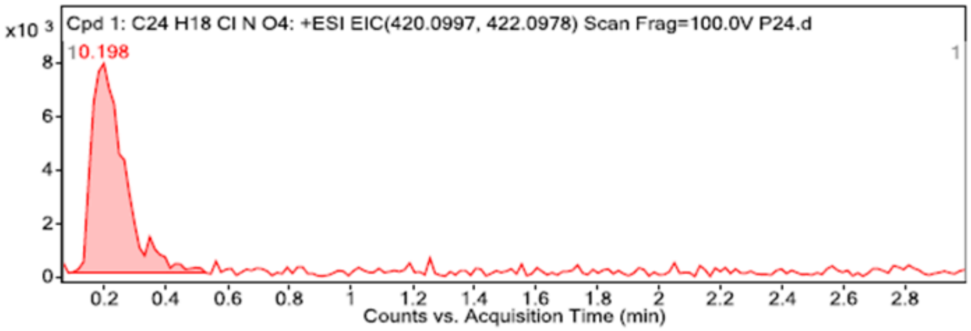

MS Zoomed Spectrum

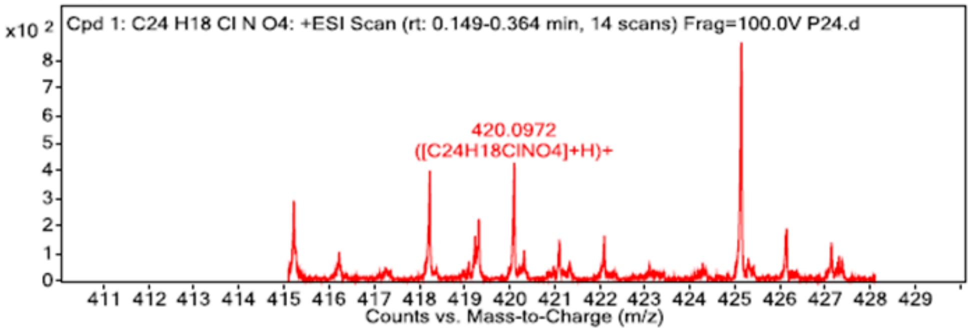

HRMS of (*E*)-4-((3-(((4-chlorophenyl)imino)methyl)phenoxy)methyl)-7-methoxy-2*H*-chromen-2-one **(13j)**

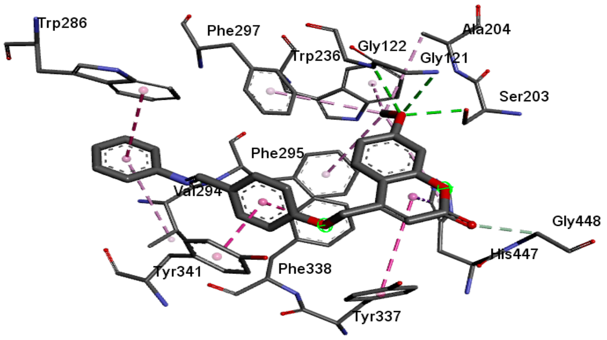

(13a) (3D LIGAND-RECEPTOR)

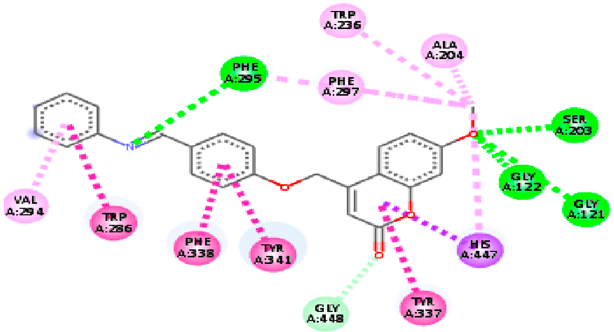

(13a) (2D LIGAND-RECEPTOR)

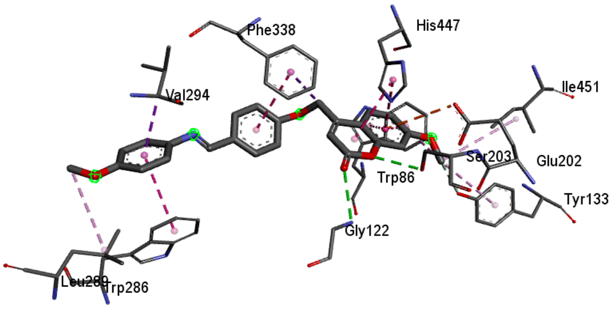

(13b) (3D LIGAND-RECEPTOR)

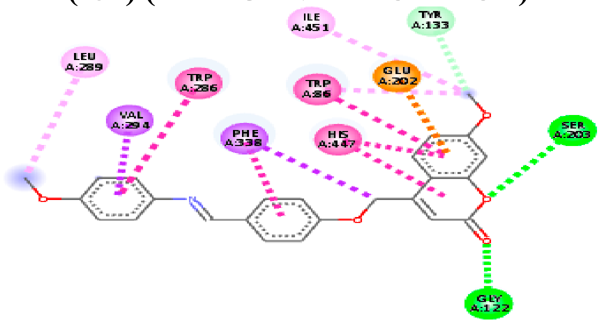

(13b) (2D LIGAND-RECEPTOR)

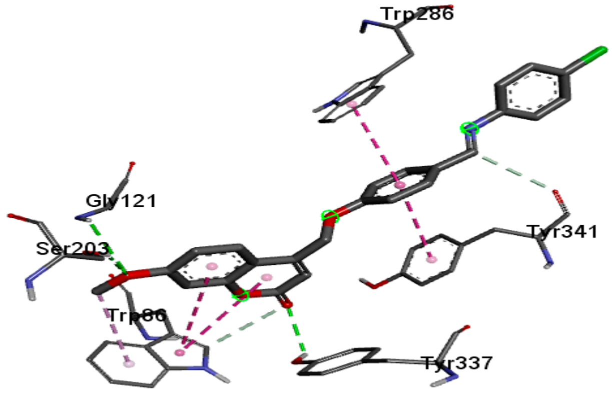

(13c) (3D LIGAND-RECEPTOR)

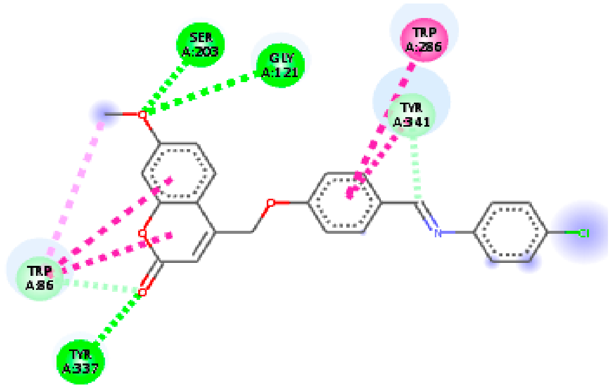

(13c) (2D LIGAND-RECEPTOR)

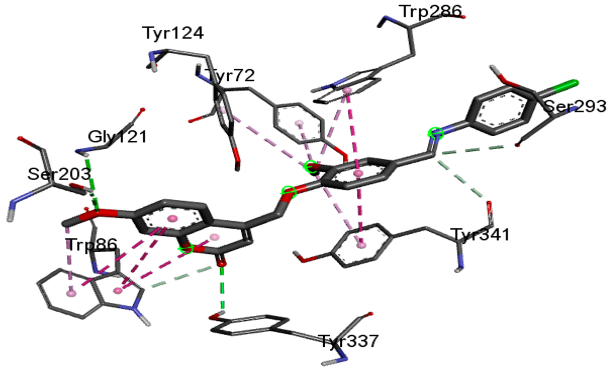

(13d) (3D LIGAND-RECEPTOR)

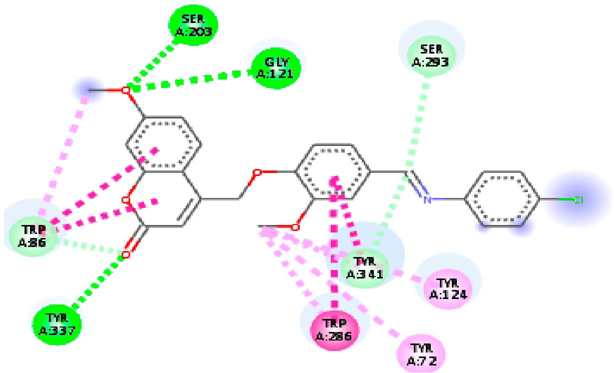

(13d) (2D LIGAND-RECEPTOR)

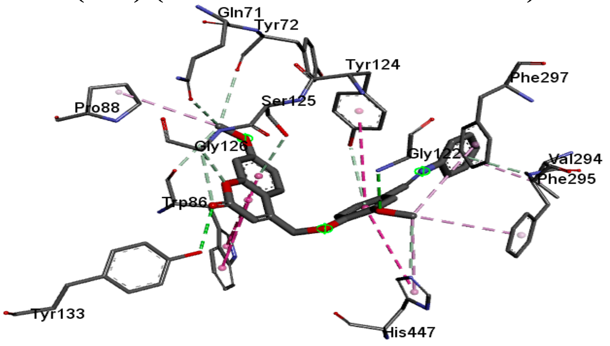

(13e) (3D LIGAND-RECEPTOR)

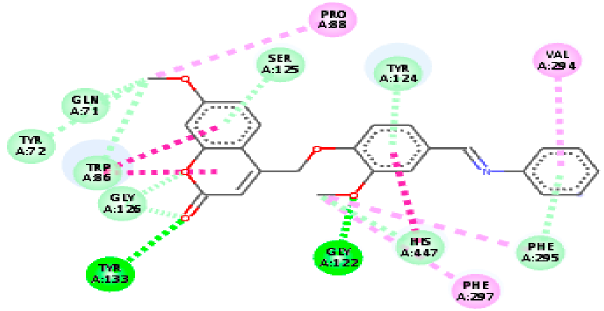

(13e) (2D LIGAND-RECEPTOR)

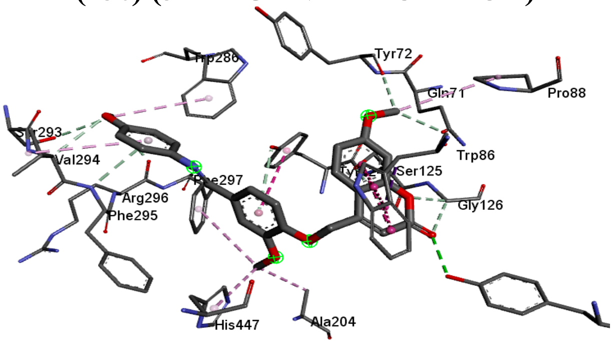

(13f) (3D LIGAND-RECEPTOR)

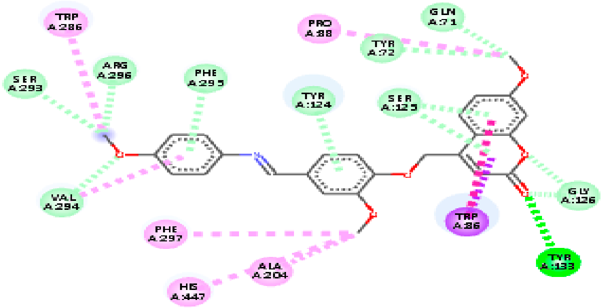

(13f) (2D LIGAND-RECEPTOR)

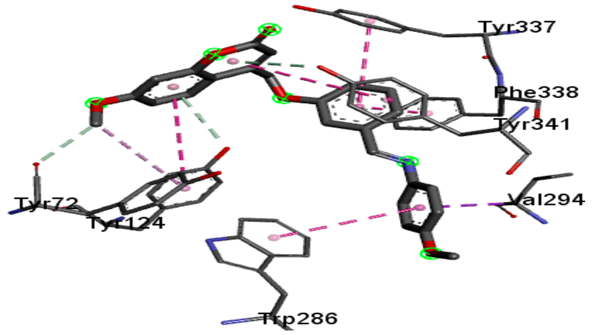

(13g) (3D LIGAND-RECEPTOR)

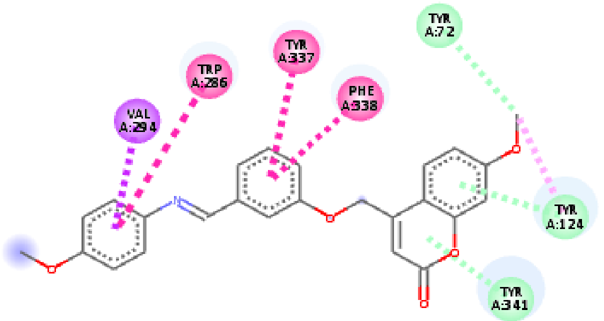

(13g) (2D LIGAND-RECEPTOR)

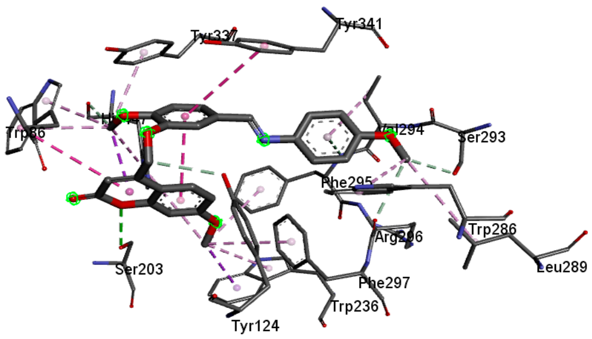

(13h) (3D LIGAND-RECEPTOR)

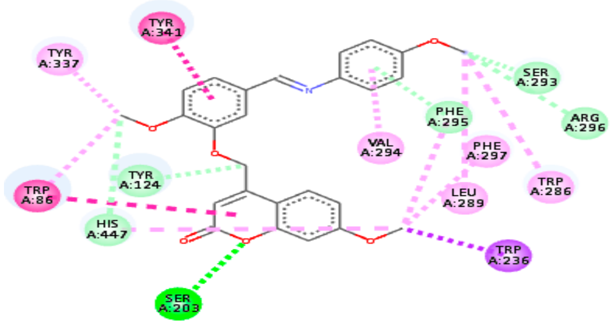

(13h) (2D LIGAND-RECEPTOR)

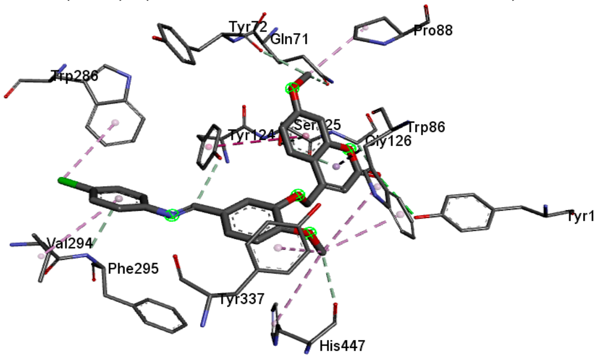

(13i) (3D LIGAND-RECEPTOR)

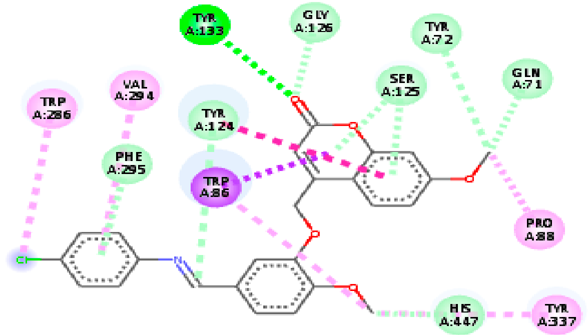

(13i) (2D LIGAND-RECEPTOR)

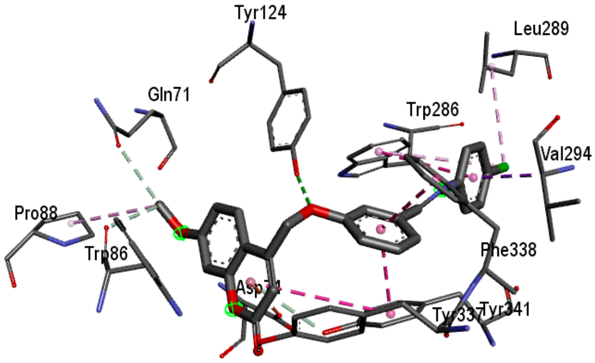

(13j) (3D LIGAND-RECEPTOR)

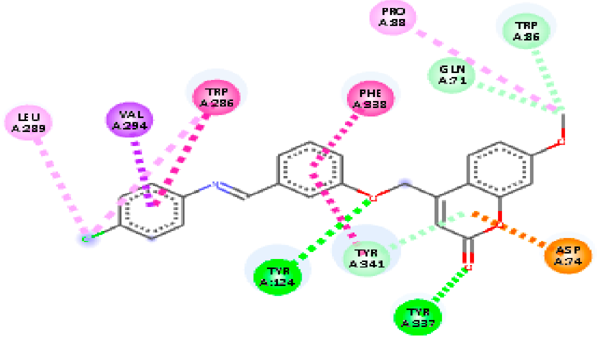

(13j) (2D LIGAND-RECEPTOR)

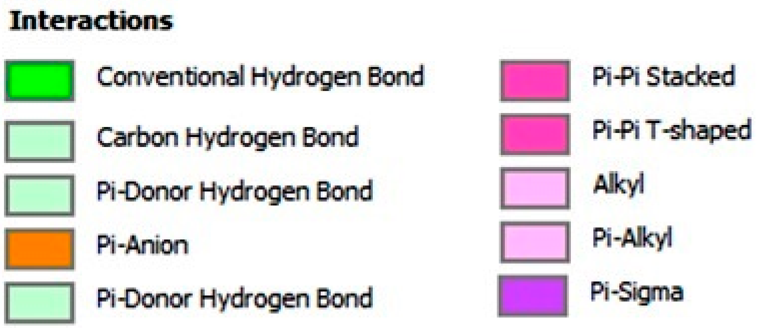

Figure S1. The proposed binding mode of newly synthesized hybrids (**13a-j**) docked in the active site of acetylcholinesterase (4EY7) protein; (2D and 3D ligand-receptor interactions).

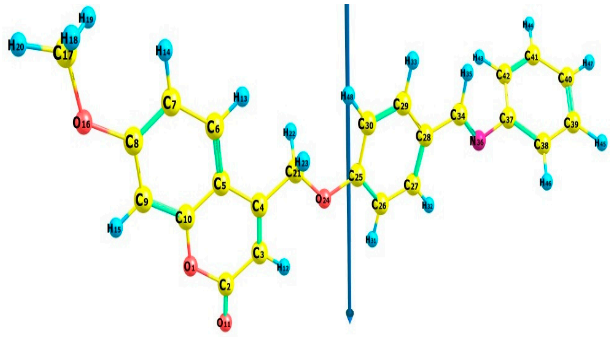

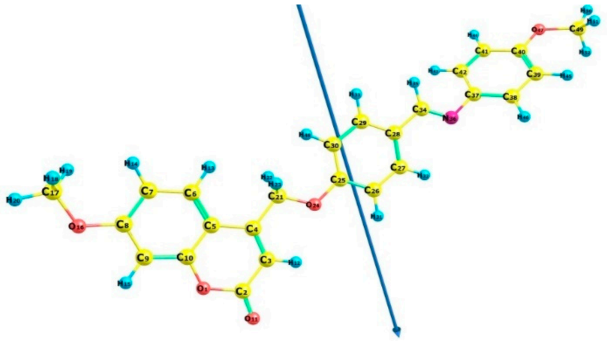

(13a) **(13b)**

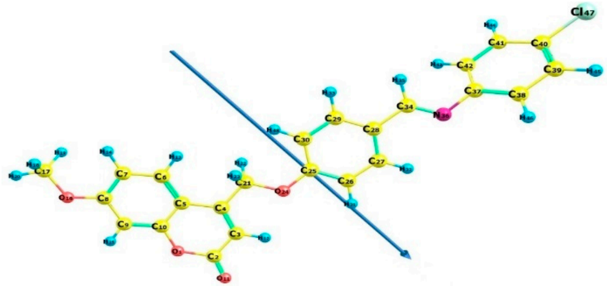

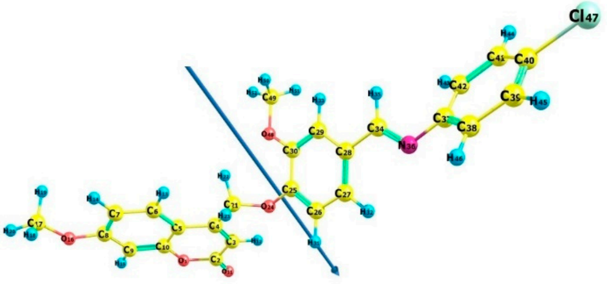

(13c) **(13d)**

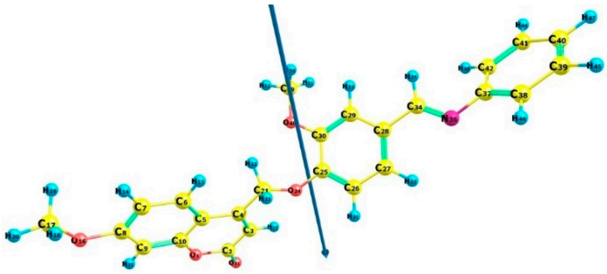

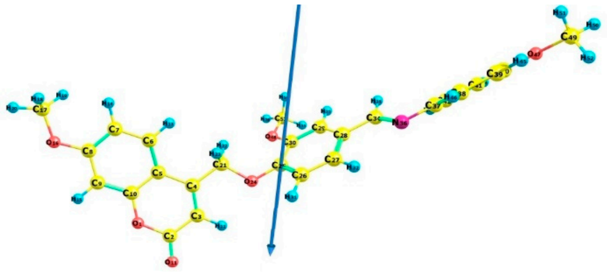

(13e) **(13f)**

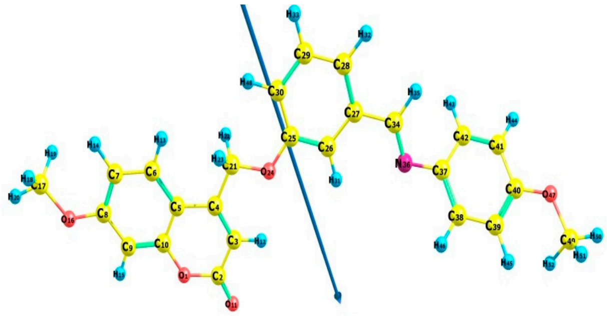

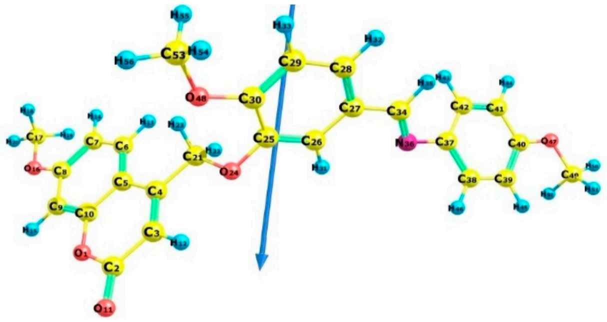

(13g) **(13h)**

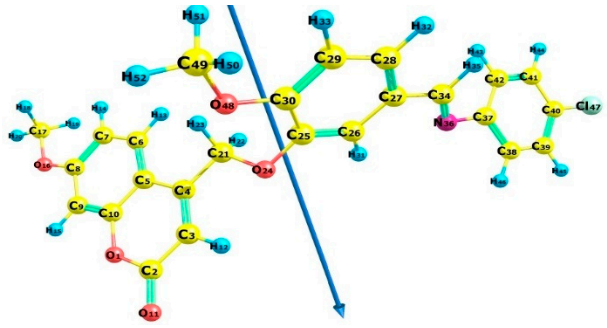

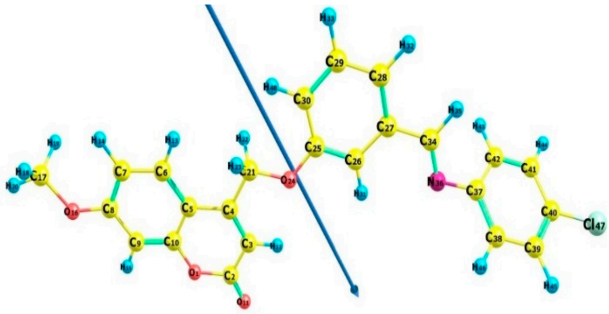

(13i) **(13j)**

Figure S2. The optimized geometry, numbering system, vector of dipole moment of the synthesized compounds (**13a-j)** using wb97xd/6-311++g(d,p) level of calculation.

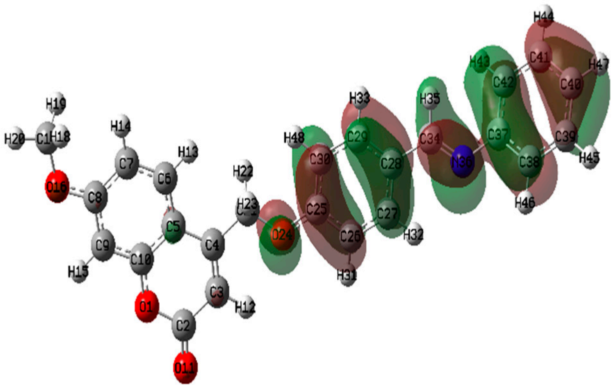

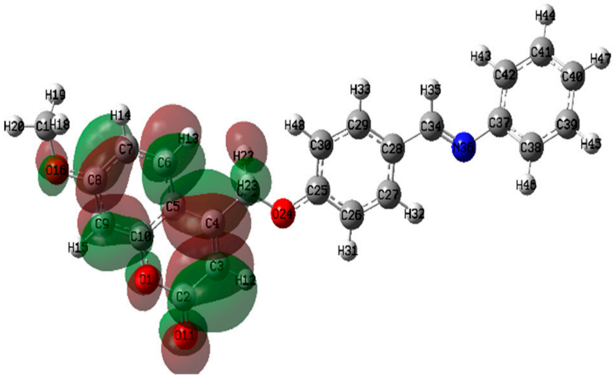

(13a) (HOMO) **(13a) (LUMO)**

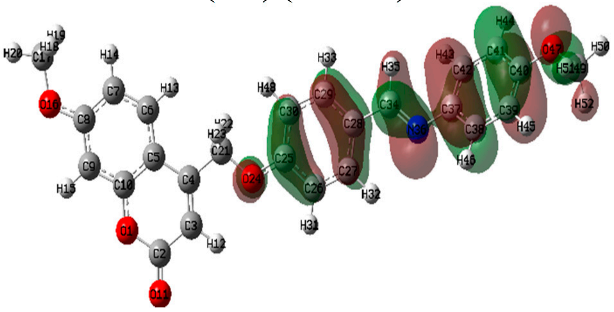

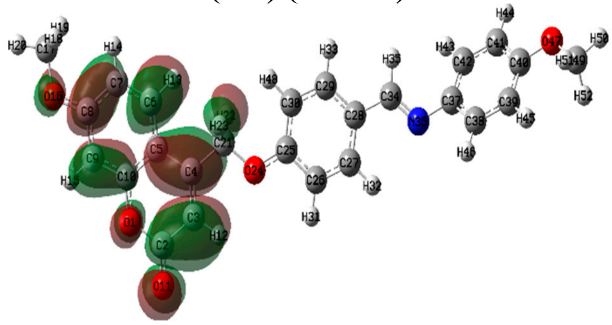

(13b) (HOMO) **(13b) (LUMO)**

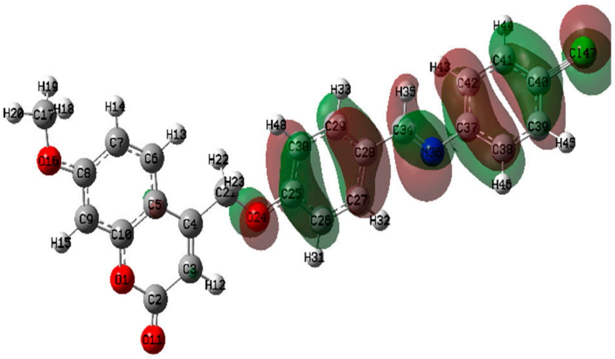

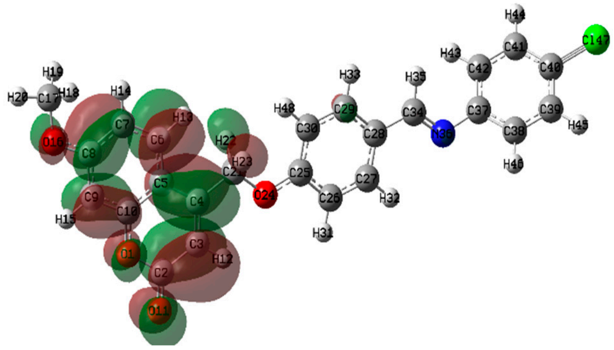

(13c) (HOMO) **(13c) (LUMO)**

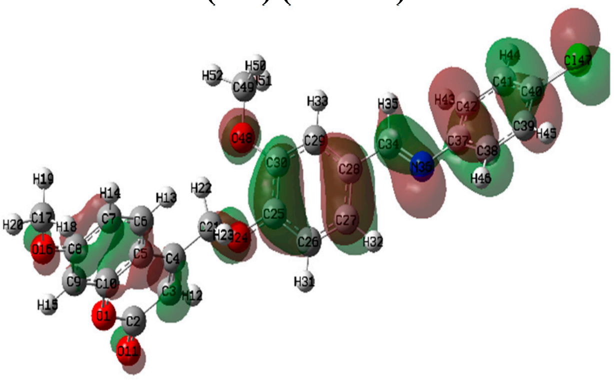

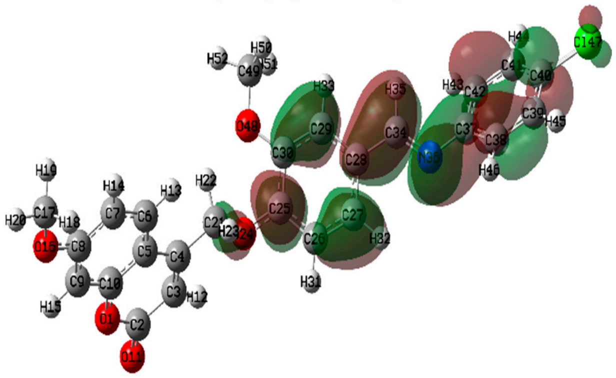

(13d) (HOMO) **(13d) (LUMO)**

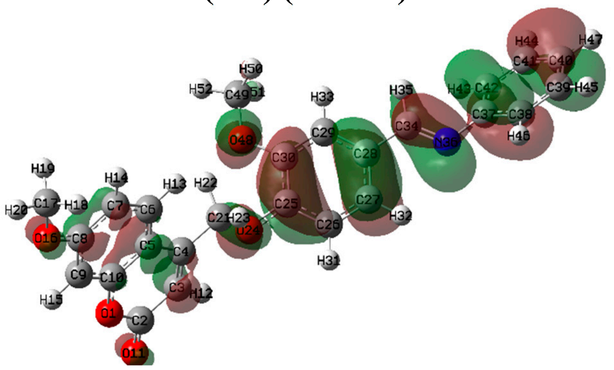

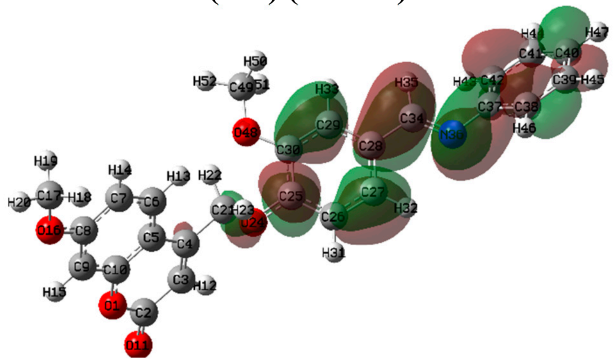

(13e) (HOMO) **(13e) (LUMO)**

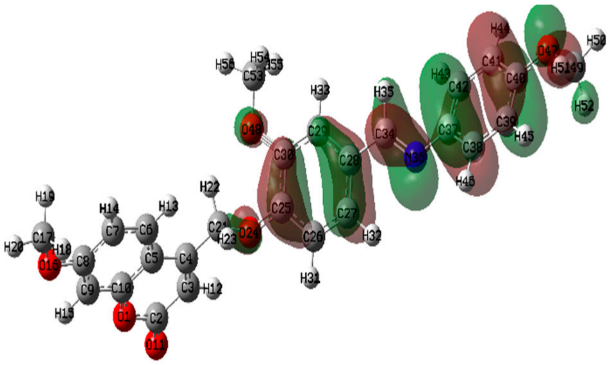

(13f) (HOMO)

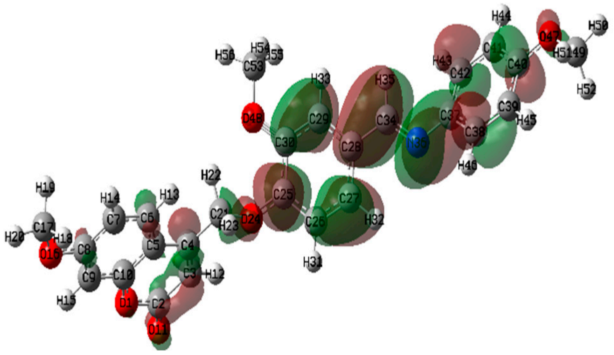

(13f) (LUMO)

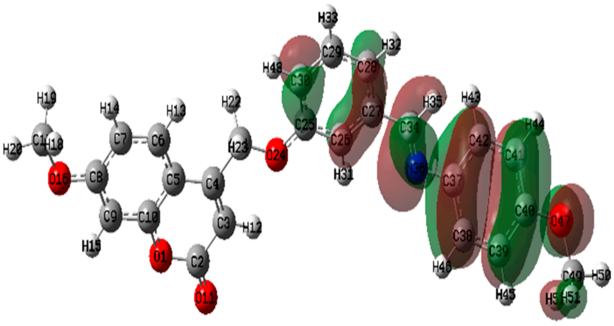

(13g) (HOMO)

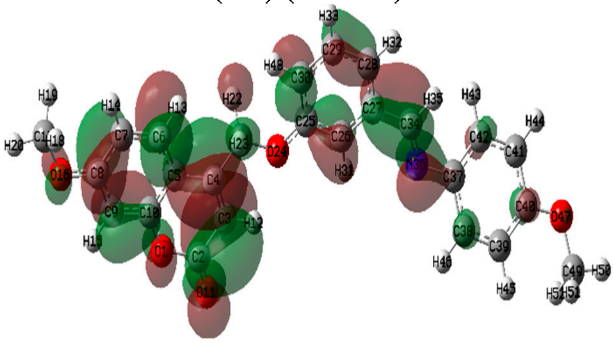

(13g) (LUMO)

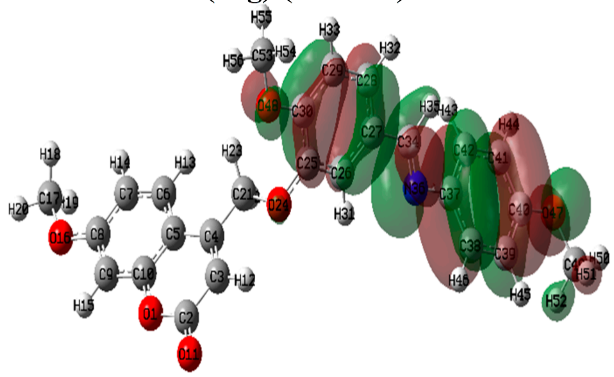

(13h) (HOMO)

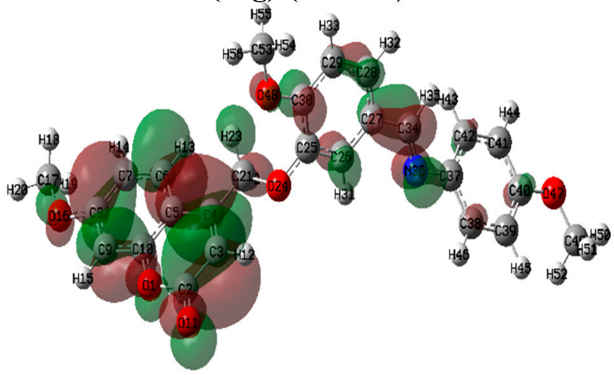

(13h) (LUMO)

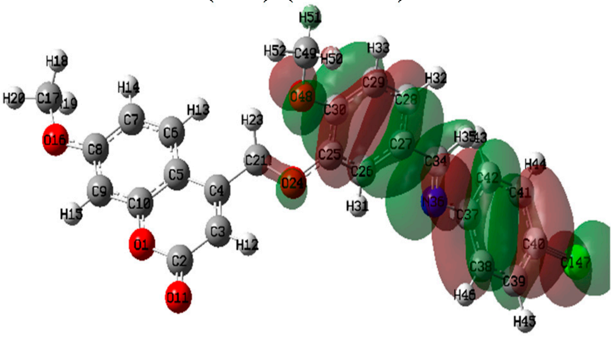

(13i) (HOMO)

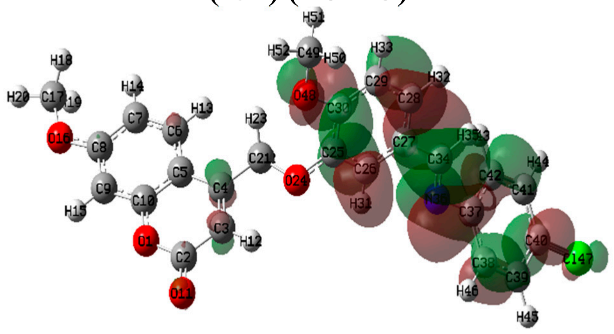

(13i) (LUMO)

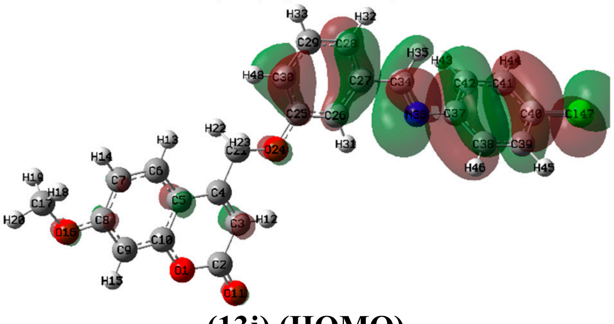

(13j) (HOMO)

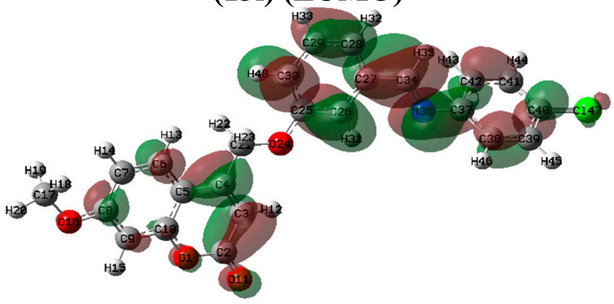

(13j) (LUMO)

Figure S3. Frontier molecular orbitals of the synthesized compounds (**13a-j**) using wb97xd/6-311++g(d,p) level of calculation.

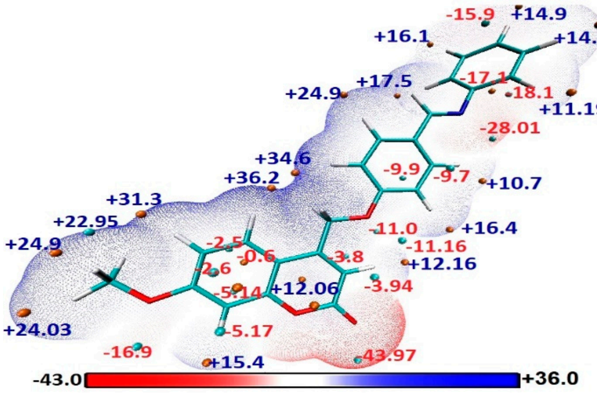

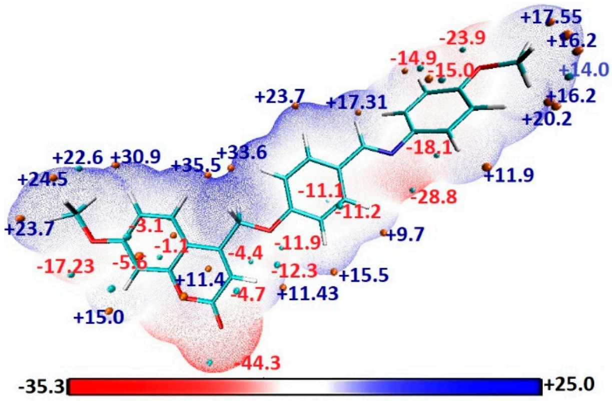

(13a) **(13b)**

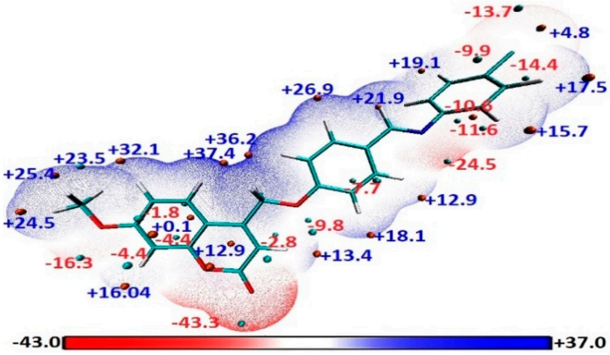

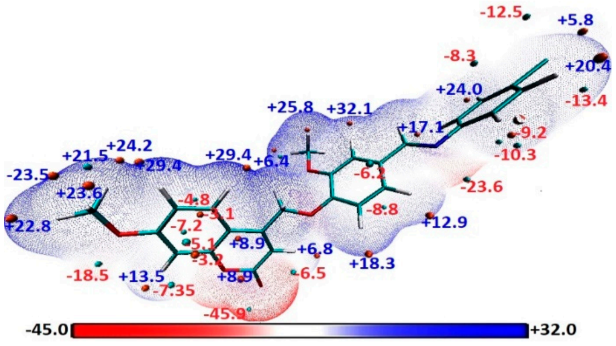

(13c) **(13d)**

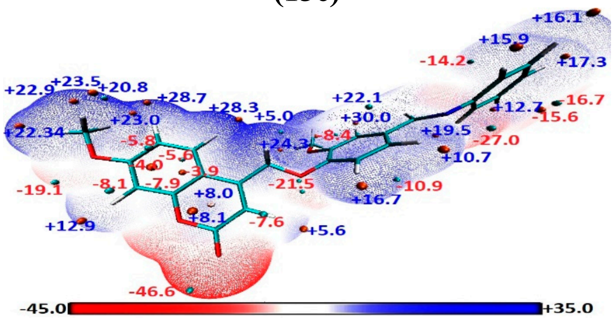

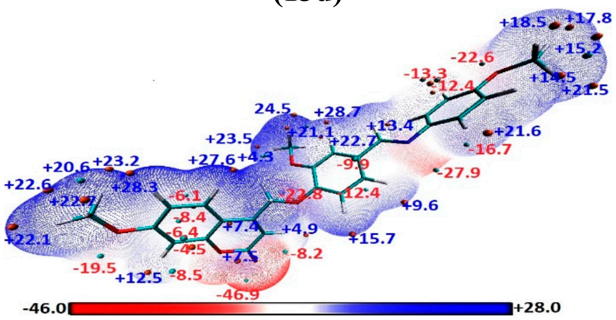

(13e) **(13f)**

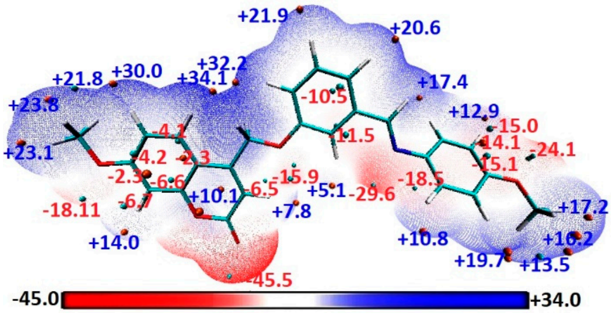

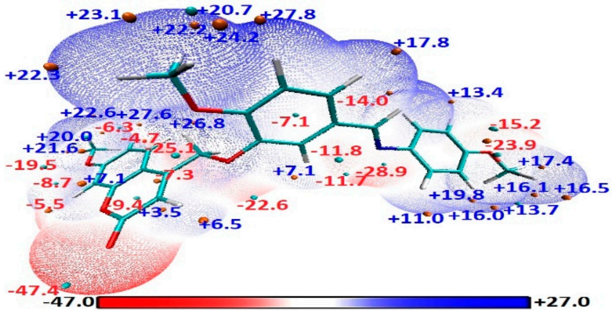

(13g) **(13h)**

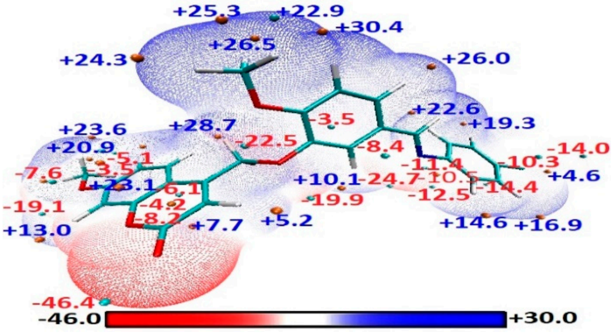

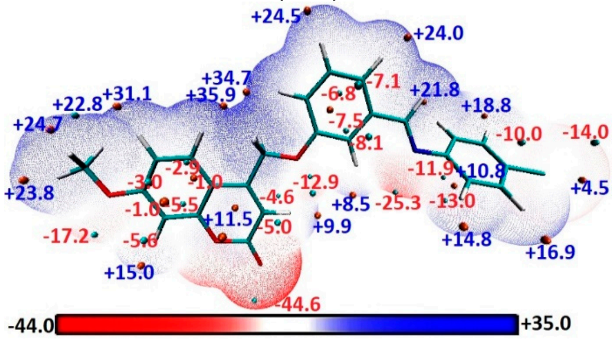

(13i) **(13j)**

Figure S4. MEP surfaces of the synthesized compounds (**13a-j**) using wb97xd/6-311++g(d,p) level of calculation.

Table S1. The selected bond length (A°), bond angles and dihedral angles, (degree) of the newly synthesized compounds (**13a-j**) using wb97xd/6-311++g(d,p) level of level of theory.

	**Exp.** [6]	**13a**	**13b**	**13c**	**13d**	**13e**	**13f**	**13g**	**13h**	**13i**	**13j**
**R(O1,C2)**	1.383	1.380	1.380	1.380	1.381	1.381	1.381	1.381	1.382	1.382	1.381
**R(O1,C10)**	1.360	1.356	1.356	1.356	1.356	1.356	1.356	1.356	1.356	1.356	1.356
**R(C2,C3)**	1.450	1.457	1.457	1.458	1.456	1.456	1.456	1.458	1.456	1.456	1.458
**R(C2,O11)**	1.204	1.198	1.198	1.198	1.198	1.198	1.198	1.198	1.198	1.198	1.198
**R(C4,C21)**		1.501	1.501	1.501	1.499	1.499	1.499	1.502	1.499	1.499	1.502
**R(C8,C9)**	1.379	1.391	1.391	1.391	1.390	1.390	1.390	1.390	1.390	1.390	1.390
**R(C8,O16)**	1.388	1.349	1.349	1.348	1.350	1.350	1.350	1.349	1.350	1.350	1.349
**R(C9,C10)**	1.381	1.383	1.383	1.383	1.384	1.384	1.384	1.384	1.384	1.384	1.383
**R(O16,C17)**	1.363	1.414	1.414	1.414	1.413	1.413	1.413	1.413	1.413	1.413	1.414
**R(C21,O24)**		1.404	1.404	1.405	1.414	1.414	1.414	1.403	1.413	1.413	1.403
**R(O24,C25)**		1.357	1.358	1.356	1.365	1.365	1.366	1.361	1.369	1.367	1.360
**R(C30,A48)**		1.082	1.082	1.082	1.355	1.356	1.356	1.082	1.350	1.349	1.082
**R(C34,N36)**		1.270	1.270	1.271	1.270	1.269	1.270	1.269	1.270	1.271	1.269
**R(N36,C37)**		1.407	1.408	1.405	1.405	1.407	1.408	1.408	1.408	1.405	1.406
**R(C40,A47)**		1.084	1.358	1.749	1.749	1.084	1.357	1.357	1.358	1.749	1.749
**A(C2,O1,C10)**	122.1	122.5	122.5	122.5	122.5	122.5	122.5	122.5	122.5	122.5	122.5
**A(O1,C2,C3)**	117.1	116.5	116.6	116.6	116.5	116.5	116.6	116.6	116.5	116.5	116.5
**A(O1,C2,O11)**	117.4	117.8	117.8	117.8	117.7	117.7	117.7	117.8	117.6	117.6	117.8
**A(C4,C5,C6)**	125.0	125.0	125.0	124.9	124.9	124.9	124.9	124.9	124.9	124.9	124.9
**A(C4,C5,C10)**	117.4	117.6	117.7	117.6	117.7	117.7	117.7	117.7	117.7	117.7	117.6
**A(C7,C8,O16)**		124.2	124.2	124.2	124.2	124.2	124.2	124.2	124.2	124.2	124.2
**A(C8,O16,C17)**	119.1	118.8	118.8	118.8	118.7	118.7	118.7	118.7	118.7	118.7	118.7
**A(C4,C21,O24)**		109.8	109.9	109.9	109.7	109.7	109.7	109.9	110.1	110.0	109.8
**A(C21,O24,C25)**		118.6	118.5	118.5	116.0	115.9	115.9	118.5	114.5	114.8	118.6
**A(O24,C25,C30)**		124.5	124.4	124.4	121.2	121.2	121.2	124.2	119.8	119.9	124.2
**A(C25,C26,C27)**		120.1	120.1	120.1	120.9	120.9	120.9	120.0	120.6	120.6	120.0
**A(C27,C28,C34)**		121.5	121.5	121.5	121.5	121.5	121.6	120.0	119.7	119.8	120.1
**A(C34,N36,C37)**		119.1	119.3	119.1	119.2	119.1	119.6	119.7	119.3	119.2	119.2
**A(N36,C37,C38)**		118.3	118.4	118.4	118.2	118.2	118.1	118.0	118.5	118.4	118.3
**A(C39,C40,C41)**		119.5	119.5	120.9	120.9	119.6	119.5	119.5	119.5	120.9	121.0
**D(C10,O1,C2,O11)**	180.0	179.9	179.8	179.8	-179.9	180.0	-179.9	-179.9	179.6	179.6	-180.0
**D(C2,C3,C4,C21)**	179.1	-179.8	-179.5	-179.6	180.0	180.0	-180.0	179.9	179.5	179.5	-179.9
**D(C5,C4,C21,O24)**		179.8	179.3	-179.5	179.5	179.3	-179.9	179.0	-177.8	-177.6	178.8
**D(C4,C5,C6,C7)**	-179.1	-179.9	-179.8	179.9	-179.9	-179.8	179.9	180.0	-179.9	180.0	179.9
**D(C4,C5,C10,C9)**	179.6	179.9	179.9	-179.9	179.9	179.8	-179.9	-180.0	179.9	-179.9	-179.9
**D(O16,C8,C9,C10)**	178.6	-179.9	-179.8	-179.9	-179.9	-179.8	-180.0	179.9	-179.9	180.0	179.9
**D(C7,C8,O16,C17)**		0.3	0.0	0.4	-1.2	-1.4	-0.8	0.3	-0.3	0.1	0.4
**D(C21,O24,C25,C26)**		179.2	178.7	-179.9	115.1	114.8	115.0	176.5	-107.0	-108.0	177.9
**D(C26,C27,C28,C34)**		180.0	-180.0	-179.8	180.0	-179.9	179.8	180.0	180.0	179.9	-180.0
**D(C27,C28,C34,N36)**		-2.8	-3.9	-3.3	-1.1	-1.0	-0.6	179.9	179.5	179.5	179.8
**D(C28,C34,N36,C37)**		177.6	177.5	177.4	177.5	177.4	177.7	178.3	177.3	177.2	177.9
**D(C34,N36,C37,C42)**		-47.6	-42.2	-47.6	-44.7	-45.1	-38.5	-39.5	-41.8	-46.2	-46.8
**D(N36,C37,C38,C39)**		179.9	-179.7	179.9	-180.0	179.9	-179.6	-179.4	-179.8	179.6	180.0

Values are mean ± SD triplicate assays.

Table S2: Mean absolute errors computed for selected bond lengths (A°) and angles (degree) of synthesized compounds **(13a-j)** verses **7-Acetoxy-coumarin** calculated at long-range corrections wb97xd/6-311++g(d,p) level of theory. The X-ray crystal structure data have been taken from ref. [1]

	13a	13b	13c	13d	13e	13f	13g	13h	13i	13j
**R(O1,C2)**	0.003	0.002	0.003	0.002	0.001	0.001	0.002	0.001	0.001	0.002
**R(O1,C10)**	0.004	0.004	0.004	0.004	0.004	0.004	0.004	0.004	0.004	0.004
**R(C2,C3)**	0.007	0.007	0.008	0.006	0.006	0.006	0.008	0.006	0.006	0.008
**R(C2,O11)**	0.007	0.007	0.007	0.006	0.006	0.006	0.007	0.006	0.006	0.007
**R(C8,C9)**	0.012	0.012	0.012	0.011	0.011	0.011	0.011	0.011	0.011	0.011
**R(C8,O16)**	0.039	0.039	0.039	0.038	0.038	0.038	0.039	0.038	0.038	0.039
**R(C9,C10)**	0.002	0.002	0.002	0.003	0.003	0.003	0.002	0.003	0.003	0.002
**R(O16,C17)**	0.051	0.051	0.051	0.050	0.050	0.050	0.050	0.050	0.050	0.051
**A(C2,O1,C10)**	0.436	0.427	0.434	0.385	0.389	0.375	0.423	0.402	0.414	0.444
**A(O1,C2,C3)**	0.562	0.551	0.549	0.554	0.557	0.548	0.540	0.555	0.561	0.555
**A(O1,C2,O11)**	0.450	0.430	0.462	0.306	0.289	0.273	0.370	0.231	0.263	0.395
**A(C4,C5,C6)**	0.014	0.059	0.069	0.124	0.140	0.132	0.082	0.142	0.123	0.068
**A(C4,C5,C10)**	0.250	0.277	0.271	0.301	0.308	0.313	0.293	0.317	0.301	0.272
**A(C8,O16,C17)**	0.337	0.331	0.325	0.398	0.374	0.433	0.407	0.410	0.392	0.388
**D(C10,O1,C2,O11)**	0.037	0.137	0.148	0.042	0.025	0.040	0.035	0.315	0.352	0.004
**D(C2,C3,C4,C21)**	0.697	0.422	0.545	0.907	0.955	0.904	0.819	0.415	0.468	0.855
**D(C4,C5,C6,C7)**	0.778	0.736	0.843	0.812	0.718	0.841	0.911	0.786	0.890	0.864
**D(C4,C5,C10,C9)**	0.333	0.322	0.338	0.341	0.250	0.390	0.417	0.370	0.359	0.362
**D(O16,C8,C9,C10)**	1.267	1.200	1.314	1.266	1.187	1.353	1.281	1.314	1.354	1.267

Table S3. Values of the Fukui functions and dual descriptor of compounds **(13a-e)** by using wb97xd/6-311++g(d,p) level of theory

	(13a)			(13b)			(13c)			(13d)			(13e)		
**Atom**	**f(-)**	**f(+)**	**Δf**	**f(-)**	**f(+)**	**Δf**	**f(-)**	**f(+)**	**Δf**	**f(-)**	**f(+)**	**Δf**	**f(-)**	**f(+)**	**Δf**
**O1**	0.000	**0.021**	**0.021**	0.000	**0.021**	**0.021**	0.000	0.021	0.020	0.001	0.000	-0.001	0.001	0.000	-0.001
**C2**	0.000	**0.084**	**0.084**	0.000	**0.084**	**0.084**	0.000	0.084	0.083	0.001	0.001	0.000	0.001	0.001	0.001
**C3**	0.004	**0.199**	**0.195**	0.000	**0.198**	**0.198**	0.003	0.198	0.194	0.011	0.007	-0.003	0.007	0.009	0.002
**C4**	0.001	**0.209**	**0.208**	0.000	**0.209**	**0.209**	0.001	0.207	0.206	0.016	-0.003	-0.019	0.018	0.000	-0.017
**C6**	0.001	**0.166**	**0.165**	0.000	**0.167**	**0.167**	0.001	0.164	0.163	0.004	-0.004	-0.008	0.003	-0.003	-0.006
**C8**	0.003	**0.082**	**0.080**	0.000	**0.083**	**0.083**	0.003	0.081	0.078	0.010	-0.001	-0.011	0.008	-0.001	-0.008
**C9**	0.001	**0.052**	**0.051**	0.000	**0.052**	**0.052**	0.001	0.051	0.050	0.004	0.001	-0.003	0.003	0.002	-0.002
**C10**	0.001	**0.035**	**0.034**	0.000	**0.035**	**0.035**	0.001	0.035	0.034	0.005	0.002	-0.002	0.004	0.003	-0.001
**O11**	0.002	0.065	0.063	0.000	**0.065**	**0.065**	0.002	0.065	0.063	0.007	0.000	-0.007	0.006	0.001	-0.005
**O16**	0.002	**0.011**	**0.009**	0.000	**0.011**	**0.011**	0.002	0.011	0.009	0.009	0.000	-0.009	0.007	0.000	-0.007
**C21**	0.001	-0.039	-0.039	0.000	-0.038	-0.039	0.001	-0.040	-0.041	-0.001	0.004	0.005	-0.003	0.002	0.004
**O24**	**0.051**	0.001	**-0.050**	**0.021**	0.001	**-0.019**	0.043	0.001	-0.042	0.029	0.006	-0.023	0.033	0.006	-0.027
**C25**	**0.073**	0.004	**-0.069**	**0.036**	0.004	**-0.033**	0.061	0.004	-0.057	0.093	0.112	0.019	0.109	0.117	0.009
**C26**	**0.027**	0.000	**-0.027**	**0.009**	0.000	**-0.009**	0.022	0.000	-0.022	0.010	0.010	0.000	0.010	0.013	0.004
**C27**	**0.008**	0.001	**-0.008**	**0.011**	0.001	**-0.010**	0.008	0.001	-0.008	0.073	0.085	0.012	0.086	0.089	0.003
**C28**	**0.096**	0.000	**-0.096**	**0.047**	0.000	**-0.047**	0.083	0.001	-0.082	0.068	0.082	0.014	0.077	0.087	0.010
**C29**	**0.037**	0.002	**-0.035**	**0.025**	0.002	**-0.023**	0.031	0.004	-0.027	-0.008	0.075	0.083	-0.010	0.073	0.083
**C30**	**0.040**	0.003	**-0.038**	**0.015**	0.003	**-0.013**	0.034	0.002	-0.031	0.042	0.023	-0.019	0.052	0.025	-0.027
**C34**	**0.021**	0.002	**-0.020**	**0.052**	0.001	**-0.051**	**0.021**	0.005	**-0.016**	0.027	**0.238**	**0.211**	0.029	0.229	0.200
**N36**	**0.141**	0.001	**-0.141**	**0.103**	0.000	**-0.103**	**0.128**	0.002	**-0.126**	0.104	**0.164**	**0.060**	0.114	0.168	0.053
**C37**	**0.129**	0.000	**-0.129**	**0.144**	0.000	**-0.144**	**0.125**	0.000	**-0.125**	**0.092**	0.051	**-0.041**	0.093	0.043	-0.050
**C38**	**0.089**	0.000	**-0.089**	**0.092**	0.000	**-0.092**	**0.078**	0.000	**-0.077**	**0.060**	0.016	**-0.045**	0.067	0.017	-0.050
**C39**	**0.016**	0.000	**-0.016**	**0.055**	0.000	**-0.055**	**0.023**	0.000	**-0.024**	**0.018**	0.001	**-0.017**	0.012	0.006	-0.007
**C40**	**0.138**	0.000	**-0.138**	**0.133**	0.000	**-0.133**	**0.128**	0.001	**-0.128**	**0.101**	0.052	**-0.049**	0.107	0.050	-0.058
**C41**	**0.032**	0.000	**-0.032**	**0.078**	0.000	**-0.078**	**0.039**	0.000	**-0.039**	**0.034**	0.017	**-0.017**	0.029	0.007	-0.022
**C42**	**0.058**	0.000	**-0.058**	**0.038**	0.000	**-0.038**	**0.057**	0.000	**-0.056**	**0.050**	0.032	**-0.018**	0.052	0.029	-0.023
**A47**	0.001	0	-0.001	0.1063	0	-0.1063	0.0796	0.0001	-0.0795	0.0622	0.0066	-0.0556	0.0008	0.0007	-0.0001
**A48**	0.0005	0.0001	-0.0004	0.0002	0.0003	0.0001	0.0004	0.0001	-0.0003	0.0333	0.0006	-0.0327	0.0402	0.0006	-0.0396

A = H, O or Cl *which different atom in derivatives series; Values are mean ± SD triplicate assays.*

Table S4. Values of the Fukui functions and dual descriptor of compounds **(13f-j)** by using wb97xd/6-311++g(d,p) level of theory.

	(13f)			(13g)			(13h)			(13i)			(13j)		
**Atom**	**f(-)**	**f(+)**	**Δf**	**f(-)**	**f(+)**	**Δf**	**f(-)**	**f(+)**	**Δf**	**f(-)**	**f(+)**	**Δf**	**f(-)**	**f(+)**	**Δf**
**O1**	0.000	0.001	0.001	0.000	0.016	0.016	0.000	0.017	0.017	0.000	0.001	0.001	0.000	0.004	0.003
**C2**	0.000	0.005	0.005	0.000	0.063	0.063	0.000	0.073	0.073	0.001	0.003	0.002	0.000	0.013	0.013
**C3**	0.000	0.017	0.017	0.000	0.153	0.153	-0.001	0.163	0.164	-0.001	0.005	0.007	0.008	0.036	0.027
**C4**	0.001	0.009	0.008	0.000	0.162	0.162	0.000	0.183	0.183	0.000	-0.001	-0.001	0.002	0.038	0.037
**C6**	0.001	0.004	0.004	0.000	0.121	0.121	0.000	0.152	0.152	0.001	0.009	0.008	0.001	0.023	0.021
**C8**	0.000	0.002	0.002	0.000	0.062	0.062	0.000	0.073	0.073	0.000	0.002	0.002	0.005	0.012	0.008
**C9**	0.000	0.004	0.004	0.000	0.038	0.038	0.000	0.048	0.048	0.000	0.001	0.001	0.002	0.007	0.005
**C10**	0.000	0.004	0.004	0.000	0.028	0.028	0.000	0.029	0.029	0.000	-0.004	-0.003	0.002	0.007	0.005
**O11**	0.000	0.004	0.004	0.000	0.049	0.049	0.000	0.056	0.056	0.000	0.002	0.002	0.004	0.011	0.007
**O16**	0.000	0.001	0.000	0.000	0.009	0.009	0.000	0.010	0.010	0.000	0.000	0.000	0.004	0.002	-0.002
**C21**	0.007	-0.001	-0.008	0.000	-0.040	-0.040	0.002	-0.036	-0.039	0.005	0.013	0.008	0.000	-0.017	-0.017
**O24**	0.014	0.006	-0.008	0.001	0.002	0.001	0.002	0.001	-0.002	0.007	0.001	-0.006	0.006	0.001	-0.005
**C25**	0.051	0.116	0.065	-0.001	0.003	0.005	0.011	0.005	-0.006	0.029	0.004	-0.025	0.005	0.007	0.003
**C26**	0.003	0.015	0.012	-0.003	0.031	0.034	0.006	0.021	0.015	0.007	0.087	0.080	-0.017	0.098	0.116
**C27**	0.038	0.083	0.045	0.023	0.026	0.003	0.046	0.009	-0.036	0.083	0.074	-0.009	0.031	0.078	0.047
**C28**	0.042	0.087	0.044	0.028	0.007	-0.021	0.030	0.004	-0.026	0.042	0.069	0.027	0.039	0.032	-0.007
**C29**	0.007	0.068	0.062	0.003	0.014	0.010	0.014	-0.002	-0.016	0.031	0.018	-0.013	0.003	0.037	0.034
**C30**	0.018	0.023	0.004	0.029	0.042	0.013	0.042	0.030	-0.012	0.074	0.115	0.041	0.045	0.116	0.072
**C34**	0.055	0.215	0.160	0.079	0.055	-0.024	0.057	0.032	-0.025	0.035	0.235	0.200	0.050	0.197	0.147
**N36**	0.096	0.162	0.066	0.094	0.040	-0.053	0.103	0.024	-0.079	0.125	0.157	0.032	0.126	0.137	0.011
**C37**	0.135	0.041	-0.095	0.159	0.005	-0.154	0.132	0.005	-0.128	0.105	0.051	-0.054	0.151	0.036	-0.115
**C38**	0.083	0.023	-0.059	0.091	0.008	-0.083	0.088	0.007	-0.081	0.071	0.016	-0.055	0.084	0.015	-0.068
**C39**	0.053	-0.002	-0.055	0.065	-0.001	-0.065	0.053	0.000	-0.053	0.022	-0.001	-0.023	0.034	-0.004	-0.037
**C40**	0.128	0.043	-0.085	0.145	0.009	-0.136	0.130	0.006	-0.124	0.120	0.051	-0.069	0.159	0.039	-0.120
**C41**	0.078	0.009	-0.069	0.090	0.002	-0.088	0.079	0.002	-0.077	0.038	0.019	-0.019	0.050	0.012	-0.039
**C42**	0.047	0.022	-0.025	0.042	0.004	-0.038	0.044	0.000	-0.044	0.060	0.025	-0.035	0.068	0.018	-0.049
**A47**	0.102	0.0055	-0.0965	0.1224	0.0012	-0.1212	0.1034	0.0007	-0.1027	0.0731	0.0062	-0.0669	0.1083	0.0045	-0.1038
**A48**	0.0109	0.0007	-0.0102	0.0002	0.0007	0.0005	0.0249	0.0032	-0.0217	0.0532	0.016	-0.0372	0.0003	0.0016	0.0013

A = H, O or Cl *which different atom in derivatives series; Values are mean ± SD triplicate assays.*

Table S5. Values of the condensed local softnesses (Hartree*e) of compounds **(13a-j)** by using wb97xd/6-311++g(d,p) level of theory from CDFT point of view.

Atoms	13a		13b		13c		13d		13e		13f		13g		13h		13i		13j	
	s+/s-	s-/s+	s+/s-	s-/s+	s+/s-	s-/s+	s+/s-	s-/s+	s+/s-	s-/s+	s+/s-	s-/s+	s+/s-	s-/s+	s+/s-	s-/s+	s+/s-	s-/s+	s+/s-	s-/s+
**O1**	0.5	2.0	0.1	7.1	0.2	5.9	0.0	-51.1	1.1	0.9	0.8	1.3	0.6	1.8	0.1	7.3	0.2	4.5	2.7	0.4
**C2**	0.2	5.0	0.0	23.9	0.1	19.0	0.0	26.6	1.0	1.0	0.7	1.5	0.2	4.4	0.0	29.1	0.1	15.7	4.2	0.2
**C3**	0.0	31.6	0.0	1993.5	0.0	186.6	0.0	-35.7	1.2	0.8	0.4	2.8	-1.2	-0.8	0.0	-144.2	0.0	-163.1	225.3	0.0
**C8**	0.5	2.1	0.1	9.4	0.1	7.7	0.1	9.8	1.1	0.9	0.8	1.3	0.6	1.6	0.1	9.9	0.2	5.8	5.1	0.2
**C9**	0.5	2.0	0.1	7.2	0.2	6.0	0.1	8.3	1.1	0.9	0.8	1.3	0.6	1.7	0.1	7.7	0.2	4.7	3.2	0.3
**C10**	0.6	1.7	0.1	8.2	0.2	6.6	0.1	6.9	1.1	0.9	0.8	1.3	0.5	2.1	0.1	10.2	0.2	5.7	9.1	0.1
**O11**	0.4	2.6	0.1	10.6	0.1	8.5	0.1	10.7	1.1	0.9	0.7	1.3	0.3	3.5	0.1	11.0	0.2	6.4	5.8	0.2
**O16**	0.6	1.6	0.2	5.2	0.2	4.3	0.1	18.6	1.1	0.9	0.8	1.2	0.7	1.5	0.2	5.3	0.3	3.4	7.3	0.1
**C21**	0.6	1.7	0.2	4.2	0.3	3.0	0.1	14.4	1.0	1.0	0.6	1.7	0.4	2.5	0.1	10.5	0.2	4.4	0.9	1.2
**O24**	2.1	0.5	-2.4	-0.4	-3.3	-0.3	-1.6	-0.6	1.8	0.6	1.0	1.0	0.7	1.5	-2.6	-0.4	12.9	0.1	0.5	1.8
**C25**	1.0	1.0	-3.1	-0.3	-4.0	-0.3	-2.0	-0.5	1.1	0.9	0.7	1.5	0.5	2.0	-1.7	-0.6	33.9	0.0	0.1	7.4
**C26**	1.2	0.8	20.1	0.0	25.1	0.0	2.6	0.4	0.8	1.2	0.6	1.8	0.3	3.6	7.4	0.1	1.0	1.0	0.1	15.5
**C27**	0.7	1.5	1.6	0.6	2.1	0.5	5.8	0.2	1.2	0.8	0.6	1.7	0.0	-23.0	0.9	1.1	1.8	0.5	0.3	3.3
**C28**	1.1	0.9	0.5	2.2	1.7	0.6	-0.3	-3.1	1.4	0.7	0.2	4.2	0.6	1.6	2.3	0.4	1.8	0.6	0.8	1.3
**C29**	0.9	1.1	4.5	0.2	5.5	0.2	53.7	0.0	0.4	2.4	0.4	2.6	0.4	2.3	2.3	0.4	1.7	0.6	0.3	3.3
**C30**	1.3	0.8	-13.8	-0.1	-19.8	-0.1	0.3	3.6	1.4	0.7	0.6	1.5	0.5	1.9	-7.6	-0.1	2.7	0.4	0.3	3.8
**C34**	0.5	2.2	-27.5	0.0	-60.1	0.0	2.3	0.4	0.3	3.9	0.5	2.2	0.6	1.7	-48.5	0.0	0.9	1.1	0.1	7.5
**N36**	0.9	1.1	3.6	0.3	5.2	0.2	1.0	1.0	0.7	1.5	0.4	2.3	0.4	2.3	8.5	0.1	1.9	0.5	0.2	4.1
**C37**	9.6	0.1	-22.3	0.0	-16.1	-0.1	1.8	0.5	4.5	0.2	22.7	0.0	67.7	0.0	-16.4	-0.1	1321.0	0.0	3.5	0.3
**C38**	2.6	0.4	18.7	0.1	20.1	0.0	42.8	0.0	1.6	0.6	2.0	0.5	2.3	0.4	58.7	0.0	5.7	0.2	0.7	1.4
**C39**	1.5	0.7	9.5	0.1	6.6	0.2	1.8	0.5	1.1	0.9	1.9	0.5	2.3	0.4	10.9	0.1	2.9	0.3	0.6	1.6
**C40**	2.1	0.5	9.6	0.1	9.7	0.1	-0.1	-7.3	1.4	0.7	1.7	0.6	2.0	0.5	9.6	0.1	3.6	0.3	0.8	1.3
**C41**	1.8	0.5	12.6	0.1	9.3	0.1	2.0	0.5	1.2	0.8	2.4	0.4	2.8	0.4	10.8	0.1	3.4	0.3	0.8	1.3
**C42**	2.5	0.4	29.7	0.0	28.1	0.0	36.4	0.0	1.6	0.6	1.8	0.6	2.1	0.5	33.4	0.0	5.1	0.2	0.8	1.3
**A47**	1.5	0.7	15.4	0.1	8.0	0.1	-2.2	-0.5	1.1	0.9	3.4	0.3	4.1	0.2	14.1	0.1	3.8	0.3	0.9	1.1

**A48**	1.2	0.9	-10.4	-0.1	-13.7	-0.1	-1.7	-0.6	2.1	0.5	1.0	1.0	0.5	1.9	-2.3	-0.4	23.8	0.0	0.2	5.5

Table S6. Values of the condensed local electrophilicity (ElP)/nucleophilicity (NuP) index (e*eV) of compounds **(13a-j)** by using wb97xd/6-311++g(d,p) level of theory from CDFT point of view.

**Atoms**	**13a**		**13b**		**13c**		**13d**		**13e**		**13f**		**13g**		**13h**		**13i**		**13j**	
	**ElP**	**NuP**	**ElP**	**NuP**	**ElP**	**NuP**	**ElP**	**NuP**	**ElP**	**NuP**	**ElP**	**NuP**	**ElP**	**NuP**	**ElP**	**NuP**	**ElP**	**NuP**	**ElP**	**NuP**
**O1**	-0.01	-0.01	-0.01	-0.05	-0.01	-0.03	0.00	-0.04	-0.01	-0.01	-0.01	-0.01	0.00	-0.01	0.00	-0.05	-0.01	-0.03	-0.02	-0.01
**C2**	0.00	-0.02	0.00	-0.10	0.00	-0.07	0.00	-0.06	-0.01	0.00	0.00	-0.01	0.00	-0.01	0.00	-0.10	0.00	-0.05	-0.02	0.00
**C3**	0.00	-0.02	0.00	-0.15	0.00	-0.10	0.00	-0.09	0.00	0.00	0.00	0.00	0.00	0.00	0.00	-0.15	0.00	-0.06	-0.08	0.00
**C8**	-0.01	-0.02	-0.01	-0.08	-0.01	-0.05	0.00	-0.05	-0.01	-0.01	-0.01	-0.01	-0.01	-0.01	-0.01	-0.09	-0.01	-0.04	-0.06	-0.01
**C9**	-0.01	-0.02	-0.01	-0.06	-0.01	-0.04	0.00	-0.04	-0.01	-0.01	-0.01	-0.01	-0.01	-0.01	-0.01	-0.07	-0.01	-0.03	-0.03	-0.01
**C10**	0.00	-0.01	0.00	-0.03	0.00	-0.02	0.00	-0.02	0.00	0.00	0.00	0.00	0.00	0.00	0.00	-0.03	0.00	-0.01	-0.03	0.00
**O11**	-0.01	-0.03	-0.01	-0.15	-0.01	-0.10	0.00	-0.10	-0.02	-0.01	-0.01	-0.02	0.00	-0.02	-0.01	-0.16	-0.01	-0.07	-0.09	-0.01
**O16**	-0.01	-0.01	-0.01	-0.04	-0.01	-0.03	0.00	-0.03	-0.01	-0.01	-0.01	-0.01	-0.01	-0.01	-0.01	-0.04	-0.01	-0.02	-0.06	-0.01
**C21**	-0.01	-0.01	0.00	-0.02	-0.01	-0.01	0.00	-0.01	-0.01	-0.01	0.00	-0.01	0.00	-0.01	0.00	-0.02	0.00	-0.01	0.00	0.00
**O24**	-0.04	-0.02	-0.03	0.02	-0.04	0.01	0.00	0.02	-0.04	-0.02	-0.02	-0.03	-0.01	-0.02	-0.01	0.00	-0.01	0.00	-0.01	-0.01
**C25**	-0.05	-0.05	-0.04	0.02	-0.06	0.01	-0.01	0.02	-0.08	-0.07	-0.05	-0.09	-0.02	-0.04	-0.02	0.02	-0.03	0.00	-0.01	-0.02
**C26**	-0.03	-0.03	-0.02	0.00	-0.03	0.00	0.00	0.00	-0.03	-0.03	-0.02	-0.05	-0.02	-0.08	-0.01	0.00	-0.02	-0.02	0.00	-0.05
**C27**	-0.03	-0.04	-0.02	-0.02	-0.03	-0.01	0.00	0.00	-0.07	-0.05	-0.03	-0.07	0.00	-0.06	-0.01	-0.01	-0.04	-0.02	-0.01	-0.03
**C28**	-0.03	-0.03	-0.01	-0.02	-0.03	-0.01	0.00	-0.02	-0.05	-0.03	-0.01	-0.05	-0.02	-0.05	-0.03	-0.02	-0.04	-0.02	-0.03	-0.03
**C29**	-0.04	-0.04	-0.03	-0.01	-0.04	-0.01	0.00	0.00	-0.02	-0.04	-0.02	-0.06	-0.02	-0.07	-0.02	-0.01	-0.03	-0.02	-0.02	-0.04
**C30**	-0.04	-0.03	-0.02	0.00	-0.04	0.00	0.00	-0.01	-0.05	-0.03	-0.02	-0.04	-0.04	-0.10	-0.04	0.01	-0.05	-0.02	-0.02	-0.06
**C34**	-0.04	-0.09	-0.05	0.00	-0.05	0.00	0.00	-0.01	-0.03	-0.11	-0.05	-0.15	-0.06	-0.14	-0.05	0.00	-0.04	-0.04	-0.02	-0.09
**N36**	-0.08	-0.08	-0.05	-0.02	-0.08	-0.01	0.00	-0.02	-0.07	-0.10	-0.04	-0.14	-0.04	-0.13	-0.04	-0.01	-0.07	-0.03	-0.03	-0.08
**C37**	-0.05	0.00	-0.08	0.00	-0.06	0.00	-0.01	-0.03	-0.03	-0.01	-0.07	0.00	-0.09	0.00	-0.07	0.01	-0.04	0.00	-0.02	0.00
**C38**	-0.06	-0.02	-0.06	0.00	-0.06	0.00	-0.01	0.00	-0.05	-0.03	-0.06	-0.04	-0.07	-0.04	-0.06	0.00	-0.05	-0.01	-0.02	-0.02
**C39**	-0.04	-0.02	-0.05	-0.01	-0.04	0.00	-0.01	-0.02	-0.03	-0.03	-0.05	-0.03	-0.05	-0.03	-0.04	-0.01	-0.03	-0.01	-0.02	-0.02
**C40**	-0.09	-0.04	-0.08	-0.01	-0.08	-0.01	0.00	-0.06	-0.08	-0.05	-0.07	-0.06	-0.08	-0.05	-0.07	-0.01	-0.06	-0.02	-0.03	-0.03
**C41**	-0.04	-0.02	-0.06	-0.01	-0.04	0.00	-0.01	-0.02	-0.04	-0.03	-0.06	-0.03	-0.07	-0.03	-0.05	-0.01	-0.04	-0.01	-0.02	-0.02

**C42**	-0.05	-0.02	-0.04	0.00	-0.05	0.00	-0.01	0.00	-0.04	-0.02	-0.04	-0.03	-0.04	-0.03	-0.04	0.00	-0.04	-0.01	-0.02	-0.02
**A47**	-0.04	-0.03	-0.08	-0.01	-0.14	-0.01	-0.05	0.17	-0.04	-0.03	-0.07	-0.03	-0.08	-0.03	-0.07	-0.01	-0.12	-0.03	-0.07	-0.05
**A48**	-0.02	-0.02	-0.02	0.00	-0.02	0.00	0.00	0.00	-0.04	-0.02	-0.02	-0.02	-0.02	-0.05	-0.03	0.02	-0.05	0.00	-0.01	-0.03

Values are mean ± SD triplicate assays

References

1. Z. Yang, D. Zhang, J. Ren, M. Yang, and S. Li. Acetylcholinesterase inhibitory activity of the total alkaloid from traditional Chinese herbal medicine for treating Alzheimer’s disease. Medicinal Chemistry Research. 21(6) (2012) 734-738. https://doi.org/10.1007/s00044-011-9582-8.
2. A.H. Hasan, S. Shakya, F.H.S. Hussain, S. Murugesan, S. Chander, M.R.F. Pratama, S. Jamil, B. Das, S. Biswas, and J. Jamalis. Design, synthesis, anti-acetylcholinesterase evaluation and molecular modelling studies of novel coumarin-chalcone hybrids. Journal of Biomolecular Structure and Dynamics. (2023) 1-13. https://doi.org/10.1080/07391102.2022.2162583.
3. A.H. Hasan, S. Murugesan, S.I. Amran, S. Chander, M.M. Alanazi, T.B. Hadda, S. Shakya, M.R.F. Pratama, B. Das, S. Biswas, and J. Jamalis. Novel thiophene Chalcones-Coumarin as acetylcholinesterase inhibitors: Design, synthesis, biological evaluation, molecular docking, ADMET prediction and molecular dynamics simulation. Bioorganic Chemistry. 119 (2022) 105572. https://doi.org/10.1016/j.bioorg.2021.105572.
4. A. Abdalla Ali, S.A. Mhamad, A.H. Hasan, I. Ahmad, S.A. Abdullah, S. Jamil, H. Patel, S. Murugesan, and J. Jamalis. Synthesis, biological evaluation and molecular modeling studies of modulated benzyloxychalcones as potential acetylcholinesterase inhibitors. Journal of Biomolecular Structure and Dynamics. (2023) 1-12. https://doi.org/10.1080/07391102.2023.2220032.
5. H.A. Hasan, S.M.F. Yusof, A.N. Kamarudin, S. Murugesan, S. Shakya, and J. Jamalis. Synthesis, Anti-acetylcholinesterase Evaluation, Molecular Docking and Molecular Dynamics Simulation of Novel Psoralen Derivatives. Current Organic Synthesis. 20 (2023) 1-17. http://dx.doi.org/10.2174/1570179420666230328121554.
6. K. Gnanaguru, N. Ramasubbu, K. Venkatesan, and V. Ramamurthy. A study on the photochemical dimerization of coumarins in the solid state. The Journal of Organic Chemistry. 50(13) (1985) 2337-2346. https://doi.org/10.1021/jo00213a027.
